# Area-Selective Atomic/Molecular Layer Deposition of Europium-Organic Thin Films on Graphene and Other 2D Materials for Photoluminescent Heterostructures

**DOI:** 10.1021/acsnano.5c22728

**Published:** 2026-03-17

**Authors:** Aleksei V. Emelianov, Kamila K. Mentel, Amr Ghazy, Joona Pekkanen, Yu-Han Wang, Andreas Johansson, Maarit Karppinen, Mika Pettersson

**Affiliations:** § Nanoscience Center, Department of Chemistry, 4168 University of Jyväskylä, FI-40014 Jyväskylä, Finland; † Department of Chemistry and Materials Science, School of Chemical Engineering, Aalto University, FI-00076 Aalto, Espoo, Finland; ‡ Nanoscience Center, Department of Physics, University of Jyväskylä, FI-40014 Jyväskylä, Finland

**Keywords:** graphene, 2D material, femtosecond laser, area-selective deposition, luminescence

## Abstract

Developing a controlled, defect-free, and spatially selective deposition of molecular and hybrid thin films on 2D materials remains a key challenge for their integration into multifunctional optoelectronic systems due to their surface inertness. Here, we demonstrate area-selective atomic/molecular layer deposition of europium-organic (Eu-BDC) thin films on graphene utilizing direct femtosecond laser two-photon oxidation. The laser dose defines the density of nucleation sites and precisely controls Eu-BDC film thickness and uniformity. By optimizing the deposition parameters and carefully choosing a transfer polymer, we achieve over 90% selectivity and high film homogeneity in the activated areas with submicron resolution. Upon 532 nm excitation, graphene/Eu-BDC exhibits strong emission at 612 nm with additional lines at 579, 592, and 652 nm. It also shows a green band at ∼566 nm, which is not observed on Si/SiO_2_. Photoluminescence quenching on graphene shortens lifetimes due to energy and charge transfer at the graphene/Eu-organic interface. Moreover, the Eu-organic layer lowers the graphene work function and shifts the Dirac point, indicating a controllable n-type doping. The same laser modification strategy is also demonstrated on other 2D materials, as shown for MoS_2_ and WS_2_. This resist-free approach enables area-selective growth on 2D surfaces with tunable optical and electronic properties, providing compact integration of patterned emitters and photodetectors on a single chip.

## Graphical abstract

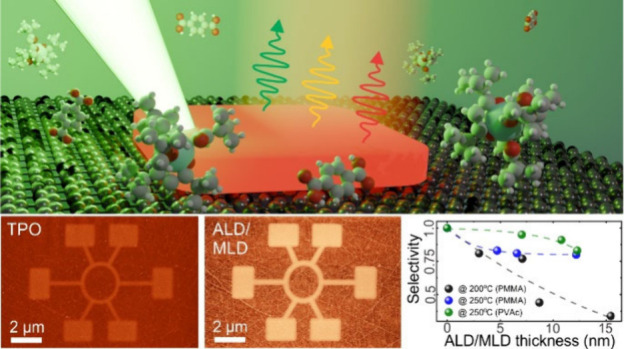

## Introduction

Graphene heterostructures and 3D architectures, intricately patterned and combined with various functional materials, exhibit diverse properties and hold significant potential for advancing opto- and bioelectronics, sensors, and energy conversion and storage technologies.^1–7^ Current patterning techniques mainly employ wet chemistry, focus on 2D functionalities, and do not allow vertical stacking in desired places.^8,9^ Atomic layer deposition (ALD) and molecular layer deposition (MLD) are useful tools to control thin film growth on different surfaces.^10–12^ Both methods provide uniform layer-by-layer growth over large areas, while MLD uses organic precursors instead of inorganic or metal-based reactants.^13^ The modularity of these techniques enables their combination into a process known as atomic/molecular layer deposition (ALD/MLD). This combined approach has been shown to produce metal–organic thin films with enhanced mechanical and optoelectronic properties.^11,14,15^

Area-selective deposition (ASD) is increasingly being explored as a promising alternative to conventional top-down methods,^16^ offering the advantage of reducing edge placement errors.^17^ ASD enables thin film growth predominantly on designated surfaces,^18^ even when multiple different surfaces are present under identical processing conditions.^19^ To maximize the effectiveness of ASD, it is crucial to differentiate between growth areas (GA) and nongrowth areas (NGA) by using bottom-up process technology to achieve high selectivity.^20^

Area-selective ALD (AS-ALD) and MLD (AS-MLD) have already been employed on many materials,^21–24^ including the growth of 2D materials (2DM).^25,26^ Utilizing inhibitors to suppress the deposition in undesired areas is the dominant method in current ASD,^27–29^ however, it requires additional lithographic steps.^30^ Due to the inherent 2D nature, the surface of 2DM does not provide sufficient reactive sites for the chemisorption of ALD/MLD precursors compared with traditional microelectronics.^31,32^ While AS-ALD has been demonstrated on 2DM,^33^ its combination with AS-MLD remains largely unexplored. Functionalization of distinct surface areas is required to allow the selective growth of materials. Selectivity can be enhanced using surface functionalization by self-assembled monolayers,^34,35^ electron beam irradiation,^36,37^ UV exposure,^38^ oxygen plasma treatment,^39–41^ or by growing lateral superlattices.^42^ However, surface activation methods such as electron beam irradiation may introduce lattice defects and trap states as well as amorphous carbon contamination, compromising area selectivity.

Recently, we proposed a way to overcome the chemical inertness of graphene to ALD precursors by locally activating the surface using direct femtosecond laser (fs-laser) two-photon oxidation (TPO) for area-selective ZnO deposition.^43^ This method photochemically attaches oxygen-containing groups to graphene by exposing it to ultrafast laser pulses under an ambient atmosphere.^44,45^ As a result, epoxy and hydroxyl groups form on the graphene surface with moderate laser exposure, while carbonyl and carboxyl groups appear at higher irradiation doses, closer to the ablation threshold.^46,47^ TPO provides precise control over the oxidation level of graphene and enables the patterning of complex structures with high spatial resolution (∼300 nm), with the potential to achieve below 20 nm resolution using tip-enhanced techniques.^48,49^ Additionally, this is a simple, resist-free ultrafast direct laser writing (UDLW) method,^50^ that works in ambient air, and the oxidized graphene surface can be restored to its initial state through thermal annealing in an inert atmosphere.^43^

Here, for the first time, we perform AS-ALD/MLD on the 2DM surface with submicron resolution in a controllable manner, utilizing the chemical interactions of precursors with the TPO surface and restoring graphene to its initial state. Europium-organic thin films deposited via ALD/MLD show interesting photoluminescence properties, which are difficult to achieve using typical ALD of europium-oxide.^51–54^ We combined UDLW and ASD to develop luminescent graphene/Eu-organic heterostructures in predefined areas using TPO. We achieved high homogeneity and over 90% selectivity in locally activated graphene regions for Eu-organic films up to 11 nm. The fabricated graphene/Eu-organic thin films exhibited high photoluminescence emission even when excited with a continuous wave (CW) 532 nm laser, while Eu^3+^ ions typically require UV excitation. These ALD/MLD films can also act as inhibitors for subsequent depositions,^55,56^ allowing for the addition of new functionalities to graphene-based heterostructure devices.

## Results and Discussion

### Area-Selective ALD/MLD on Graphene

We used the Eu­(thd)_3_ and 1,4-benzene dicarboxylic acid (BDC) precursors to deposit Europium-1,4-benzene dicarboxylate (Eu-BDC) films by AS-ALD/MLD on graphene, as illustrated in Scheme 1. To overcome the inertness of the graphene surface on SiO_2_ ^31–33^ and increase the selectivity of the deposition in the target areas, we utilized our recently developed TPO method via fs-laser irradiation in an ambient atmosphere.^44,57^ Using this method, we introduced C–O–C and −OH groups at low and moderate laser doses, and −COOH and −C=O groups at high doses (see Table S1).^46,58^ Following the successful functionalization of selected 2 × 2 um^2^ areas with oxygen-containing groups, we deposited 3–15 nm Eu-BDC films on the 18 graphene chips using ALD/MLD (Table S2). We varied the number of ALD/MLD cycles and temperature to achieve the desired thickness. The choice of Eu-BDC thin films was made as this is a well-studied ALD/MLD process, where the growth is well controlled.^51,59,60^ For graphene transfer, we used two different polymers: poly­(methyl methacrylate) (PMMA) and polyvinyl acetate (PVAc). All the ALD/MLD films were amorphous, as deduced from grazing incidence X-ray diffraction measurements on Si.^60^

**Scheme 1:**
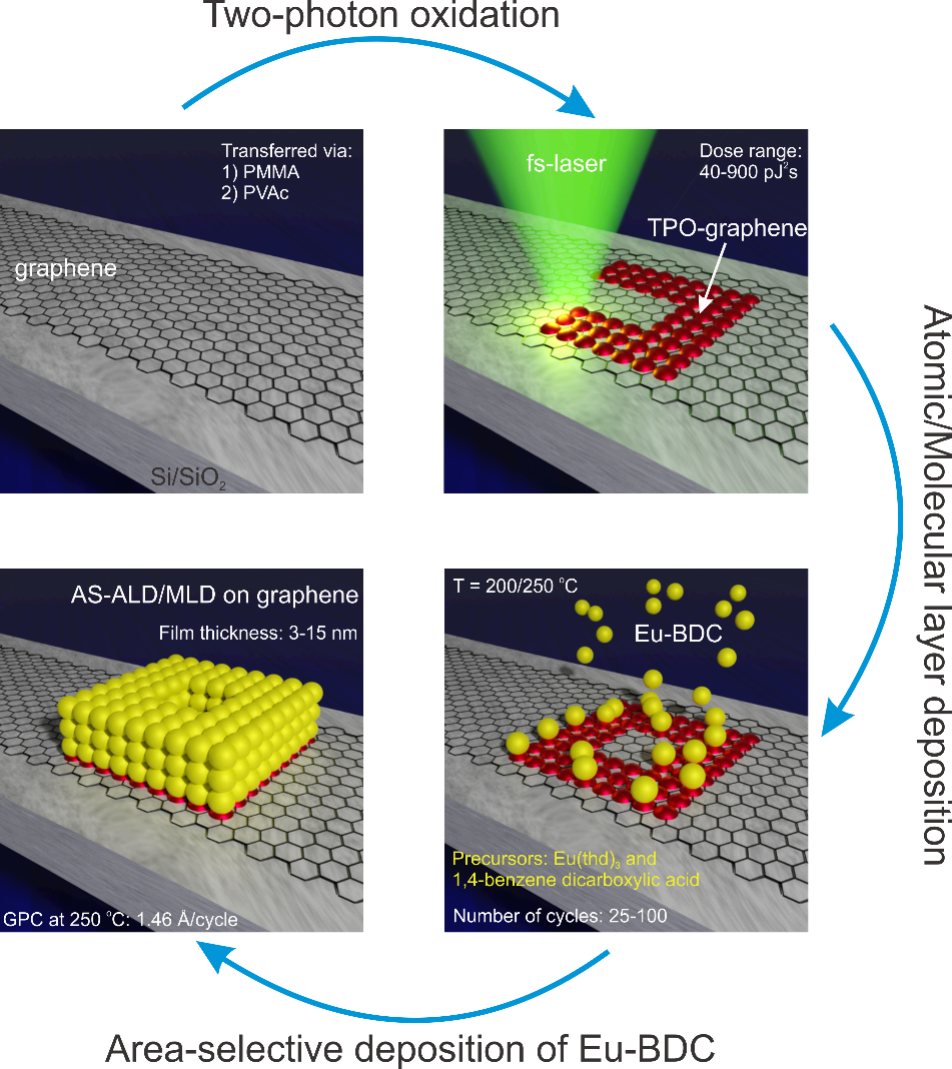
Process Flow for AS-ALD/MLD of Eu-BDC Thin Films on Graphene Using UDLW^a^

a The red spheres represent oxygen-containing surface functional groups, and the yellow spheres show Eu-BDC species selectively deposited on the TPO-activated regions.

AFM images of PVAc-transferred graphene (PVAc graphene) after TPO (Figure 1a) and 50 ALD/MLD cycles of Eu-BDC at 250 °C (Figure 1b) show selective deposition on predefined 2 × 2 μm^2^ areas with UDLW. The thickness of the Eu-BDC films had a little variation with the laser dose and was ∼7.1 nm for 158 pJ^2^s, while deposition on pristine graphene occurred only on wrinkles and polymer residues, with an average thickness of 0.19 ± 0.1 nm (Figure 1c). When the deposition was performed on PMMA-transferred samples (PMMA graphene) at 250 °C (Figure S1), the Eu film thickness was ∼6.6 nm at 158 pJ^2^s, however, the average thickness on the pristine graphene surface increased to 0.71 ± 0.3 nm. The loss of selectivity due to surface contamination by polymer residues was more pronounced for AS-ALD/MLD deposition performed at a lower temperature of 200 °C (Figure S1). The increased height (1.5–2 nm) of the TPO graphene arises from both surface oxidation and localized structural deformation. At the applied fs-laser doses, TPO is primarily photochemical, however, minor photothermal effects can induce out-of-plane rippling of graphene on Si/SiO_2_, increasing the measured thickness. In addition, oxygen-containing groups form on both sides of the graphene sheet, further contributing to the resulting height.

**Figure 1:**
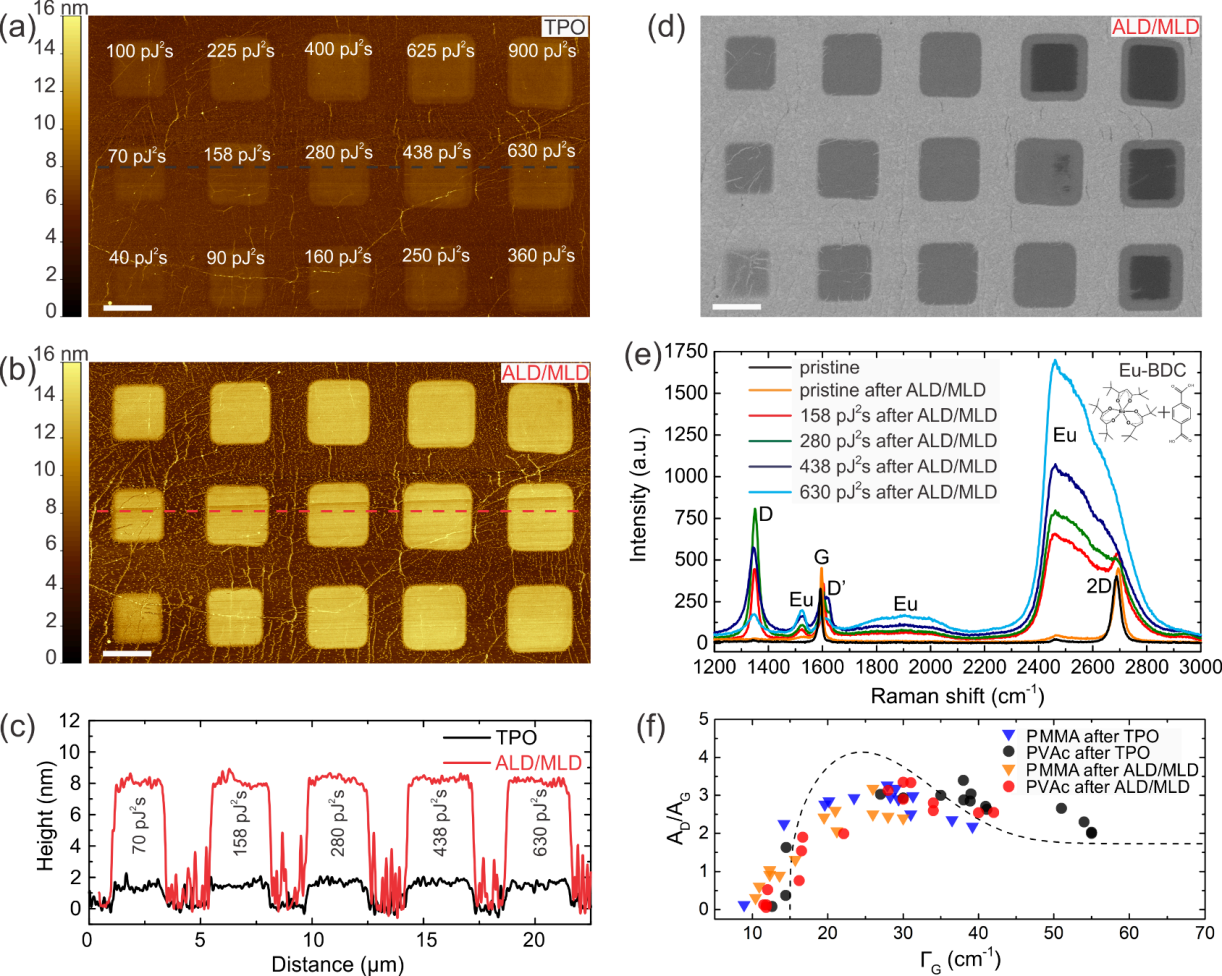
a) AFM image of PVAc graphene after TPO with various fs-laser irradiation doses. b) AFM image of the same sample after 50 ALD/MLD cycles of Eu-BDC. c) AFM profiles before and after ASD measured from a) and b). d) HIM image after ALD/MLD. The scale bars in a), b), and d) are 2 μm. e) Raman spectra of pristine graphene before and after 50 ALD/MLD cycles of Eu-BDC, and of four TPO areas after 50 ALD/MLD cycles of Eu-BDC. f) A_D_/A_G_ vs Γ_G_ after TPO and ALD/MLD on PVAc and PMMA graphene. The dashed line represents the simulation curve corresponding to the generated point-like defects.

Secondary electron images obtained using a helium ion microscope (HIM) after ALD/MLD of Eu-BDC show a smooth distribution of europium compound in TPO areas with sharp edges, and minimal growth in pristine graphene regions (Figure 1d). At low laser doses (<100 pJ^2^s), the film roughness in TPO areas is affected by wrinkles. A clear threshold for laser-induced damage was observed at ∼438 pJ^2^s, where graphene maintained its integrity and could be partially or fully restored during the ALD/MLD process. At higher pulse energies, exceeding 25 pJ (for 360, 625, 630, and 900 pJ^2^s), irreversible structural defects and conductivity loss due to an increased amount of oxygen species were observed, as seen from the HIM image.

We used Raman spectroscopy to probe graphene functionalization and further investigate the selectivity of Eu-BDC AS-ALD/MLD on graphene. Figure 1e demonstrates that only a small blue shift of the G (∼1590 cm^–1^) and 2D (∼2680 cm^–1^) bands was observed after the deposition of a thin Eu-BDC film on pristine graphene. This shift is most probably caused by thermal annealing at 250 °C during the ALD/MLD process. In the TPO areas, showing an increased D (∼1350 cm^–1^) band and an additional D′ (∼1625 cm^–1^) band due to the oxidation process (see Figure S2),^57^ three additional bands appeared after the Eu-BDC deposition (Figure S3). Two bands at ∼1900 cm^–1^ (corresponding to 592 nm emission wavelength) and ∼2450 cm^–1^ (corresponding to 612 nm) are associated with electronic transitions of the Eu-BDC film,^60^ and their intensity increases with the laser dose, which correlates with the thickness and homogeneity of the film. A third band at ∼1525 cm^–1^ (579 nm) also corresponds to an electronic transition in Eu-BDC. The FTIR spectrum of a 100 nm Eu-BDC film deposited on a Si substrate (see Figure S4) shows two main bands at ∼1394 cm^–1^ and ∼1545 cm^–1^, corresponding to the asymmetric and symmetric stretching of the bonded carboxylate groups near the Eu^3+^ ion.^62^ While the asymmetric stretching of the carboxylate group is close to the third band in the Raman spectrum at ∼1525 cm^–1^, it is not Raman active^63^ but can be activated via surface-enhanced Raman spectroscopy with the appropriate substrate.^64^

We also carried out nano-FTIR spectroscopy on pristine graphene and graphene/Eu-BDC films via infrared scattering scanning near-field optical microscopy to study the distribution of Eu-BDC on graphene at the nanoscale. While pristine graphene shows no IR bands in the region of interest, the graphene/Eu-BDC film demonstrates two distinct peaks at ∼1402 cm^–1^ and ∼1562 cm^–1^ (Figure S5), similar to those observed for a thicker Eu-BDC film on Si. When comparing transmission FTIR and nano-FTIR spectra, the band positions may shift by a few wavenumbers. However, a more significant shift may indicate stronger electronic interactions between graphene and the thin Eu-BDC film compared to the film on Si.

To assess the crystallinity of graphene and its variation after TPO and ALD/MLD, we calculated the A_D_/A_G_ ratios and defect concentration (n_D_) from the measured Raman spectra (Figure S6 and Table S3). Figure 1f shows the A_D_/A_G_ ratio as a function of the full width at half-maximum (Γ_G_) of the G band for PVAc and PMMA graphene before and after TPO and 50 ALD/MLD cycles of Eu-BDC. The defects generated in graphene by UDLW correspond to point-like defects.^65^ After the ALD/MLD process, a decrease in the A_D_/A_G_ ratio and a narrowing of Γ_G_ were observed, especially for PMMA graphene films. Thermal annealing at 250 °C during the ALD/MLD partially restored the laser-induced point-like defects and improved the crystallinity of graphene. Additionally, the chemical interaction between the graphene and Eu-BDC film weakened due to the reduced number of oxygen-containing defects on the surface. Thus, even if defects are generated in graphene upon UDLW, the intrinsic properties of the layer can be restored during ALD/MLD at 250 °C when small to moderate fs-laser irradiation doses are used. At higher laser pulse energies (>25 pJ), it becomes harder to control the defect density, which can result in an increase of n_D_ after the ALD/MLD process (Figure S6b). We should note that the reduction and broadening of the D band at TPO doses ≥ 438 pJ^2^s does not indicate less oxidation, but rather reflects the stage where vacancy and hole defects start to dominate, and I_D_/I_G_ decreases and saturates with increasing disorder.^66^ These structural modifications enhance precursor adsorption, leading to the growth of thicker Eu-organic films.

### What Affects the Selectivity of ALD/MLD on Graphene?

A schematic illustration of the interaction between ALD/MLD precursors and pristine graphene, as well as graphene with PMMA and PVAc residues, is shown in Figure 2a. The thickness of the ALD/MLD Eu-BDC films demonstrated slight variation with the laser dose for PVAc graphene layers within 50 cycles (Figure 2b). However, a small variation in thickness was observed for 100 cycles and doses below 70 pJ^2^s. Graphene transferred via PMMA demonstrated a higher dependence on the laser dose, with thickness saturation occurring only after 100 pJ^2^s for 50 cycles and 360 pJ^2^s for 100 cycles, respectively. This variation can be attributed to the presence of polymer residues, particularly for PMMA, which may inhibit the oxidation of graphene at low fs-laser doses and reduce the number of functional groups in the TPO area. In our further analysis, we used the heights of the ALD/MLD films measured within the laser dose range of 225–438 pJ^2^s, where all studied samples showed saturation of film thickness (see Figure 2b).

**Figure 2:**
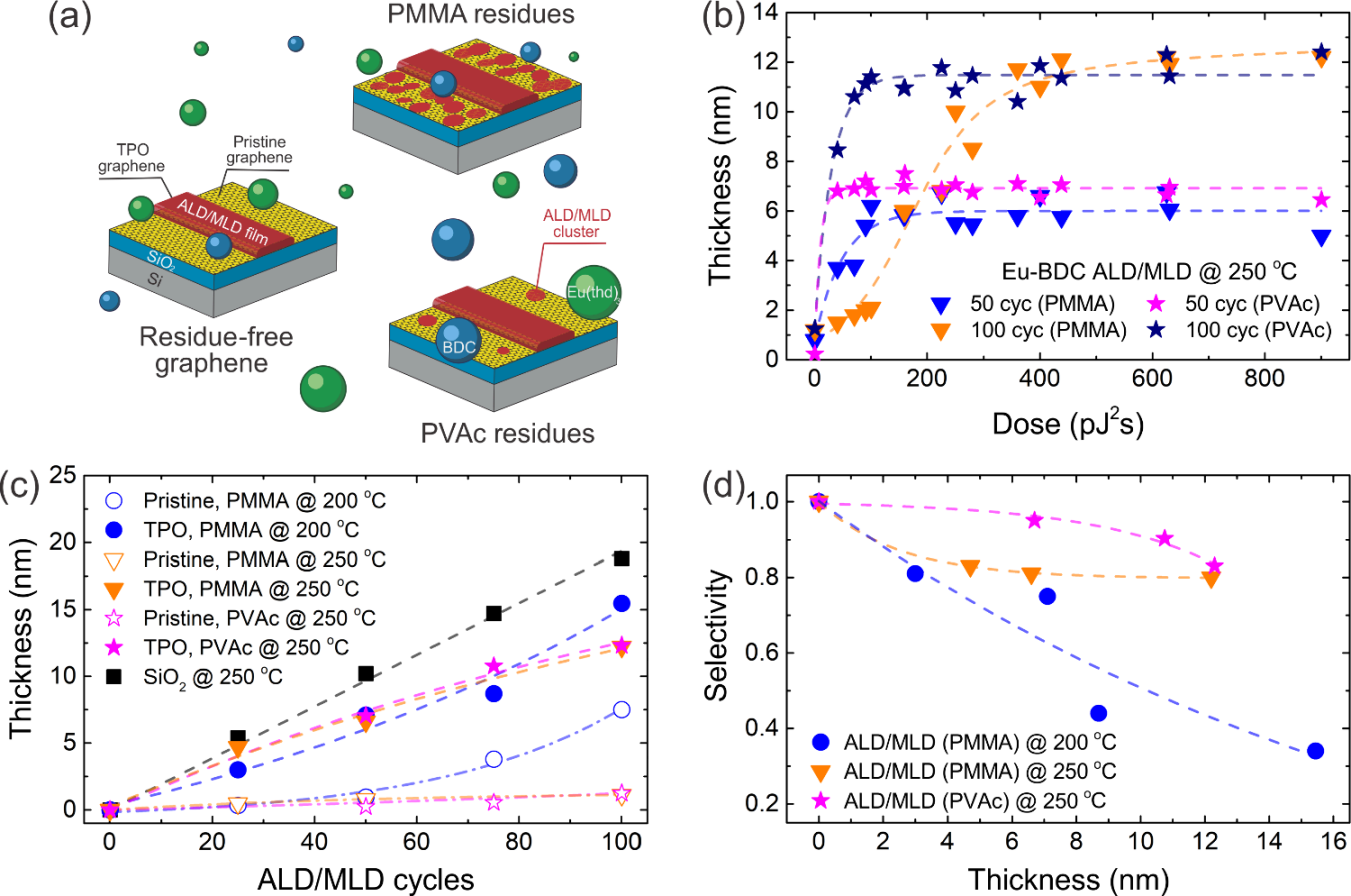
a) Schematic illustration of ALD/MLD precursor interaction on pristine graphene, and graphene with PMMA and PVAc residues. b) Thickness vs fs-laser dose for PMMA and PVAc graphene samples after 50 and 100 ALD/MLD cycles of Eu-BDC at 250 °C. c) Thickness vs number of ALD/MLD cycles, measured for TPO graphene in the laser dose range of 225–438 pJ^2^s and for pristine graphene areas. d) Selectivity vs thickness, measured in the 225–438 pJ^2^s dose range. Dashed lines are guides to the eye.

Figure 2c shows the Eu-BDC film thickness measured on SiO_2_ and graphene using AFM as a function of ALD/MLD cycles. The growth on the SiO_2_ surface demonstrates a linear dependence, however, the growth per cycle (GPC) of ∼1.94 ± 0.03 Å/cyc at 250 °C is slightly lower than on the Si substrate (see Methods). We found that the Eu-BDC film grows on TPO graphene without a noticeable nucleation delay, and all the samples exhibit nearly linear growth within the first 75 cycles, with the GPC of ∼1.45 ± 0.02 Å/cyc (PVAc at 250 °C), ∼1.30 ± 0.12 Å/cyc (PMMA at 200 °C), and ∼1.47 ± 0.17 Å/cyc (PMMA at 250 °C). After 75 cycles, the GPC of the Eu-BDC film grown on TPO PMMA graphene at 200 °C increases, while for films grown at 250 °C, GPC saturation is observed. GPC saturation after 75 cycles is also visible for thin films grown on TPO PVAc graphene. The thicknesses of the deposited films for the same number of ALD/MLD cycles differ between TPO graphene and the SiO_2_ substrate due to the more chemically inert surface of graphene.

The average thickness measured on pristine graphene after the ALD/MLD process at 250 °C shows a nucleation delay of at least ∼50 cycles for PVAc graphene. Growth on PMMA graphene at 200 °C occurred already after 25 cycles and demonstrates exponential behavior, whereas at 250 °C, growth is suppressed due to a weaker interaction between the precursor molecules and the pristine graphene surface.

The selectivity of the deposition can be assessed by measuring the thickness of the Eu-BDC films on TPO and pristine graphene.^19^ To demonstrate the selectivity of our method, we tested four different film thicknesses, two transfer polymers, and two different temperatures. The selectivity of ALD/MLD was calculated as

$$Selectivity=\frac{\theta_{GA}-\theta_{NGA}}{\theta_{GA}+\theta_{NGA}}$$

where *θ*_*GA*_ and *θ*_*NGA*_ are the amount of material present after ALD/MLD on the growth and nongrowth areas, respectively.^67^ The selectivity of the ALD/MLD process at 250 °C for PVAc graphene films exceeds 0.9 up to 11 nm (Figure 2d). Selectivity ≥ 0.9 is typically considered high in ASD^19,68^ and is sufficient for device fabrication with a clean heterostructure interface.^35^ While PMMA graphene samples demonstrate lower selectivity than PVAc chips for thicknesses below 12 nm, the difference vanishes as the Eu-BDC film thickness increases.

When the temperature decreases from 250 to 200 °C, selectivity drops due to the increased interaction of reactive precursor molecules with the pristine graphene surface. A significant decrease in selectivity was observed, dropping from 75% at 5 nm to 35% at 15 nm. Nucleation on wrinkles and other defects also reduces the selectivity of ASD on CVD graphene, as previously demonstrated for HfO_2_.^69^ The differences in selectivity between PMMA- and PVAc-transferred samples can be attributed to their distinct residue chemistry and its interaction with ALD/MLD precursors. Based on our observations, PVAc residues are less reactive and form fewer nucleation sites after graphene transfer, whereas PMMA residues promote precursor adsorption through residual hydrocarbons. The effect of temperature further modulates these interactions. At 200 °C, Eu-BDC grows much faster on PMMA particles and graphene wrinkles. However, at 250 °C, a strong chemisorption occurs only on oxygen-functionalized regions, which significantly improves the selectivity (see Figure 2d). In addition, adsorbed impurities from the air can enhance ALD/MLD nucleation in pristine graphene areas. We did not test ALD/MLD at temperatures exceeding 300 °C due to the partial decomposition of oxygen-containing groups in TPO areas^43,70^ and the reduced reactivity of precursor molecules with the surface,^71^ which could drastically decrease the selectivity and GPC of ALD/MLD. Therefore, deposition at temperatures above 250 °C and below 300 °C on TPO graphene areas is required for AS-ALD/MLD of Eu-BDC films with selectivity exceeding 0.9. Alternatively, less reactive precursors could be used to perform the deposition at lower temperatures.

To achieve more conformal coverage on the pristine surfaces of 1D and 2DM, the physical adsorption of precursor molecules can be utilized at lower temperatures.^72,73^ However, nonspecific physical adsorption compromises the selectivity of ALD and MLD. This challenge can be addressed by utilizing the physisorption and diffusion of ALD precursors in superlattice-based ASD, as demonstrated for MoS_2_ (blocking area) and MoSe_2_ (deposition area) crystal structures.^42^ In our case, the ASD process is driven by chemisorption. Annealing during ALD/MLD restores the graphene surface at low and moderate fs-laser doses, reducing the chemical interaction with the film, as observed in the Raman spectra (see Figures 1f and S3). At moderate to high doses, the interaction between graphene and Eu-BDC films remains strong. By carefully tuning the fs-laser dose, the interaction strength can be controlled. A weaker interaction is preferable for transistor applications, while a stronger interaction is advantageous in designing graphene-based heterostructures that require enhanced electron transfer between graphene and functional films, for example, in optoelectronics and photonics.

We also studied how the preannealing step affects the selectivity of ALD/MLD deposition on graphene. Annealing in an Ar/H_2_ atmosphere reduced the amount of PMMA residues and enhanced the selectivity for Eu-BDC deposition (see Figures 2, S7, and Table S2). Annealing in an O_2_ atmosphere increased the number of defects in the pristine graphene layer and promoted the growth of the Eu-organic film outside the TPO areas. For PVAc graphene, the result was the opposite. While ALD/MLD on the unannealed graphene surface resulted in a nucleation delay of at least ∼50 cycles, for annealed PVAc graphene samples in an Ar/H_2_ atmosphere, the selectivity dropped to 0.7 already after 25 cycles. This may be due to the decomposition of PVAc and the presence of amorphous carbon after annealing at 350 °C in an inert atmosphere. Thus, we argue that the interaction of precursor molecules with PVAc is much weaker compared to PMMA and amorphous carbon, resulting in fewer unwanted nucleation centers for ALD/MLD (Figure 2a).

Although UDLW is a maskless method, polymer residues are an unavoidable challenge in wet-transferred graphene^74,75^ and can strongly affect the nucleation behavior in AS-ALD/MLD. While plasma or UV-ozone cleaning can remove such residues, these treatments typically damage graphene and disrupt its electronic structure. For our samples, we did not observe a continuous polymer layer after graphene transfer, as indicated by AFM (Figure S8) and Raman data (Figure S3). Figure 1a and 1b show that Eu-BDC film growth on pristine graphene occurred only on residues already visible before the ALD/MLD process and on graphene wrinkles. To address this issue, employing dry transfer methods or depositing on freshly grown graphene can be effective solutions.^33,61^ These residues often enhance nucleation during the ALD and MLD processes on 2DM, and selecting a precursor that interacts less strongly with commonly used polymers could be a promising area for further research.

To demonstrate the generality of the AS-ALD/MLD process, we applied TPO and Eu-BDC ALD/MLD to monolayer MoS_2_ and WS_2_. In both materials, selective film deposition occurred mostly in the TPO regions, confirming that this approach effectively works on inert 2D surfaces. The corresponding AFM images, Raman maps and spectra, including the evolution of the E^1^_2g_ and A_1g_ bands and excitonic features, are provided in the Supporting Information (Figures S9 and S10).

In this work, the achieved >90% selectivity and consistent film growth across 18 samples indicate that trace residues do not significantly influence the TPO and AS-ALD/MLD. The introduction of oxygen groups on TPO graphene provides reactive sites, allowing precise ASD even in the presence of polymer contamination. To emphasize the advantage of our AS-ALD/MLD approach, we present a comparison table (Table S4) that summarizes representative studies on conventional ALD, MLD, and AS-ALD for bulk, 1D and 2D materials. While selective ALD and MLD processes have been demonstrated individually, no prior work has been reported on AS-ALD/MLD on 2DM.

### Graphene/Eu-Organic Heterostructures

Figure 3a and 3b show the AFM and surface potential images of the graphene/Eu-BDC structures after ASD of an ∼7.1 nm Eu-BDC film. The three distinct squares correspond to TPO graphene regions irradiated with fs-laser doses of 40, 90, and 160 pJ^2^s. The height and work function (WF) profiles are shown in Figure 3c. The surface potential image was obtained using Kelvin probe force microscopy (KPFM) with an Au-coated AFM tip. The WF values were determined by calibrating the tip on an Au reference (5.3 eV) and converting the measured surface potential relative to this reference.

**Figure 3:**
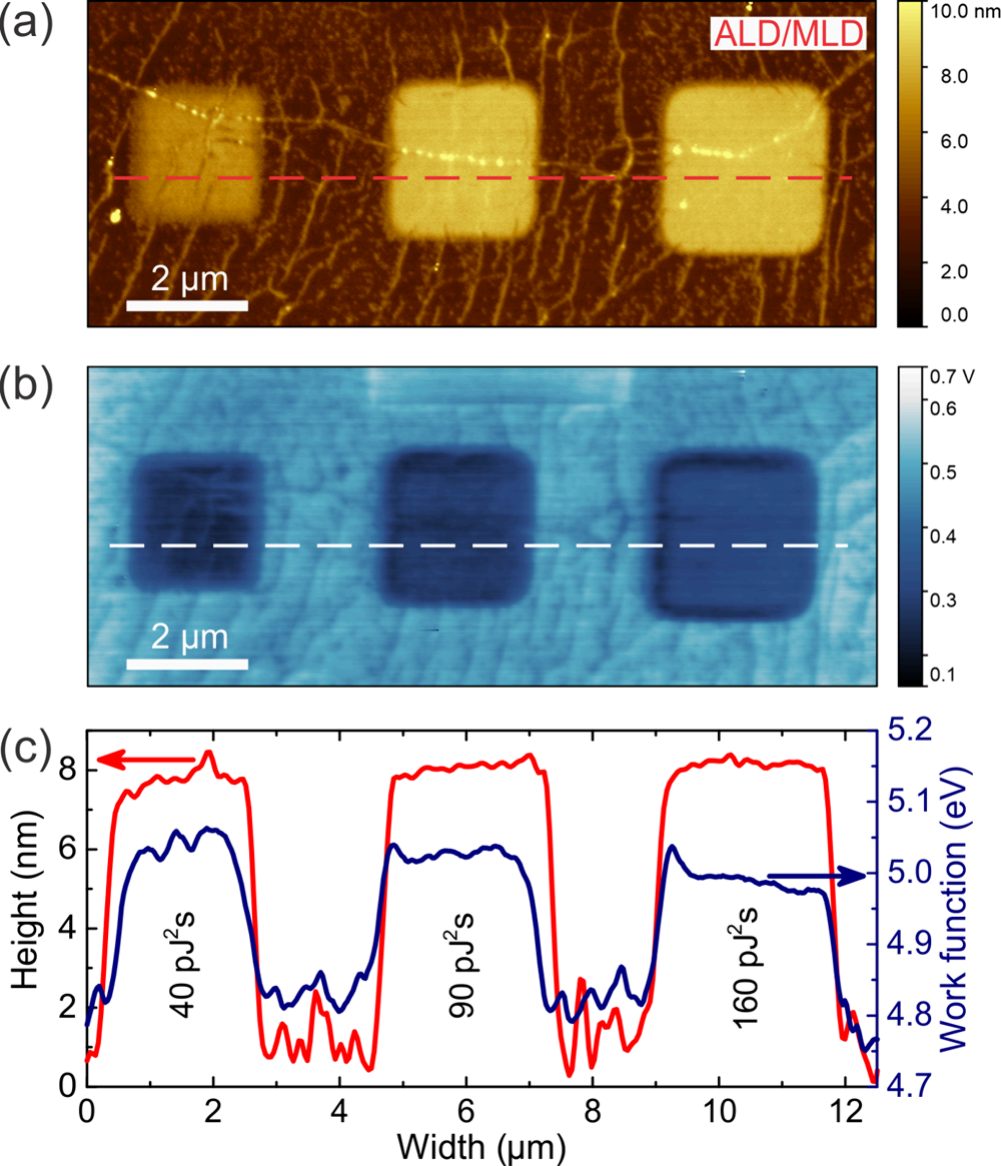
a) AFM image of PVAc graphene after 50 ALD/MLD cycles of Eu-BDC. b) Surface potential distribution of PVAc graphene after 50 ALD/MLD cycles of Eu-BDC measured via KPFM. c) Height and work function profiles measured from dashed lines in a) and b).

The WF of pristine graphene was measured at 4.75 ± 0.05 eV, while the TPO graphene areas exhibited higher WF values of 5.06 eV at 90 pJ^2^s and up to 5.27 eV for 625 pJ^2^s (Figure S11), indicating significant p-type doping due to the binding of oxygen-containing groups to the graphene surface. The deposition of 50 ALD/MLD cycles of Eu-BDC at 250 °C slightly reduced the WF by ∼35 meV in the 90 pJ^2^s area. For 160 pJ^2^s, it decreased further by ∼100 meV, reaching 4.96 eV. This reduction in WF after Eu-BDC deposition suggests n-type doping at higher fs-laser doses, driven by an interface dipole and ground-state charge transfer at the graphene/Eu-BDC interface.

After the ALD/MLD process, the G and 2D bands in the Raman spectrum of pristine graphene show a blueshift, indicating p-type doping due to the enhanced interaction between graphene and the underlying SiO_2_ substrate, increased by annealing at 250 °C during deposition in an inert atmosphere. In the TPO areas, the blueshift of the G and 2D bands diminishes as the fs-laser dose increases (Figures S3 and S12). At 160 pJ^2^s, the 2D band shows a 2 cm^–1^ redshift, demonstrating that the charge transfer mechanism is more pronounced at higher fs-laser doses. A comparison of the KPFM and Raman results shows that higher laser doses promote stronger interactions between the Eu-BDC film and graphene, leading to a decreased WF and a shift toward n-type doping.

To directly access how Eu-BDC deposition affects the charge carrier concentration in graphene, we fabricated graphene field-effect transistors (GFETs) and conducted TPO and ALD/MLD of 10 nm films on distinct channels. Figure 4 shows the output (I_DS_–V_DS_) and transfer (I_DS_–V_GS_) current–voltage (I–V) characteristics of GFETs before and after fs-laser TPO and ALD/MLD of a 10 nm Eu-BDC film. A linear I_DS_-V_DS_ behavior was observed at all the stages of graphene modification (Figure 4a). After TPO (225 pJ^2^s), a slight decrease in current was observed when oxygen-containing functional groups were introduced. A significant Dirac point (V_D_) shift for TPO graphene in the I_DS_-V_GS_ curve (Figure 4b), from 279 mV to 520 mV, and increased transconductance, is consistent with p-type doping induced by oxygen species. Following ALD/MLD, the TPO GFET showed an increase in drain current due to the detachment of functional groups and Eu-BDC deposition, whereas for the pristine GFET, it dropped, indicating increased contact resistance or channel contamination.

**Figure 4:**
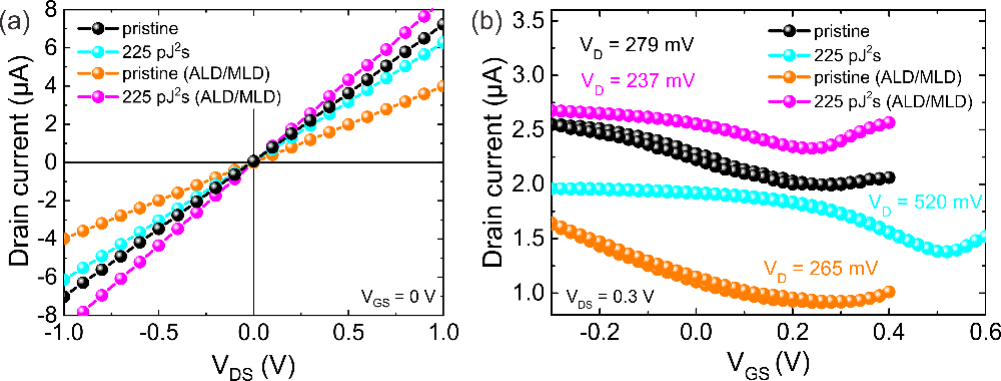
a) Output and b) transfer I–V curves of pristine and TPO graphene before and after ALD/MLD of a 10 nm Eu-BDC layer.

The transfer curves reveal a clear shift in the Dirac point from 279 mV to 237 mV after ALD/MLD of the Eu-organic thin film, corresponding to a 42 mV shift toward n-type doping (283 mV when referenced from the TPO graphene V_D_). Using the relation $\Delta n=\frac{C_{EDL}}{e}\left(V_{GS}-V_{D}\right)$ with an effective electric double-layer capacitance of 2 μF cm^–2^ for 0.01X PBS,^76^ we found that the pristine channel exhibits only a small carrier density change after ALD/MLD (−1.75 × 10^11^ cm^–2^), whereas the selectively coated TPO channel shows a significantly larger change (−5.24 × 10^11^ cm^–2^). This corresponds to an additional ∼3.5 × 10^11^ cm^–2^ carrier density modulation in the selectively grown Eu-BDC region, demonstrating that ASD produces a stronger and spatially localized electronic modulation.

This observation aligns well with the KPFM results, which indicate a decrease in WF after metal–organic film deposition and confirm electron transfer from the Eu-BDC layer to graphene. The annealing step during the ALD/MLD process restores graphene electronic properties to their pristine state. The Eu-BDC layer introduces a stable n-type doping, as observed by the V_D_ and WF shifts, indicating controlled charge transfer without permanent degradation of the graphene layer conductivity. In addition, no significant hysteresis was observed after ALD/MLD, indicating a clean graphene/Eu-BDC interface.

The ability to fine-tune the electronic properties of graphene/Eu-BDC heterostructures by controlling the fs-laser dose, ASD, and annealing conditions opens novel perspectives in 2DM electronic, optoelectronic, and sensor applications, where precise work function modulation and a clean interface are essential for optimizing device performance. Variations in ALD/MLD film thickness can also affect the built-in electric field generation in p–n junctions upon incident light illumination.^77^

### Luminescent Properties of Graphene/Eu-Organic Heterostructures

To investigate the potential applications of graphene/Eu-organic heterostructures in photonics and imaging, we studied the photoluminescence (PL) signal and its lifetime, as well as the effects of the laser irradiation dose on these properties. Micro-PL spectra collected from different areas of the sample after 100 ALD/MLD cycles of Eu-BDC upon 532 nm laser excitation (Figure 5a) revealed four characteristic PL peaks of Eu^3+^ located at ∼579 nm, ∼592 nm, ∼612 nm (the most intense and sharpest red emission line), and ∼652 nm for Eu-BDC on SiO_2_ and TPO graphene. For pristine graphene with minimal Eu-BDC deposition, only the peaks at ∼579 nm and ∼612 nm were visible due to the small amount of PL material.

**Figure 5:**
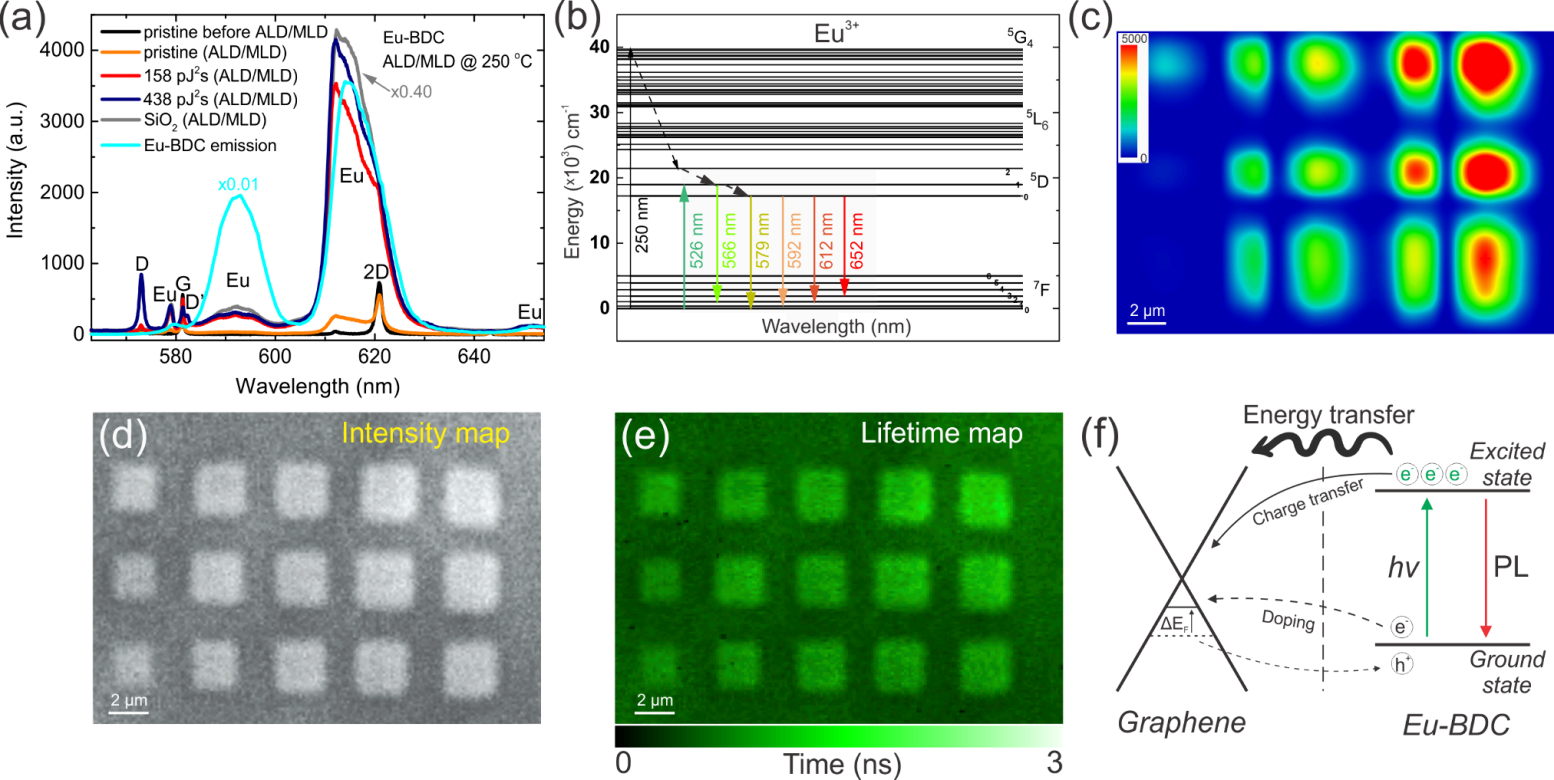
a) Micro-PL spectra of Eu-BDC films after 100 ALD/MLD cycles on SiO_2_, pristine graphene, and TPO graphene (158 and 438 pJ^2^s), excited by a 532 nm CW laser, along with the emission spectrum of a 100 nm Eu-BDC film on Si, excited by a 150 W Xe arc lamp at 250 nm. b) Energy-level scheme of the Eu^3+^ ion. c) Micro-PL map of the Eu-BDC film after 100 ALD/MLD cycles, measured at 612 nm and excited by a 532 nm CW laser. d) FLIM intensity map of the Eu-BDC film after 50 ALD/MLD cycles, excited by a 514 nm ps-laser. e) FLIM lifetime map of the Eu-BDC film after 50 ALD/MLD cycles, excited by a 514 nm ps-laser. f) Schematic representation of charge and energy transfer pathways at the graphene/Eu-BDC interface in the dark and under optical excitation.

We attribute the excitation at 532 nm to a pure magnetic dipole transition ^7^F_0_ → ^5^D_1_, which peaks at 526 nm (Figure 5b).^78,79^ The ^5^D_1_ state then relaxes nonradiatively to the level ^5^D_0_, which emits via the ^5^D_0_ → ^7^F_j_ (j = 0, 1, 2, 3) transitions. However, the emission may also occur via ^5^D_1_ → ^7^F_j_ transitions with a much shorter lifetime.^78^

A strong PL signal after ALD/MLD centered at ∼612 nm was detected on TPO graphene prepared with a relatively low irradiation dose of 90 pJ^2^s, and the signal intensity increased with higher TPO irradiation doses (Figure 5c). The PL intensity from the TPO areas was up to 20 times higher at a fs-laser dose of 158 pJ^2^s and up to 52 times higher at a 625 pJ^2^s dose compared to the PL signal from pristine graphene areas after 50 ALD/MLD cycles (Figure 1e). However, after 100 ALD/MLD cycles, the enhancement of PL intensity in the TPO areas decreased, and was ∼15 and ∼20 times stronger, respectively, due to the partial deposition of Eu-BDC on the pristine graphene areas (Figure 5a). Overall, this provides strong evidence of precise AS-ALD/MLD deposition of Eu-BDC films on TPO graphene, demonstrating the ability to localize luminescence through selective deposition.

We observed quenching of the PL signal on the TPO graphene surface compared to SiO_2_ (Figure 5a). The level of PL quenching in Eu-BDC films strongly depends on the film thickness, which is consistent with the Förster-type energy transfer (FRET) mechanism described for dipole emitters near graphene. The nonradiative energy transfer rate scales as r^–4^, yielding >99% efficiency at distances below 5 nm and vanishing above 20 nm.^80^ Our results demonstrate a clear dependence of the fluorescence quenching effect on the thickness of the Eu-BDC films deposited via ALD/MLD, as shown in Figure 5. The data support that near-field FRET dominates below ∼10 nm, while thicker films show partial recovery of luminescence as the emitter-graphene separation increases. Alternatively, the quenching effect could be due to electron transfer. After excitation of the Eu^3+^, an excited electron can transfer to the graphene conduction band, leading to fluorescence quenching.^81^

Fluorescence quenching directly affects the lifetime of Eu-BDC on graphene. We performed fluorescence lifetime imaging microscopy (FLIM) to investigate the fluorescence dynamics of ALD/MLD Eu-BDC thin films on pristine and TPO graphene. While the overall fluorescence intensity (Figure 5d) was low upon 514 nm pulsed laser excitation, the lifetime of Eu-BDC on TPO areas was significantly longer than on pristine graphene (Figure 5e). The measured lifetime of ∼2–3 ns is much shorter than the reported lifetimes for Eu^3+^ films (∼10 μs for transitions originating from the ^5^D_1_ state^78^ and ∼1.5 ms for transitions from the ^5^D_0_ state).

In a few samples, we also detected a peak at ∼566 nm upon CW laser illumination (Figure S13). These emissions indicate contributions from the ^5^D_1_→^7^F_2_ (∼566 nm) and ^5^D_1_ → ^7^F_3_ (∼579 nm) transitions to the PL spectrum of the ALD/MLD films when direct excitation of the ^5^D_1_ level occurs.^78,82^ Interestingly, the green emission at ∼566 nm was observed only on pristine and TPO graphene and was absent on Si and SiO_2_. Note that 532 nm excites the ^5^D_1_ level, and we do not consider transitions from higher energy states.

When excited by a 532 nm CW laser, the intensities of the PL peaks at ∼566 nm and ∼579 nm, relative to the peak at ∼612 nm, are significantly higher for Eu-BDC on graphene compared to the 100 nm Eu-BDC film on Si, which was excited at 250 nm (Figures 5a and S14). Therefore, the emission from the ^5^D_1_ level is enhanced compared to the emission from the ^5^D_0_ level for Eu-BDC films on pristine and TPO graphene. The ^7^F_0_ → ^5^D_1_ pure magnetic dipole transition, excited by a 532 nm laser, populates the ^5^D_1_ level. One possible enhancement mechanism could be the suppression of nonradiative relaxation to the ^5^D_0_ level, but it is difficult to identify any graphene-induced process responsible for this. Another possibility is an enhanced radiative rate from the ^5^D_1_ state to ^7^F_j_, however, we are not aware of any graphene-induced mechanism behind this. A third possibility is a thermally activated enhancement of the ^5^D_1_ state population, potentially caused by laser-induced heating of graphene. We tested this hypothesis by measuring the PL spectra at different excitation powers (Figure S15a), but the results did not show enhanced emission with increased power. Additionally, the PL intensity of the peak at ∼612 nm after 532 nm CW laser excitation demonstrates a linear response (Figure S15b), showing that no two-photon absorption processes are involved in signal generation. While the mechanism behind this enhancement remains unclear, it may arise from local structural differences or altered molecular orientation of Eu-BDC on the oxidized graphene surface.

The enhanced PL intensity and shorter fluorescence lifetimes observed for Eu-BDC on TPO graphene suggest efficient energy and charge transfer between the molecular film and graphene. The FRET dominates the quenching behavior. In contrast, a minor contribution from charge transfer to graphene is supported by the reduced WF and n-type doping after ALD/MLD. On TPO graphene, oxygen groups act as spacers, altering electronic coupling and partially suppressing quenching. These mechanisms are illustrated in Figure 5f, which summarizes electron and energy transfer pathways between Eu-BDC and graphene in the dark and under optical excitation.

We also demonstrated that the fluorescence dynamics of uncoated graphene was not affected by the TPO process itself by performing FLIM measurements after UDLW (Figure S16a,b). No visible signal was detected when the surface was irradiated with the same fs-laser doses and measured under the same conditions as in Figure 5e. However, other mechanisms, in addition to energy and electron transfer to graphene, may be involved, as short lifetimes were also observed for Eu-BDC thin films on SiO_2_ (Figure S16c,d). Notably, Eu-BDC demonstrates very weak absorption at the laser wavelengths used (514 and 532 nm).^60^ Therefore, the PL is expected to be much stronger upon resonant excitation in the UV range. The observed properties of Eu-BDC/graphene heterostructures could have potential applications in bioimaging, where tunable photoluminescence may enable the precise tracking of biological processes in real-time,^83^ and in solar cells, where enhanced electron transfer could improve charge separation efficiency.^3^

## Conclusions

In summary, we successfully demonstrated area-selective ALD/MLD of Eu-organic thin films on single-layer graphene using fs-laser TPO to locally activate predefined regions. The method achieved high homogeneity and over 90% selectivity for Eu-organic films up to 11 nm, overcoming the chemical inertness of graphene and establishing a resist-free, scalable, and controllable approach. The transfer polymer strongly affected the selectivity, with PVAc minimizing residues and enabling better control over the deposition compared to PMMA. The resulting graphene/Eu-organic heterostructures exhibited strong PL emission upon 532 nm excitation, a unique result as Eu^3+^ ions typically require UV excitation. Efficient energy and charge transfer were confirmed by Raman, I–V characteristics, work function, and fluorescence lifetime measurements. At moderate TPO doses (>160 pJ^2^s), reversible n-type doping was observed after ALD/MLD, while postannealing restored graphene close to its initial electronic state, confirming controllable surface modification.

The AS-ALD/MLD method presented here is not limited to Eu-organic thin films on graphene. The TPO activation strategy provides a general framework for ASD on a wide range of 2DM and dielectric surfaces. Beyond graphene, we applied this approach to MoS_2_ and WS_2_, where oxygen-containing functional groups (e.g., MoO_3_, WO_3_) or point-like defects formed under fs-laser irradiation acted as active nucleation sites for Eu-BDC growth.

While the polymer transfer method significantly influences selectivity, as demonstrated for PVAc and PMMA, it is possible to reach a selectivity of over 90%. Our future studies aim to explore alternative transfer techniques, such as polymer-free dry transfer and TPO and ALD/MLD on freshly grown 2DM. On the other hand, the control over generated defects via TPO is another valuable parameter, which can easily affect graphene electrical properties. Higher fs-laser doses create more oxygen functional groups, resulting in more homogeneous films, but may disrupt the underlying material. Laser power and exposure duration are crucial parameters for balancing selectivity and material integrity. The choice of ALD/MLD precursors can be optimized for a specific material by selecting ligand chemistries that enhance deposition selectivity while minimizing nonspecific adsorption on pristine areas. Combining TPO with near-field techniques may enable highly localized patterning at the nanoscale, enhancing resolution below 10 nm, which is compatible with the current CMOS technology.

The demonstrated approach provides a path forward for integrating 2DM-based heterostructures into optoelectronic, photonic, and energy-harvesting devices. Submicron precision achieved in this work expands the design possibilities for multifunctional devices on a single chip. Further optimization of TPO and ALD/MLD parameters may enable large-scale fabrication with uniform growth, facilitating the development of scalable, high-performance electronic and photonic systems.

## Methods

### Graphene Samples Fabrication

Graphene was synthesized on 500 nm Cu (111) films, which were evaporated onto single-crystal sapphire (0001) substrates, with a growth time of 25 min at 1050 °C. The graphene films were transferred onto Si substrates with a 300 nm SiO_2_ layer and a Pd/Ti grid using a PMMA- or PVAc-assisted transfer method. Eighteen samples were divided into three groups: (1) not annealed, (2) annealed at 350 °C in Ar/H_2_ atmosphere for 2 h, and (3) additionally annealed at 280 °C in O_2_ atmosphere for 2 h to remove polymer residues from the graphene surface (Table S2). GFETs were fabricated using a standard electron-beam lithography (EBL) process.^84^ The metal contacts were patterned on the chip using EBL, followed by thermal evaporation of a Ti/Pd layer (10/100 nm). Unwanted graphene outside the active regions was removed by oxygen plasma etching to define the GFET channel areas of 40 × 60 μm^2^.

TPO of graphene, MoS_2_, and WS_2_ was performed with a 515 nm fs-laser (Pharos-10, Light Conversion Ltd., 600 kHz repetition rate, 250 fs pulse duration) focused through a 100x objective with an NA of 0.8 in an ambient atmosphere with a relative humidity of 35%.^43^ Graphene was irradiated using laser pulse energies ranging from 10 to 30 pJ (corresponding to 6–18 μW average power) and exposure times from 0.4 to 1 s per spot, producing patterns with different oxidation levels. Using 515 nm irradiation enables two-photon absorption equivalent to deep-UV photon energies, promoting surface-localized oxidative functionalization of adsorbed oxygen and water molecules. Short femtosecond pulses allow operation at low average power, which eliminates photothermal effects while reaching effective two-photon excitation due to high peak intensity. The low average power also results in negligible heating of the sample during the laser irradiation. The laser dose, defined as the square of pulse energy multiplied by irradiation time, was used to compare the oxidized squares. This definition was chosen because it is proportional to two-photon absorption and irradiation time. Matrices of approximately 2 × 2 μm^2^ squares were patterned via step-by-step irradiation, with a 0.1 μm separation between consecutive laser spots. GFET channels were irradiated using a 4x objective with an NA of 0.1 and 0.5 μm steps.

### ALD/MLD of Eu-BDC

Eu-BDC thin films were deposited by ALD/MLD technique utilizing Eu­(thd)_3_ and 1,4-benzene dicarboxylic acid (BDC) as precursors, where thd = 2,2,6,6-te-tramethyl-3,5-heptanedione. Eu­(thd)_3_ was synthesized in-house, as first described by Eisentraut and Sievers.^85^ BDC (99%) was obtained commercially from Tokyo Chemical Industry Co., Ltd. and used without further treatment. The powder precursors were stored inside the reactors in open glass crucibles and maintained at 140 and 185 °C for Eu­(thd)_3_ and BDC, respectively, during the deposition. All depositions were carried out in a commercial flow-type hot-wall ALD reactor (F-120, ASM Microchemistry Ltd.). During the deposition process, the reactor pressure was maintained between 2 and 4 mbar. In-house generated nitrogen was used as both the carrier and purging gas. The precursor pulse and purge lengths were adopted from previous work.^60^ The pulse length for Eu­(thd)_3_ was 4 s, followed by a 6-s purge, while the pulse length for BDC was 7 s, followed by a 15-s N_2_ purge. The samples were kept at either 200 or 250 °C during deposition.

### Graphene/Eu-Organic Heterostructures Characterization

The thicknesses of Eu-BDC films grown on silicon witness samples were determined with X-ray reflectivity (PANalytical X’Pert diffractometer, Cu Kα source), and the data were fitted using X’Pert Reflectivity software by PANalytical. Si samples were used to standardize the growth per cycle. On Si substrates, Eu-BDC films demonstrated a growth rate of 3.0 Å/cycle at 200 °C and 2.5 Å/Cycle at 250 °C. As expected, the GPC in ALD/MLD processes decreased with increasing deposition temperature.^71^ The emission spectrum of a 100 nm Eu-BDC film was measured via a LOT QD MSH-300 monochromator and a CW 150 W Xe arc lamp at 250 nm.

AFM imaging was carried out on a Bruker Dimension Icon atomic force microscope using PeakForce Tapping mode and ScanAsyst-Air probes with the peak force set to 2.0 nN. Kelvin probe force microscopy was performed at room temperature using Au-coated NSG03 cantilevers (TipsNano). Raman mapping and spectra acquisition were conducted using a DXR Raman (Thermo Scientific) microscope with a 50x objective, 1 mW laser power, and an excitation wavelength of 532 nm. Helium ion microscopy images were obtained by a Zeiss Orion Nanofab microscope with a beam current of 0.12 pA, 10 μs dwell time, and 30 μm aperture.

Infrared scanning near-field optical microscopy experiments were performed on a neaSNOM device (attocube systems AG) equipped with a broadband laser continuum, achieving a spatial resolution below 30 nm through near-field interaction between the sample and an Au-coated AFM tip.^48,49^ Initially, an AFM topographic image was taken from the area of interest, followed by nano-FTIR spectra taken from specific spots, including background spectra from outside the region. The integration time per pixel was 70 ms with a spectral resolution of 15 cm^–1^. Each spectrum was divided by the corresponding background spectrum.

Electrical characterization of the GFETs was carried out in a liquid-gated configuration using a 0.01X PBS solution as the electrolyte. An Ag/AgCl reference electrode served as the gate electrode, while the V_DS_ was kept constant at 0.3 V. The V_GS_ was swept from −0.3 V to +0.6 V and back to obtain the transfer characteristics. The device area was immersed in the electrolyte during measurements, and all measurements were performed at room temperature. More details of the in-house-built measurement setup used are described elsewhere.^84^

FLIM measurements were conducted using a Leica SP8 X Falcon confocal microscope with a pulsed white light laser operated at an 80 MHz repetition rate. The excitation wavelength was 514 nm with an average power of 15 μW at the sample, and emission was collected in the 575–750 nm range.

## Supplementary Material



## Notes

Chemical composition of TPO graphene, fabrication parameters with detailed AFM and Raman analysis after TPO and ALD/MLD, FTIR, Nano-FTIR, work function, PL, and FLIM data for graphene; AFM and Raman data for MoS_2_ and WS_2_ after TPO and ALD/MLD; Comparison table of recent studies on conventional ALD, MLD, and AS-ALD for bulk, 1D and 2D materials (PDF)

## Notes

The preprint version of this work is Emelianov, A. V.; Mentel, K. K.; Ghazy, A.; Pekkanen, J.; Wang, Y.-H.; Johansson, A.; Karppinen, M.; Pettersson, M. Area-Selective Atomic/Molecular Layer Deposition of Europium-Organic Thin Films on Graphene and Other 2D Materials for Photoluminescent Heterostructures. 2025, chemrxiv-2024–81qv0-v2. ChemRxiv. 10.26434/chemrxiv-2024-81qv0-v2 (accessed Feb. 28, 2026).
