# Supplementary material for: Area-Selective Atomic/Molecular Layer Deposition of Europium-Organic Thin Films on Graphene and Other 2D Materials for Photoluminescent Heterostructures
Source: ACS Nano. 2026 Mar 17;20(12):10062–73. doi: 10.1021/acsnano.5c22728 (PMC13045344; doi:10.1021/acsnano.5c22728)
Supplement: Supplementary file 1 [file nn5c22728_si_001.pdf]

Supporting Information for

Area-Selective Atomic/Molecular Layer Deposition of Europium-Organic Thin Films on Graphene and Other 2D Materials for Photoluminescent Heterostructures

Aleksei V. Emelianov,[1] Kamila K. Mentel,[1] Amr Ghazy,[2] Joona Pekkanen,[2] Yu-Han Wang,[1] Andreas Johansson,[1,3] Maarit Karppinen,[2,*] Mika Pettersson[1,*]

[1]Nanoscience Center, Department of Chemistry University of Jyväskylä, FI-40014 Jyväskylä, Finland

[2]Department of Chemistry and Materials Science, School of Chemical Engineering, Aalto University, FI-00076 Aalto, Espoo, Finland

[3]Nanoscience Center, Department of Physics University of Jyväskylä, FI-40014 Jyväskylä, Finland

*e-mail: maarit.karppinen@aalto.fi, mika.j.pettersson@jyu.fi

Table of Contents:

Chemical Composition of TPO Graphene 2

Fabrication Parameters and AFM Data 2

Raman Data for Pristine Graphene and Graphene after TPO and ALD/MLD 13

FTIR and nano-FTIR spectra 22

Defect Concentration in Graphene after TPO and ALD/MLD Processes 23

Additional Data on Eu-BDC Film Thickness and Selectivity on Graphene 24

Graphene Height before and after TPO 24

TPO and ALD/MLD of Eu-organic Films on MoS_2 and WS_2 25

Table S4. Comparison of ALD, MLD, and AS-ALD Approaches on Bulk, 1D and 2D Materials. 28

The Work Function and Raman G and 2D Band Shifts after TPO and ALD/MLD 31

Micro-PL Spectra for Graphene after ALD/MLD of Eu-BDC Films 32

Emission Spectra of Eu-BDC Thick Film on Si 32

PL Spectra at Different Excitation Powers 33

Additional FLIM Data 33

References 34

Chemical Composition of TPO Graphene

Based on our XPS analysis of TPO graphene [S1], we can access this information by analyzing the I_D/I_G and $I_D/I_{D'}$ ratios from the Raman spectra of irradiated areas and comparing them with known data. At low and moderate laser doses, we introduce C-O-C and -OH groups, while at high doses, the amount of -COOH and -C=O groups increases and causes the formation of vacancies and holes in the graphene layer (Table S1), when the I_D/I_G ratio starts to decrease after reaching its maximum.

Table S1. Chemical composition of pristine and TPO graphene for Sample 8. Data were calculated from Ref. [S1].

Dose/concentration	C=C, %	C-C, %	C=OH, %	C-O-C, %	COOH, %
Pristine	67	28	2.5	2	0.5
90 pJ^2s	42.7	51	5	1	0.3
160 pJ^2s	43	38	15	3	1
280 pJ^2s	35	42.3	16	6	0.7
400 pJ^2s	14	28	38	18	2
630 pJ^2s	10	17	42	27	4
900 pJ^2s	8	24	40	25	3

Fabrication Parameters and AFM Data

Table S2. Fabrication parameters, ALD/MLD thickness from AFM, and calculated selectivity.

Sample	Transfer polymer	Pre-annealing*	Fs-laser dose range	ALD/MLD temperature	ALD/MLD thickness on SiO_2	ALD/MLD thickness on TPO graphene	ALD/MLD thickness on pristine graphene	Selectivity
1	PMMA	Ar/H_2	90-900 pJ^2s	200ºC	4.7 nm (25 cyc)	3.0 nm	0.34 nm	0.81
2	PMMA	Ar/H_2	90-900 pJ^2s	200ºC	10.9 nm (50 cyc)	7.1 nm	0.90 nm	0.77
3	PMMA	Ar/H_2	90-900 pJ^2s	200ºC	15.4 nm (75 cyc)	8.7 nm	3.80 nm	0.44
4	PMMA	Ar/H_2	90-900 pJ^2s	200ºC	20.6 nm (100 cyc)	15.45 nm	7.50 nm	0.34
5	PMMA	-	90-900 pJ^2s	200ºC	10.2 nm (50 cyc)	7.8 nm	2.07 nm	0.58
6	PMMA	-	90-900 pJ^2s	200ºC	20.2 nm (100 cyc)	12.1 nm	4.22 nm	0.48
7	PMMA	Ar/H_2	40-900 pJ^2s	250ºC	4.9 nm (25 cyc)	4.7 nm	0.44 nm	0.83

8	PMMA	Ar/H_2	40-900 pJ^2s	250ºC	9.7 nm (50 cyc)	6.6 nm	0.71 nm	0.81
9	PMMA	Ar/H_2	40-900 pJ^2s	250ºC	18.2 nm (100 cyc)	12.2 nm	1.37 nm	0.80
10	PMMA	Ar/H_2 + O_2	90-900 pJ^2s	250ºC	8.2 nm (50 cyc)	6.8 nm	1.90 nm	0.56
11	PMMA	Ar/H_2 + O_2	90-900 pJ^2s	250ºC	9.1 nm (50 cyc)	7.2 nm	1.57 nm	0.64
12	PMMA	Ar/H_2 + O_2	40-900 pJ^2s	250ºC	17.9 nm (100 cyc)	12.0 nm	9.5 nm	0.12
13	PMMA	-	90-900 pJ^2s	250ºC	9.8 nm (50 cyc)	9.0 nm	5.67 nm	0.32
14	PVAc	Ar/H_2	90-900 pJ^2s	250ºC	4.8 nm (25 cyc)	3.9 nm	0.68 nm	0.71
15	PVAc	Ar/H_2	40-900 pJ^2s	250ºC	9.5 nm (50 cyc)	7.3 nm	1.26 nm	0.70
16	PVAc	-	40-900 pJ^2s	250ºC	9.5 nm (50 cyc)	7.1 nm	0.19 nm	0.95
17	PVAc	-	90-900 pJ^2s	250ºC	14.2 nm (75 cyc)	10.8 nm	0.53 nm	0.91
18	PVAc	-	40-900 pJ^2s	250ºC	18.6 nm (100 cyc)	12.3 nm	1.18 nm	0.83

*In Ar/H_2 at 350ºC and in O_2 at 280ºC

Sample 1:

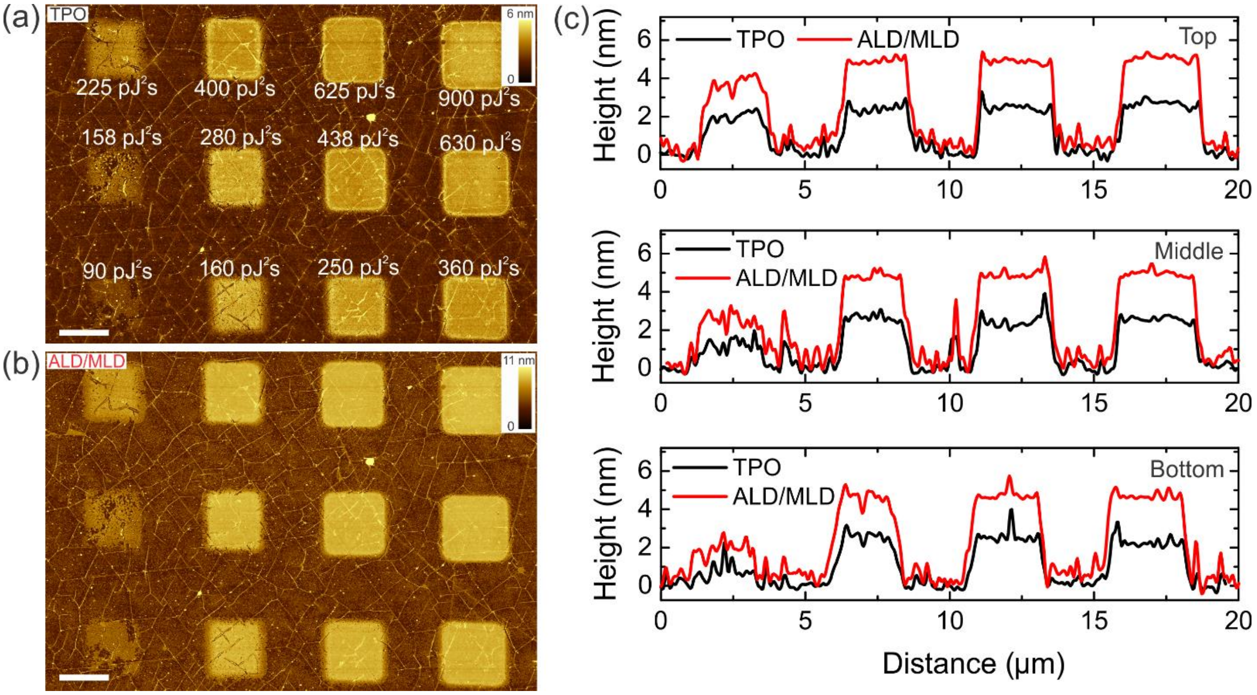

Sample 2:

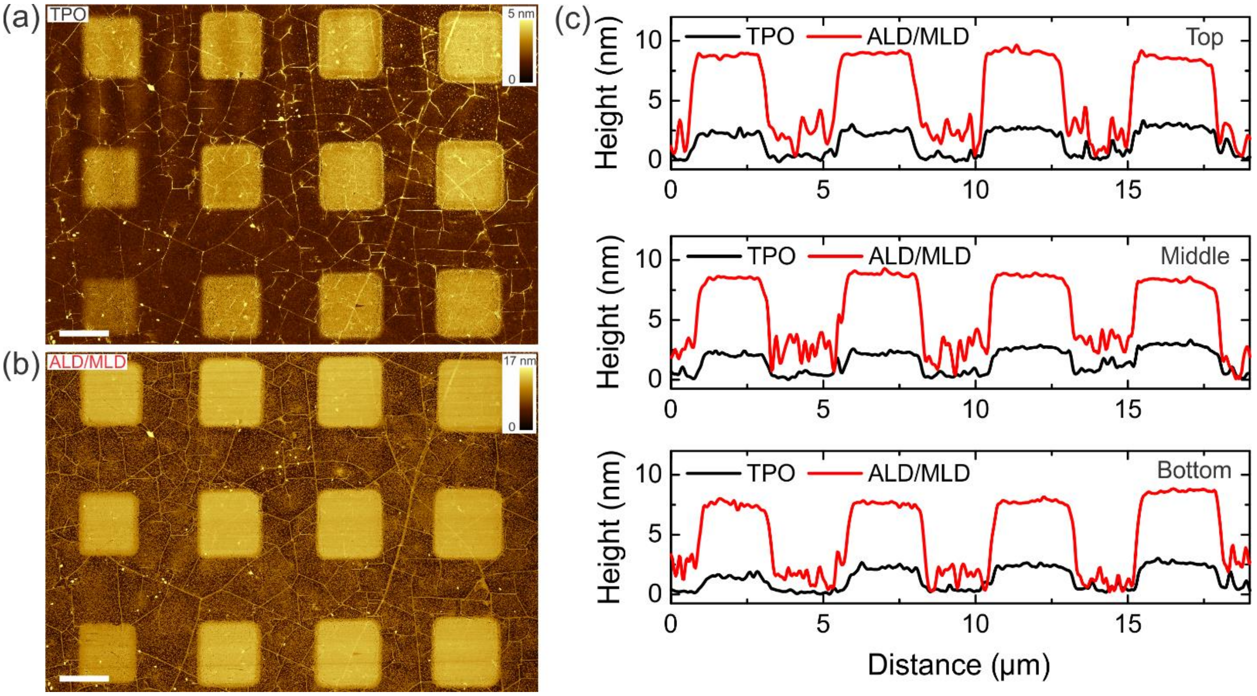

Sample 3:

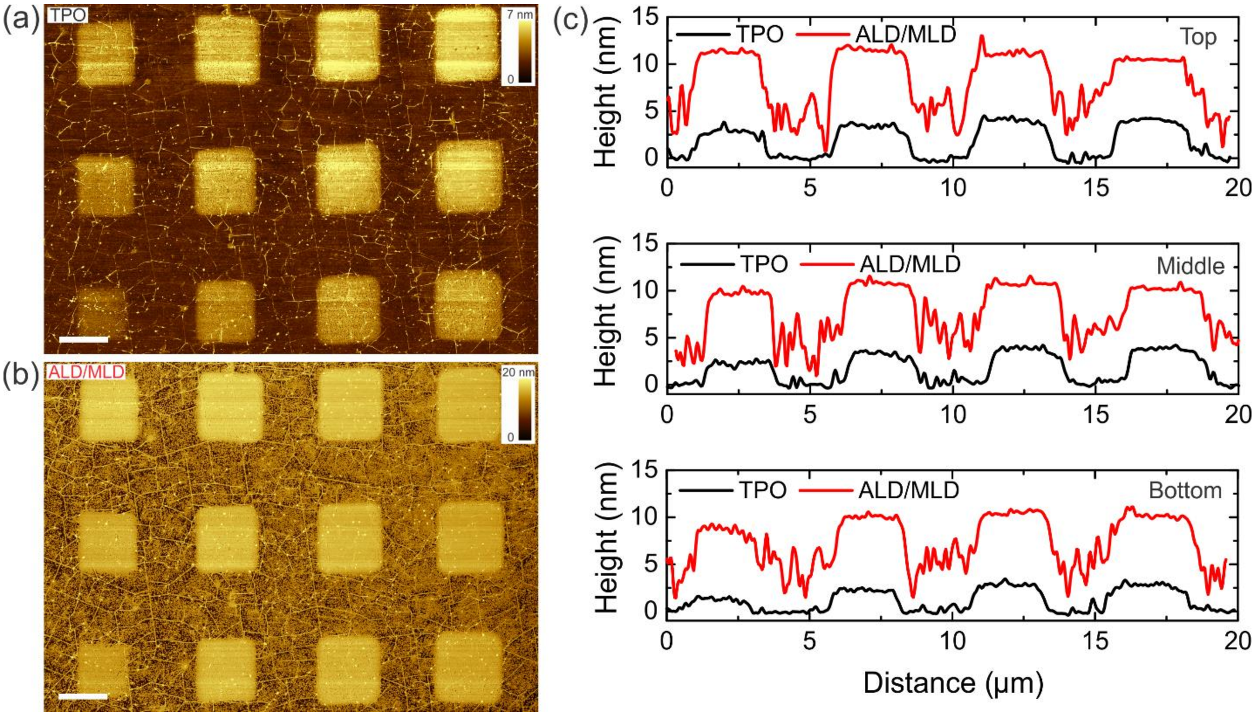

Sample 4:

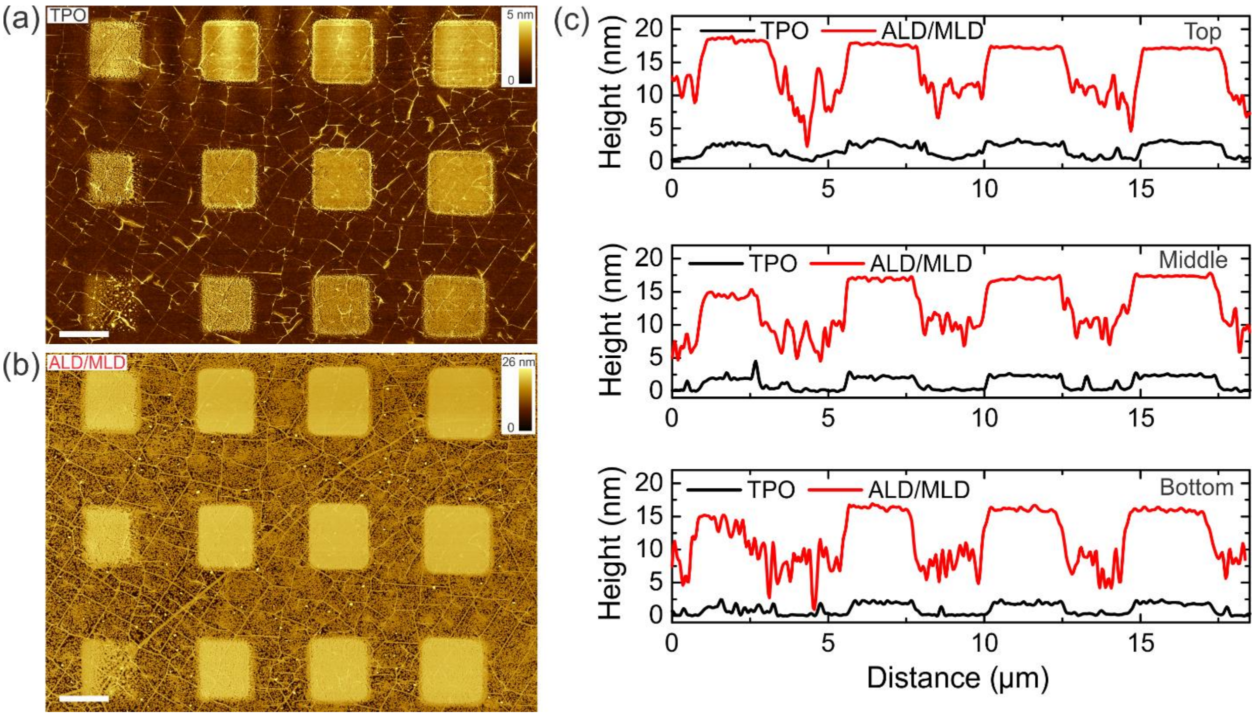

Sample 5:

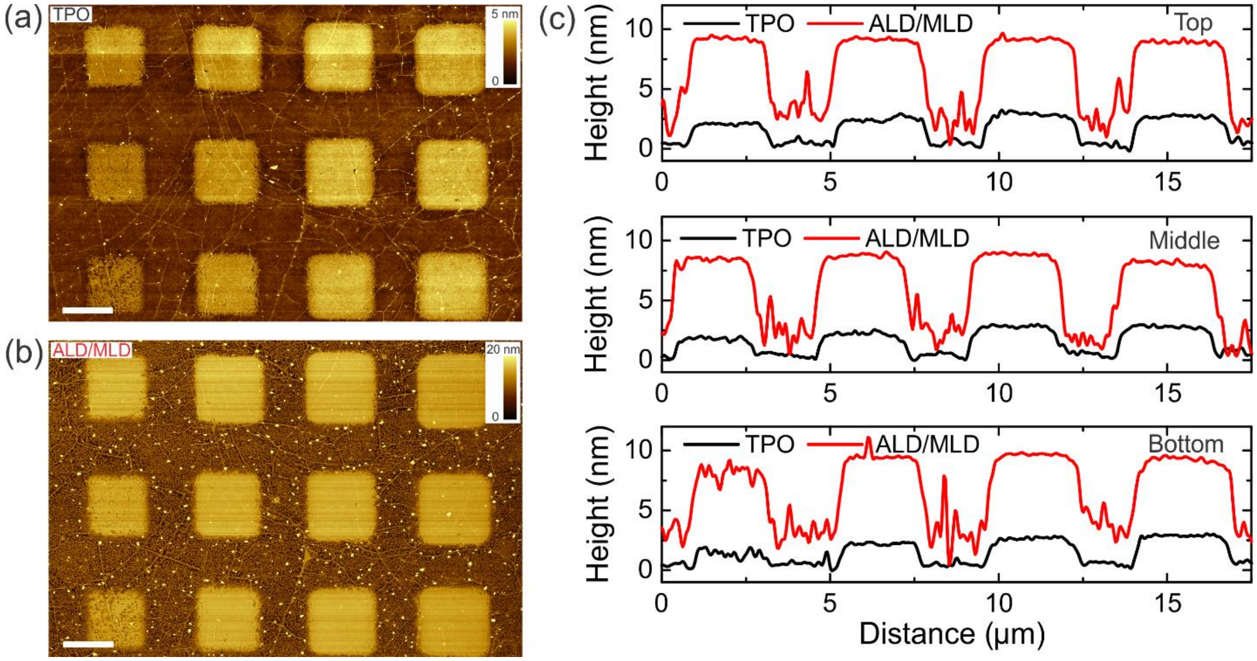

Sample 6:

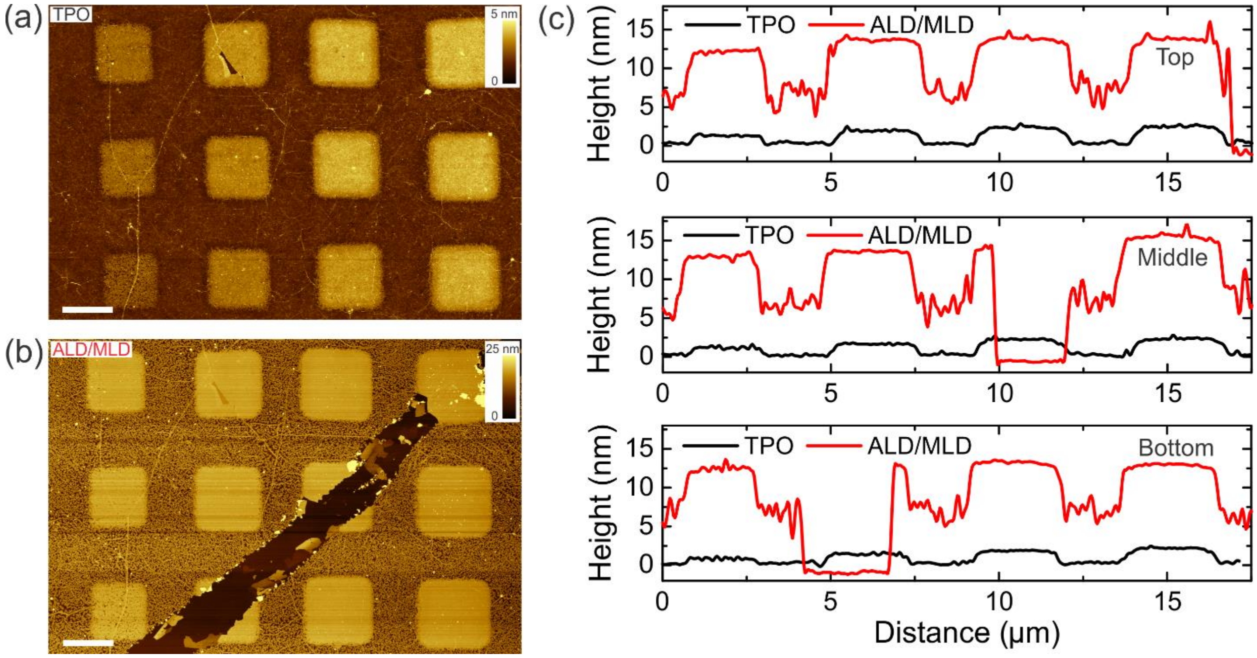

Sample 7:

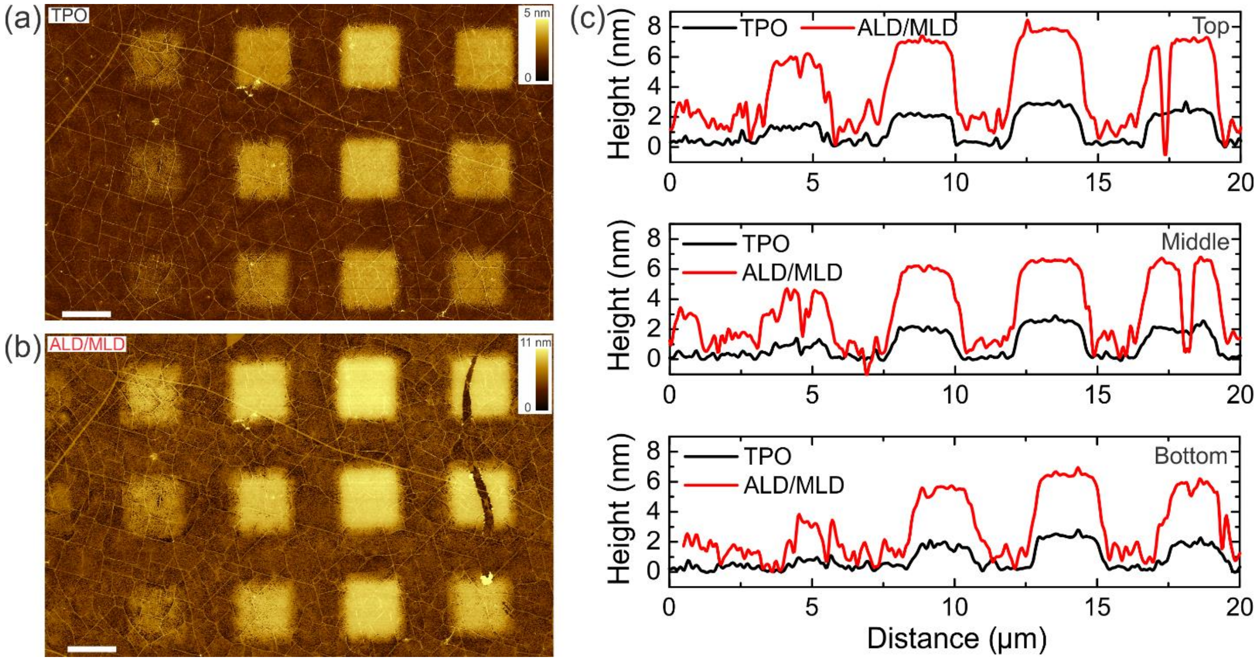

Sample 8:

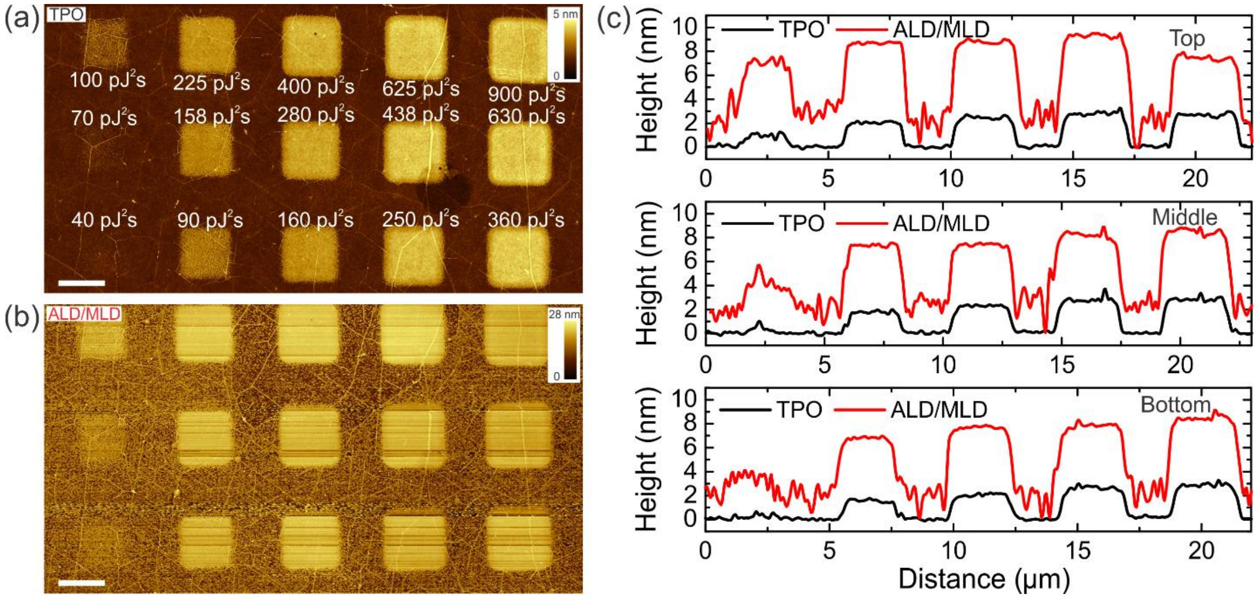

Sample 9:

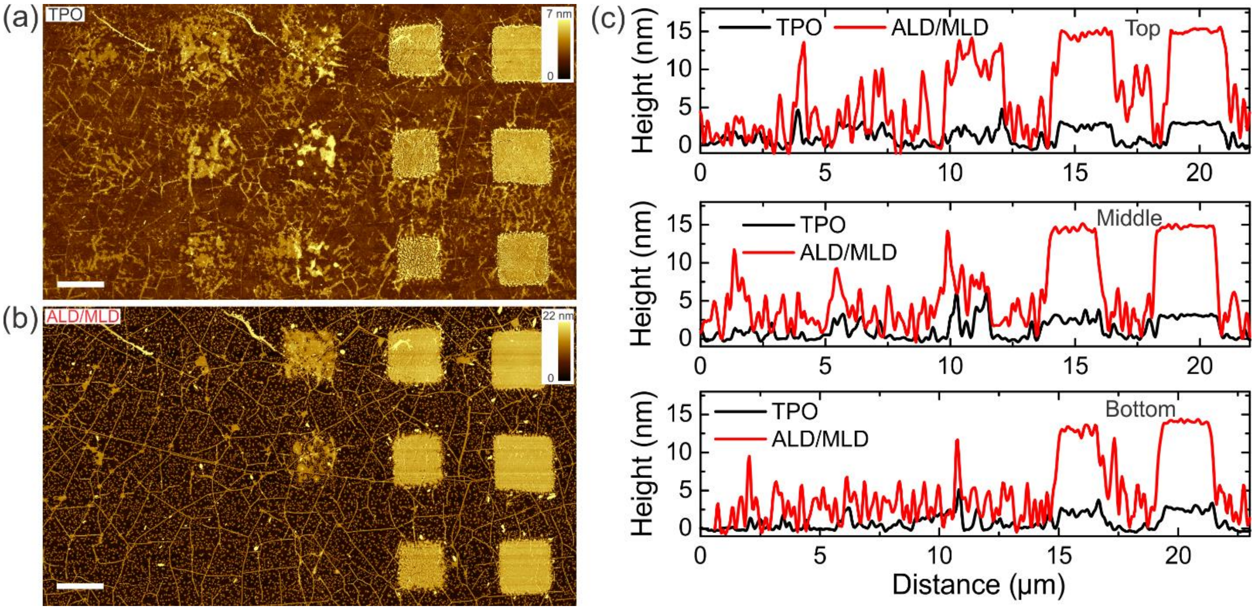

Sample 10:

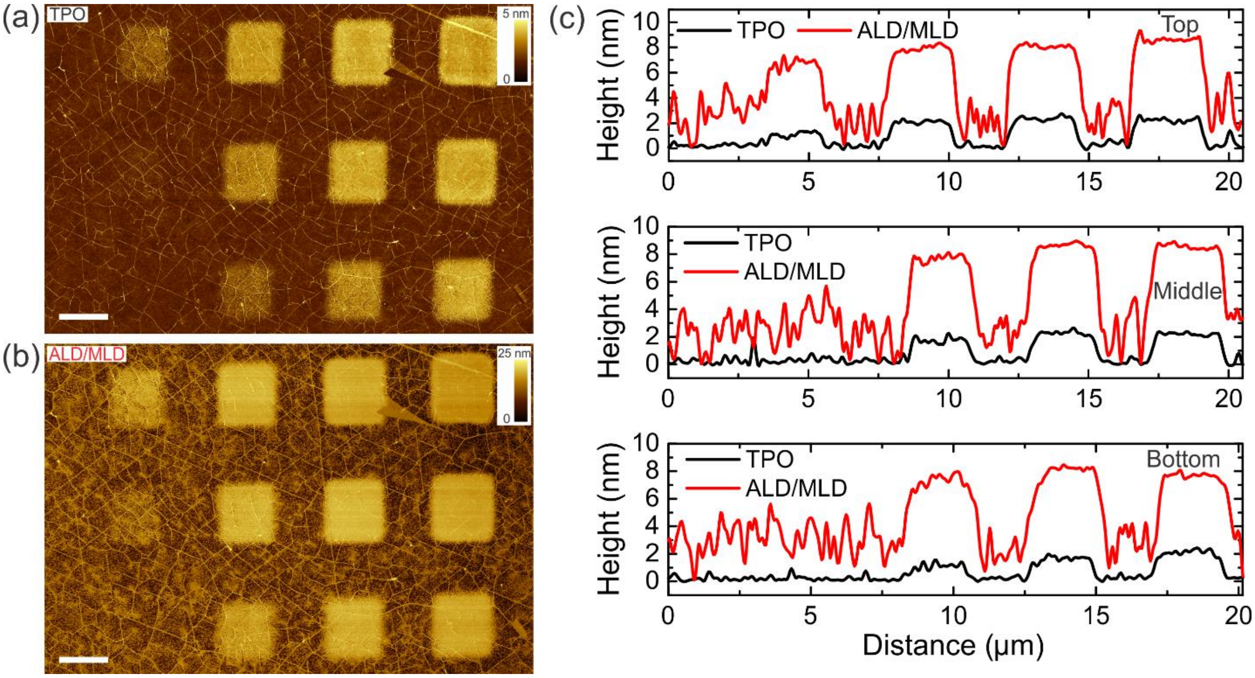

Sample 11:

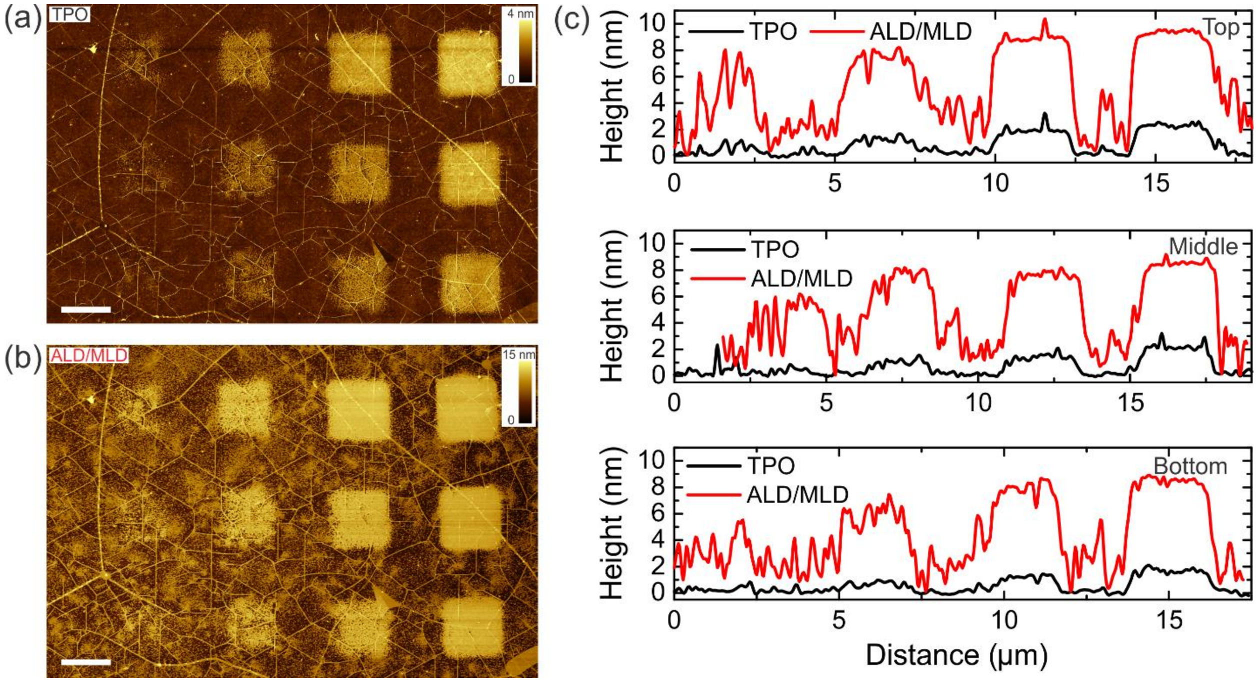

Sample 12:

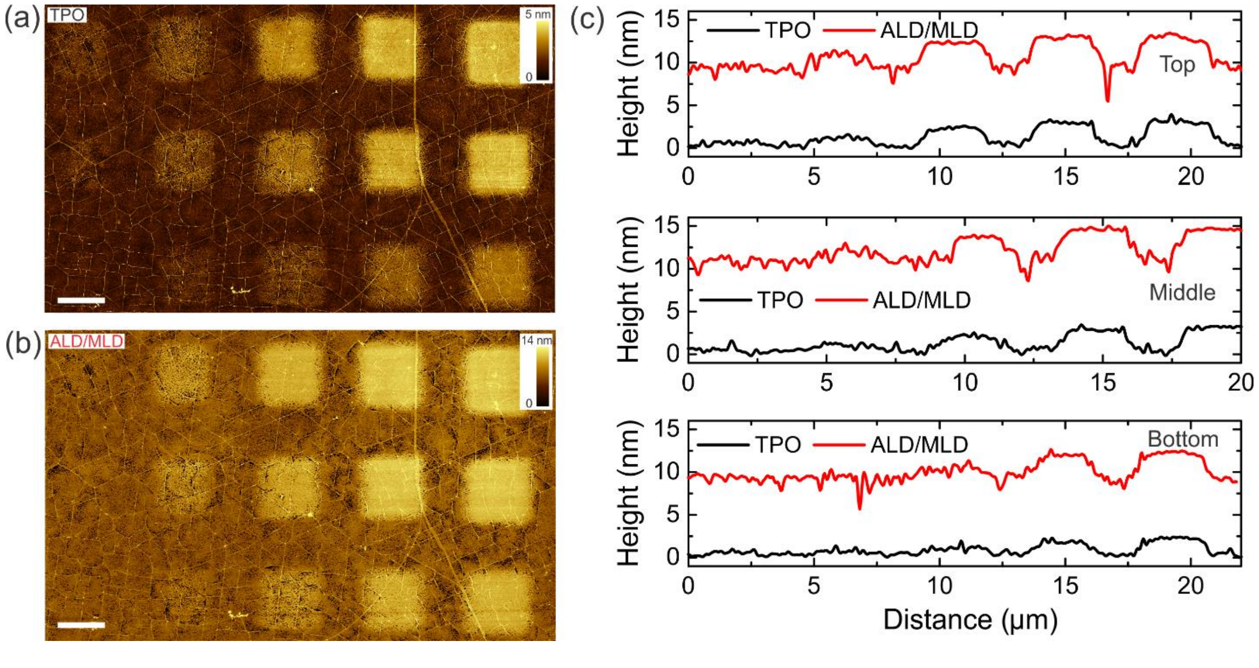

Sample 13:

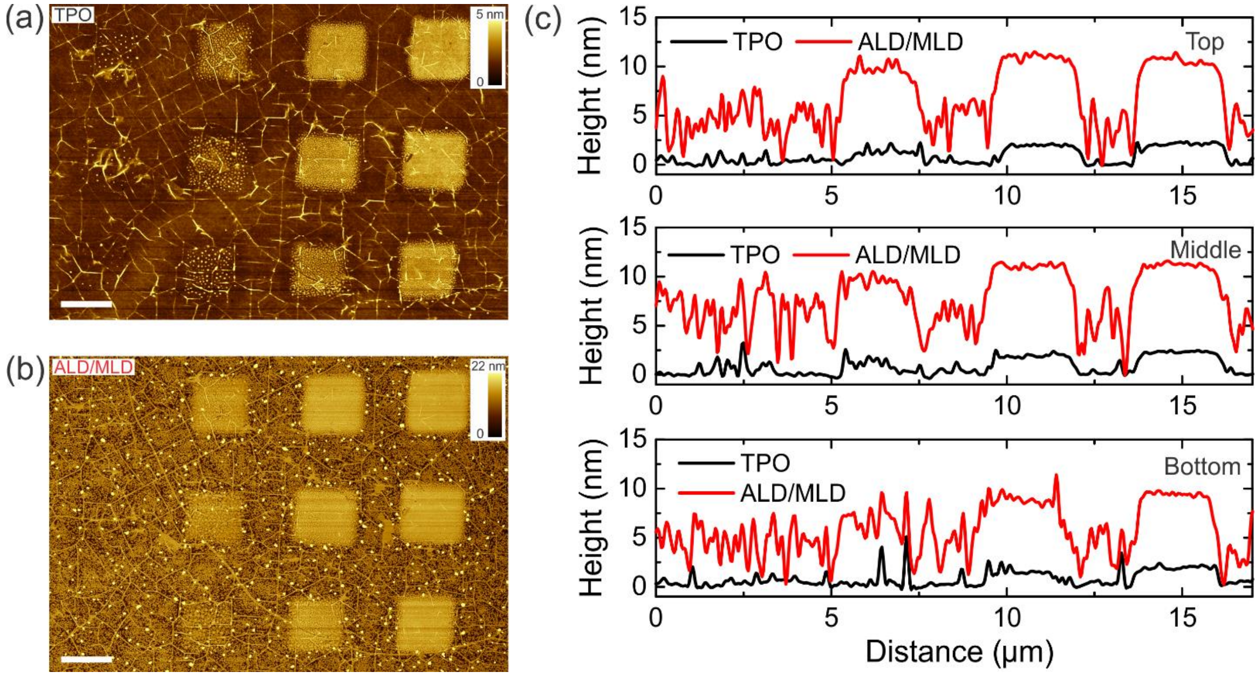

Sample 14:

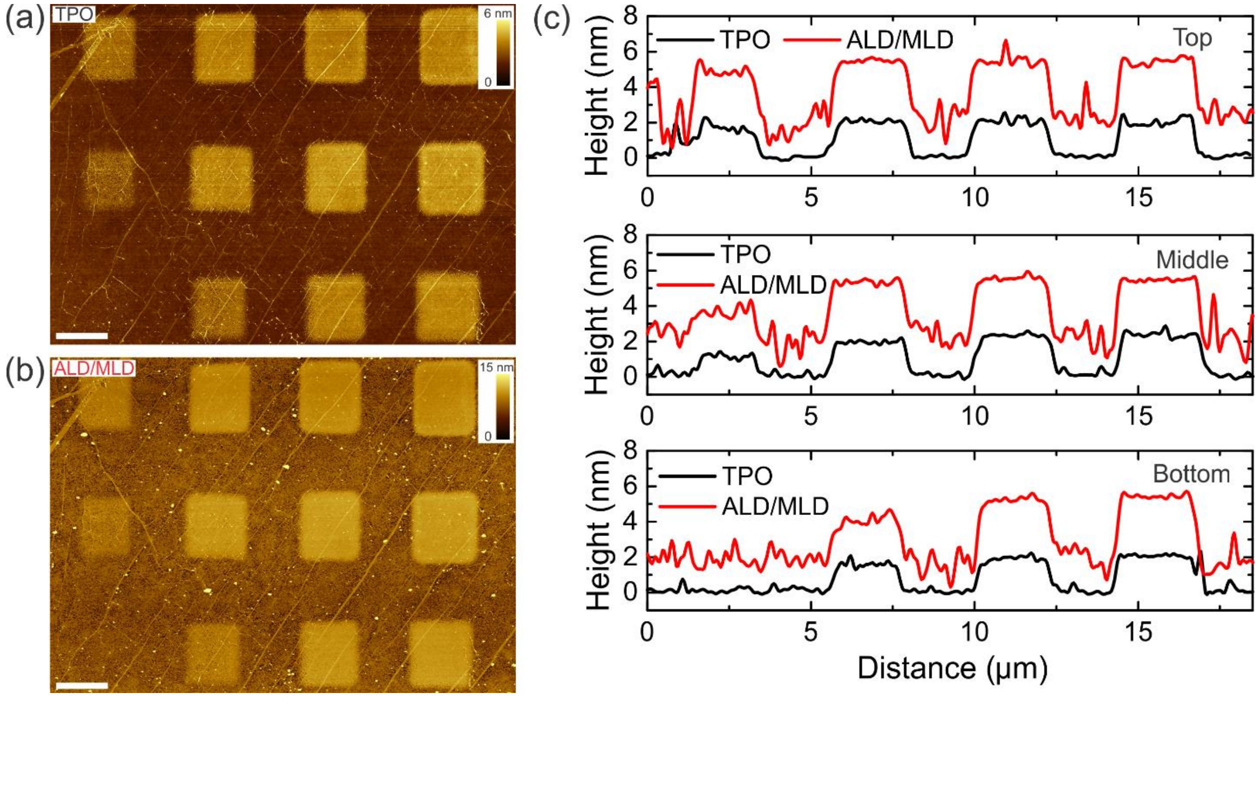

Sample 15:

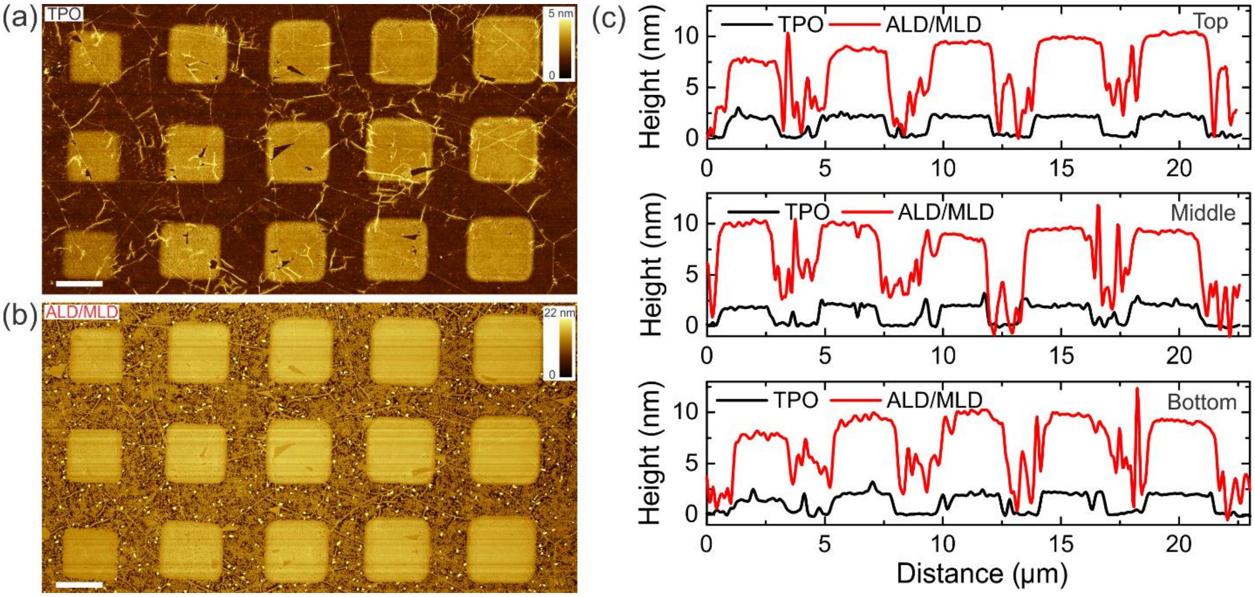

Sample 16:

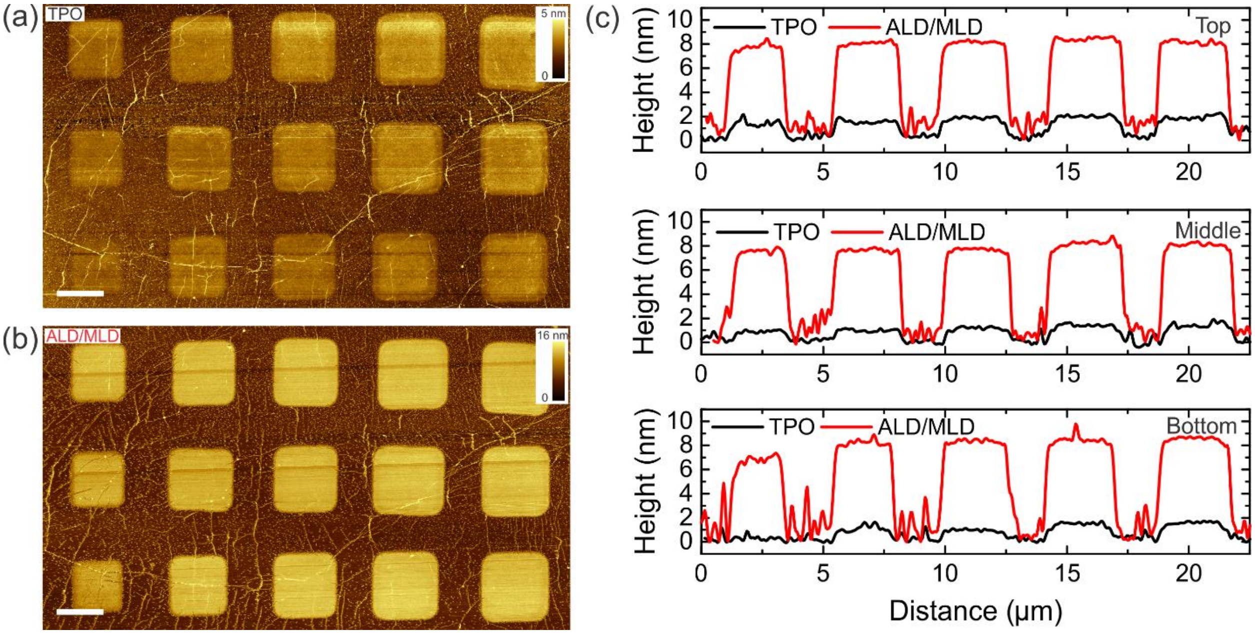

Sample 17:

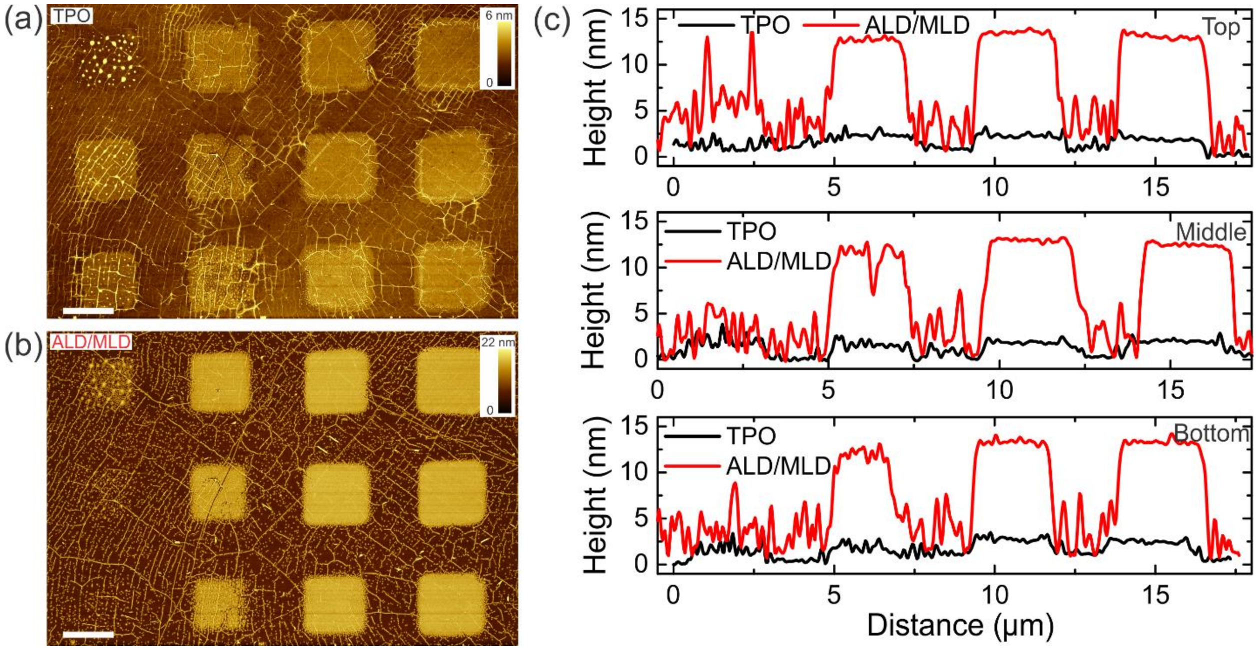

Sample 18:

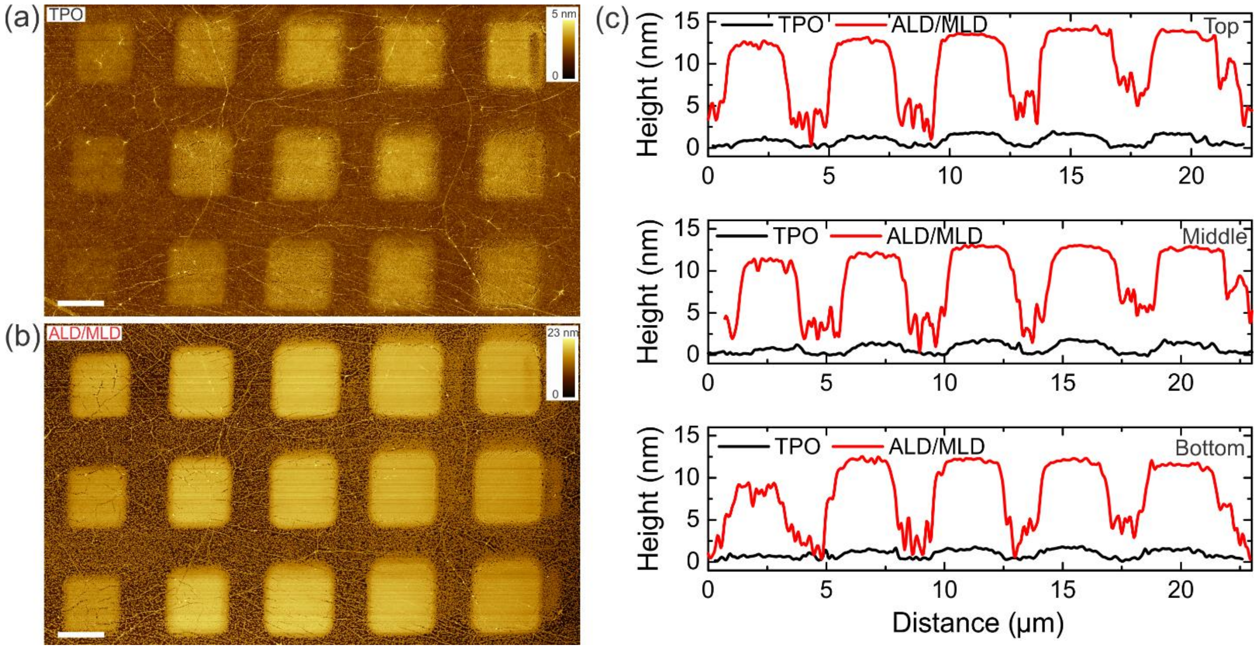

Figure S1. a) AFM images after TPO and b) after ALD/MLD for PMMA and PVAc transferred samples. c) Height profiles of corresponding AFM images after TPO (black) and ALD/MLD (red).

Raman Data for Pristine Graphene and Graphene after TPO and ALD/MLD

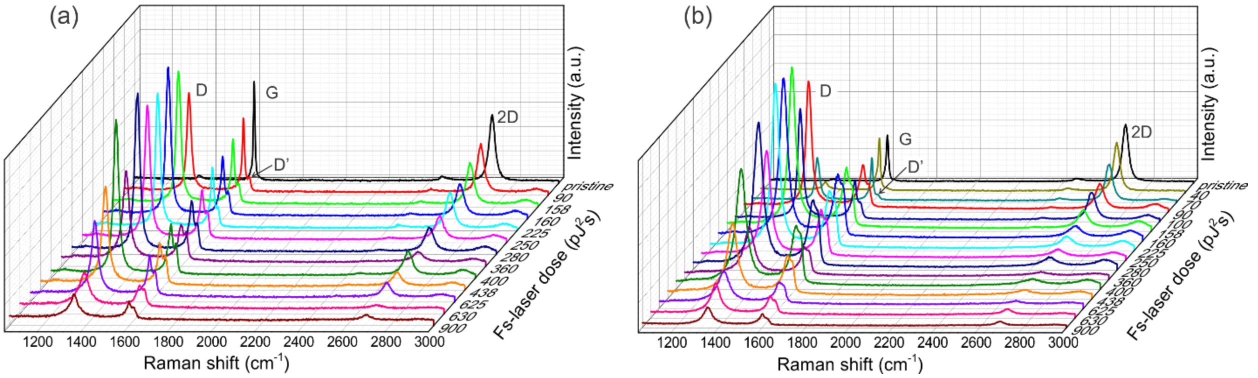

Figure S2. Raman spectra of a) Sample 8 (PMMA) and b) Sample 16 (PVAc) before and after TPO.

Sample 1:

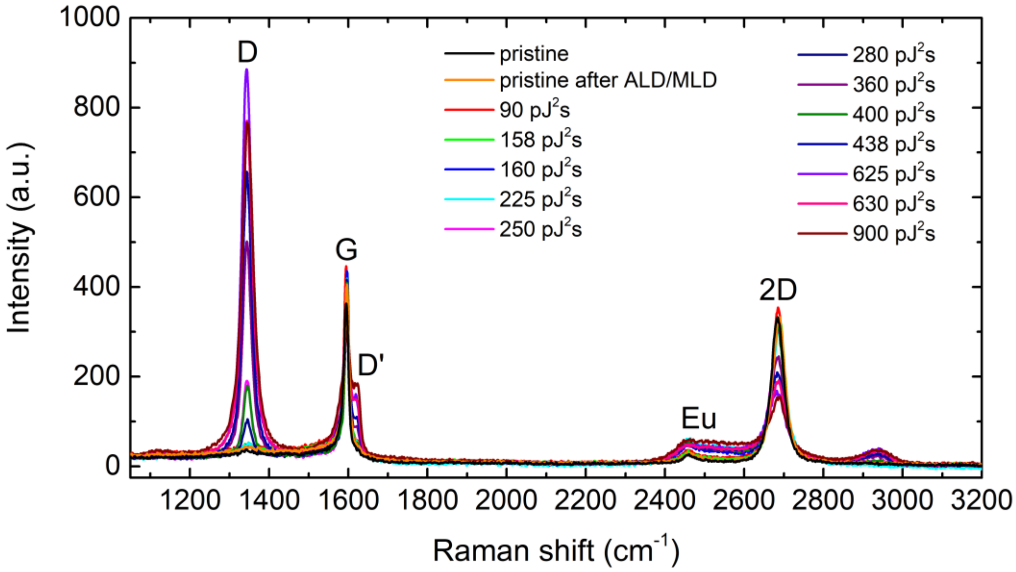

Sample 2:

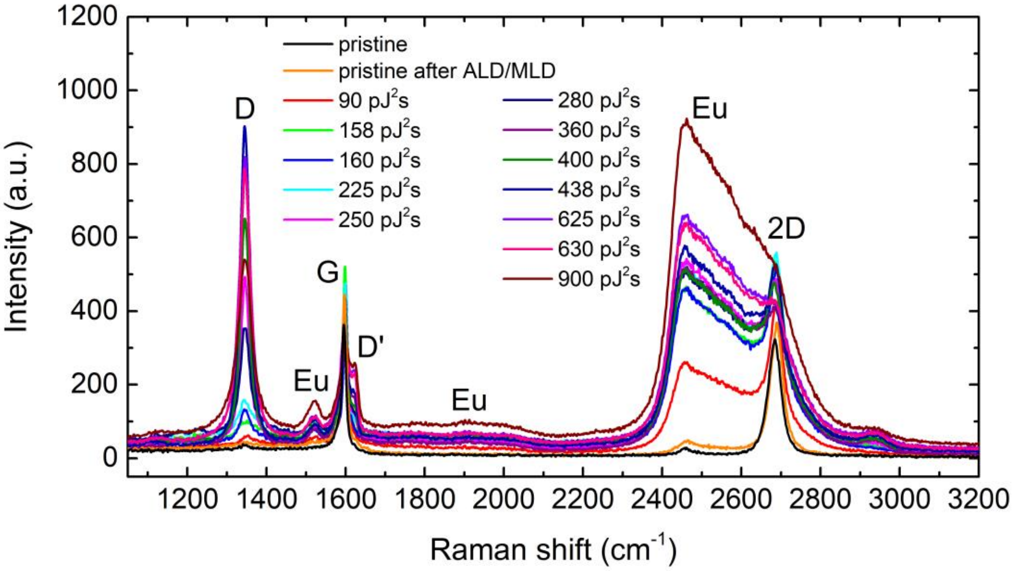

Sample 3:

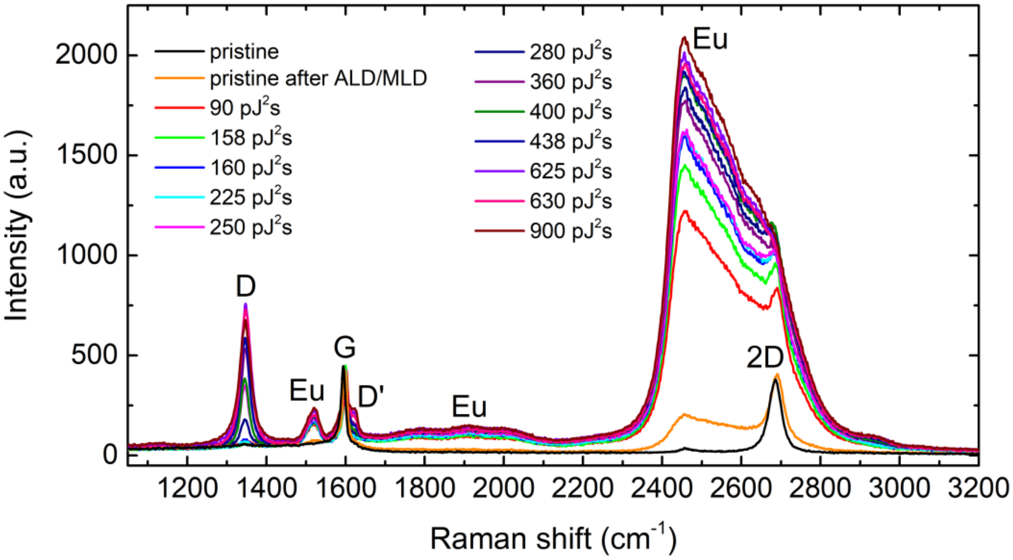

Sample 4:

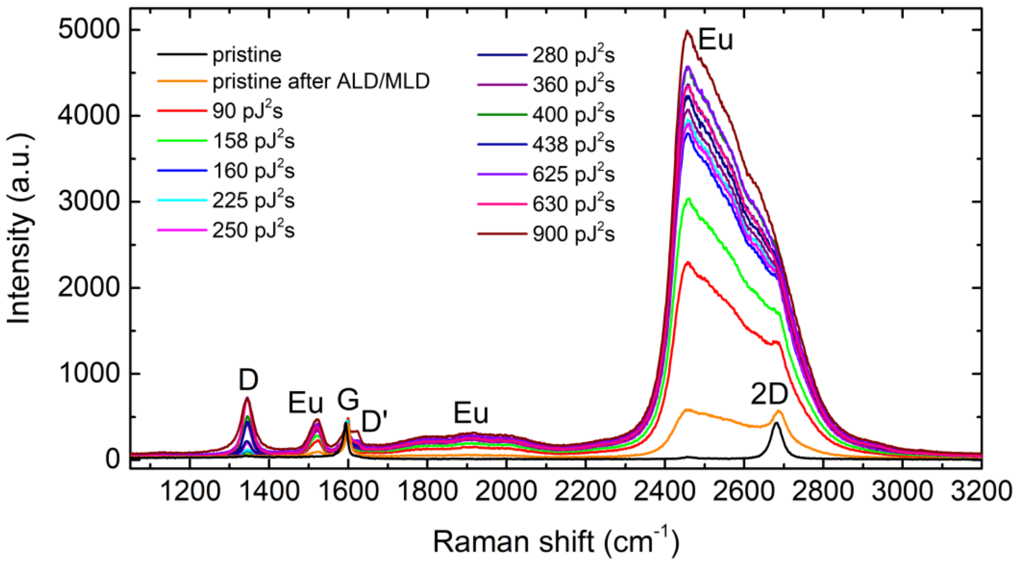

Sample 5:

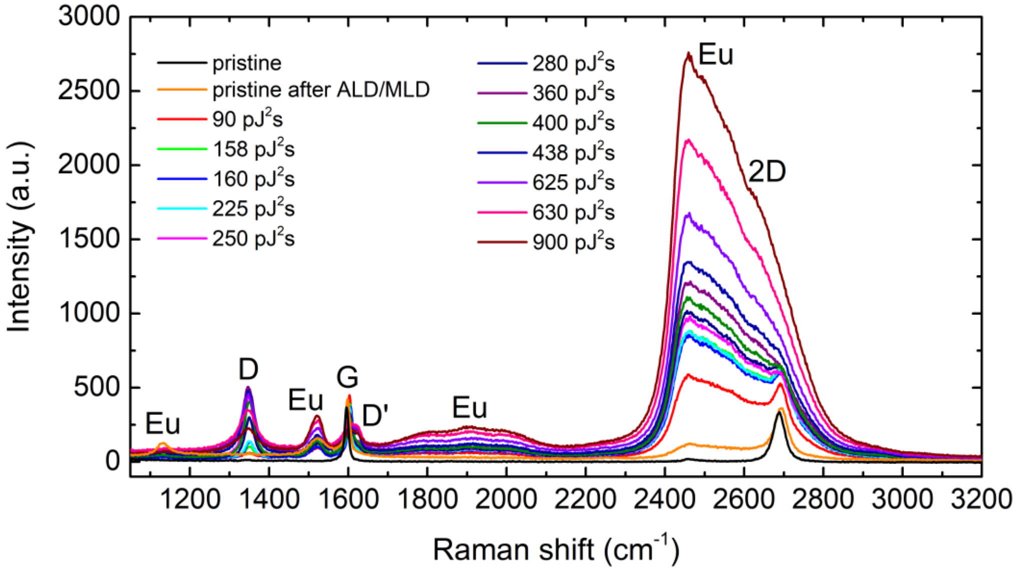

Sample 6:

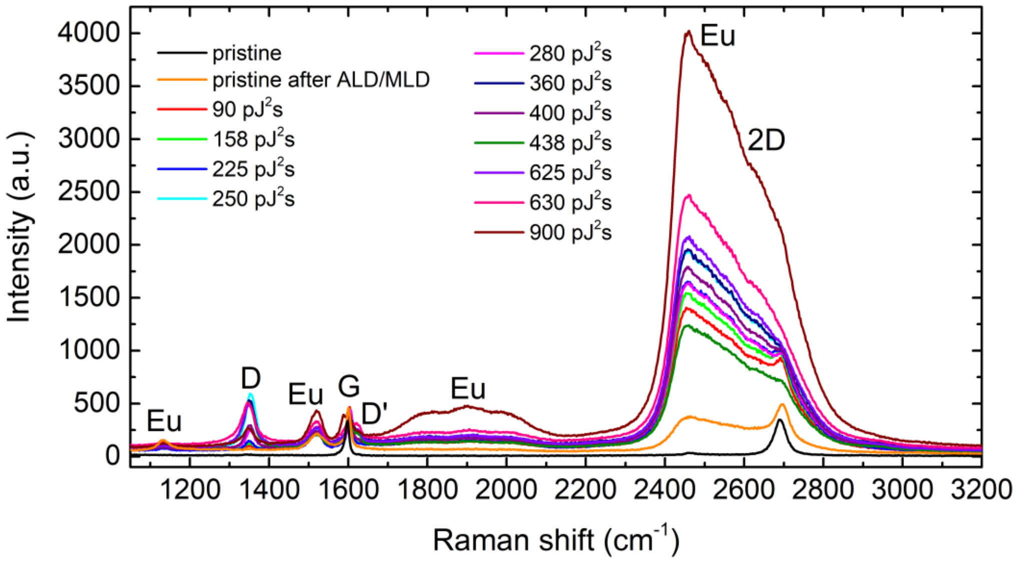

Sample 7:

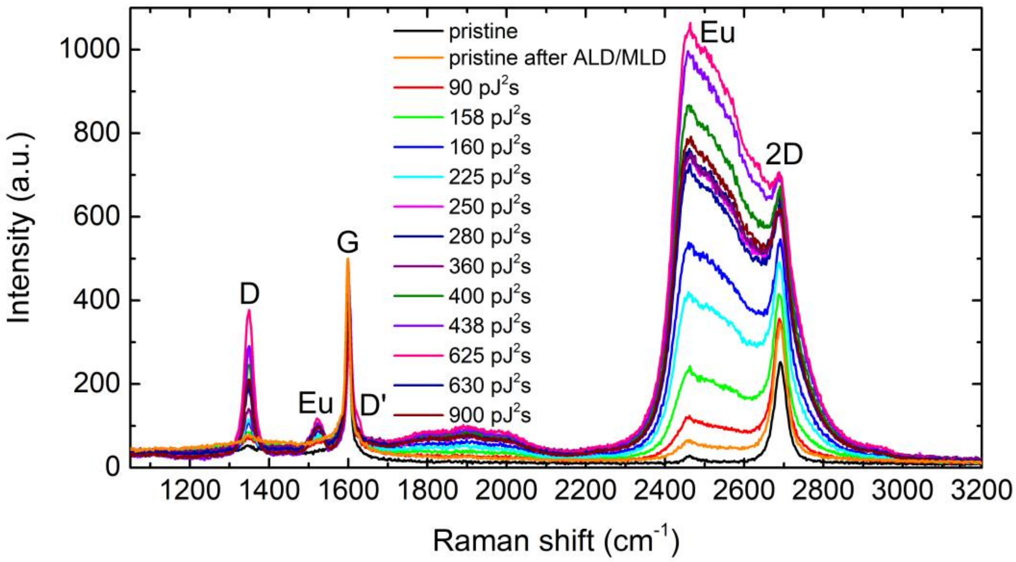

Sample 8:

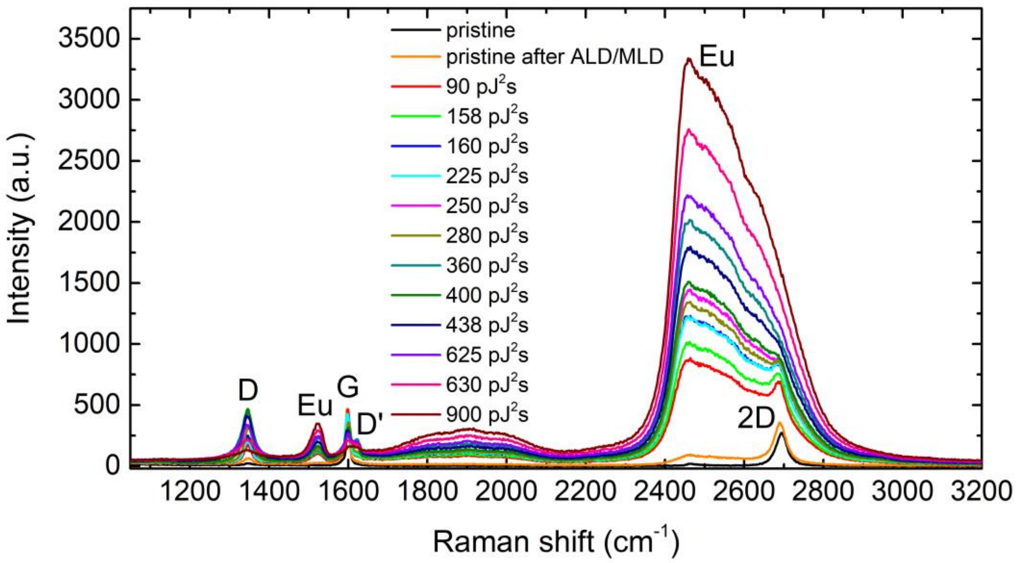

Sample 9:

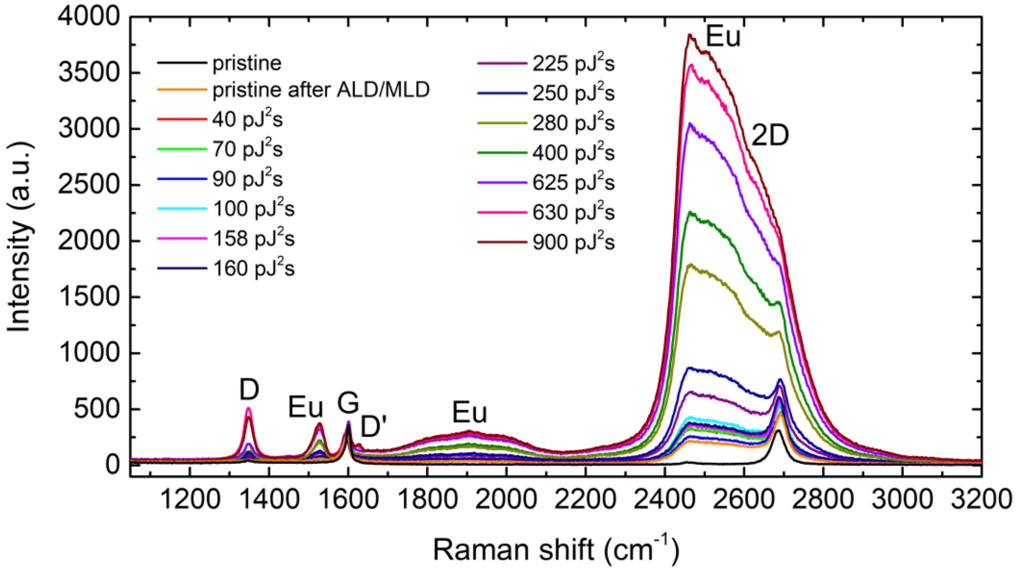

Sample 10:

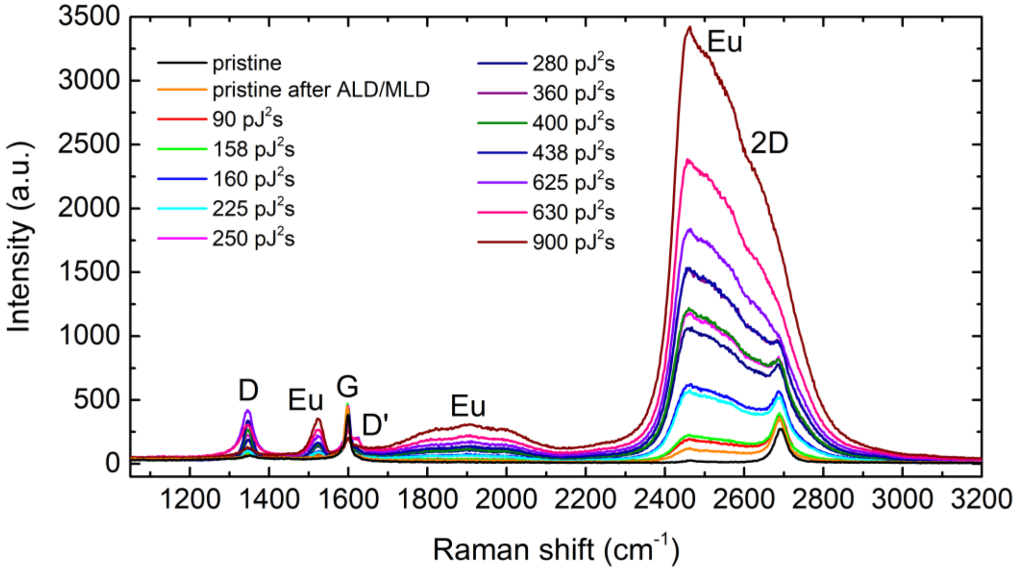

Sample 11:

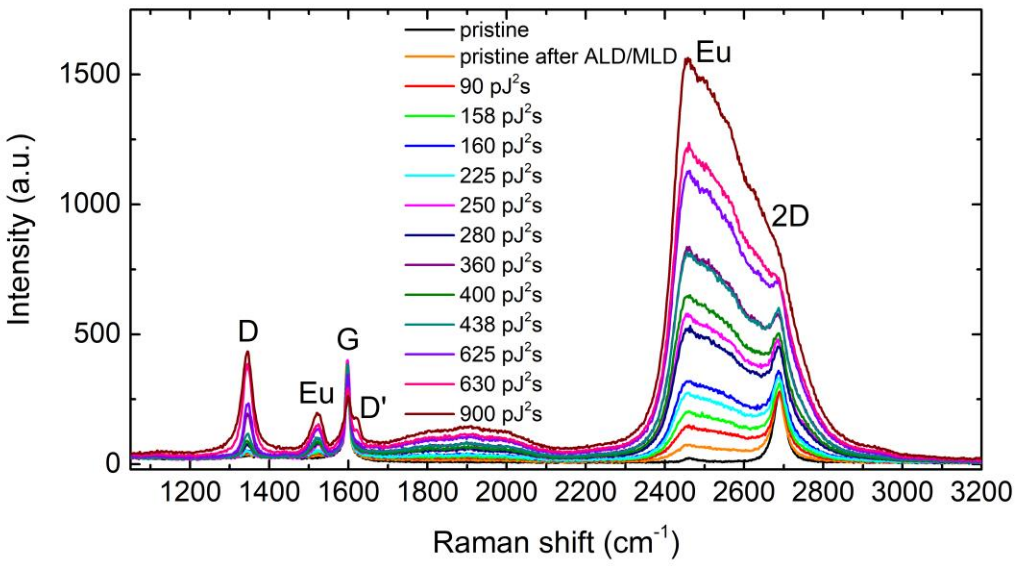

Sample 12:

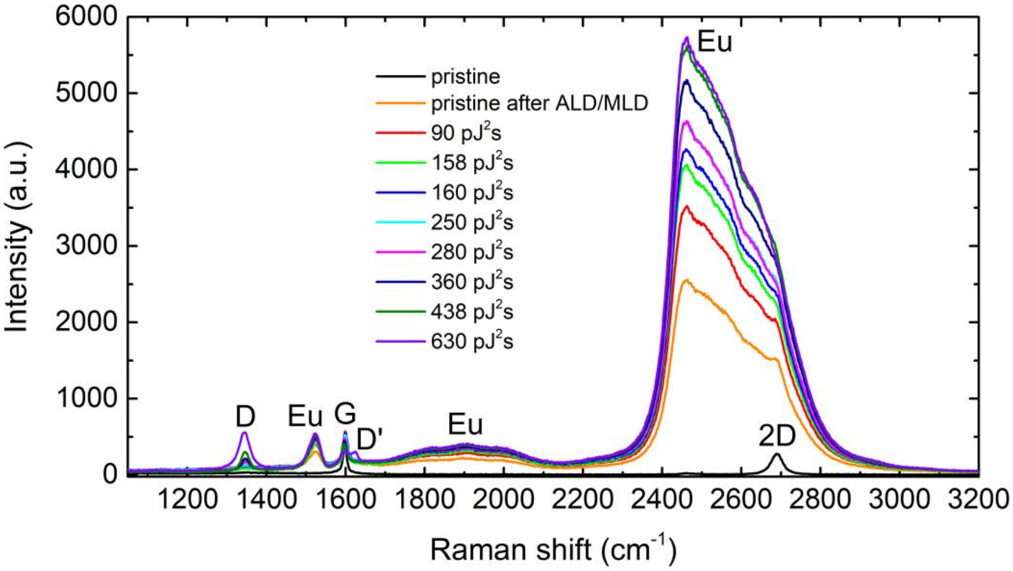

Sample 13:

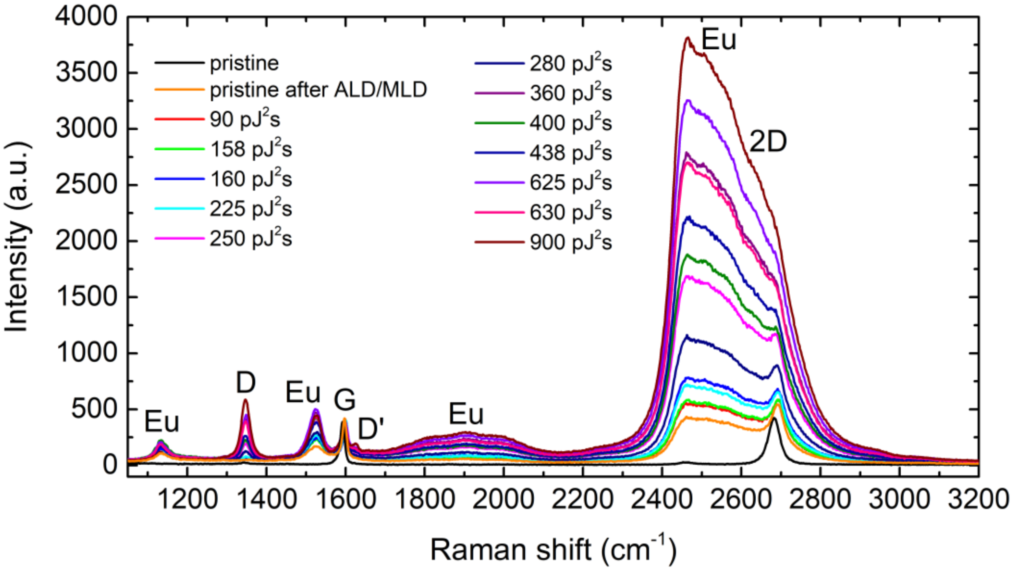

Sample 14:

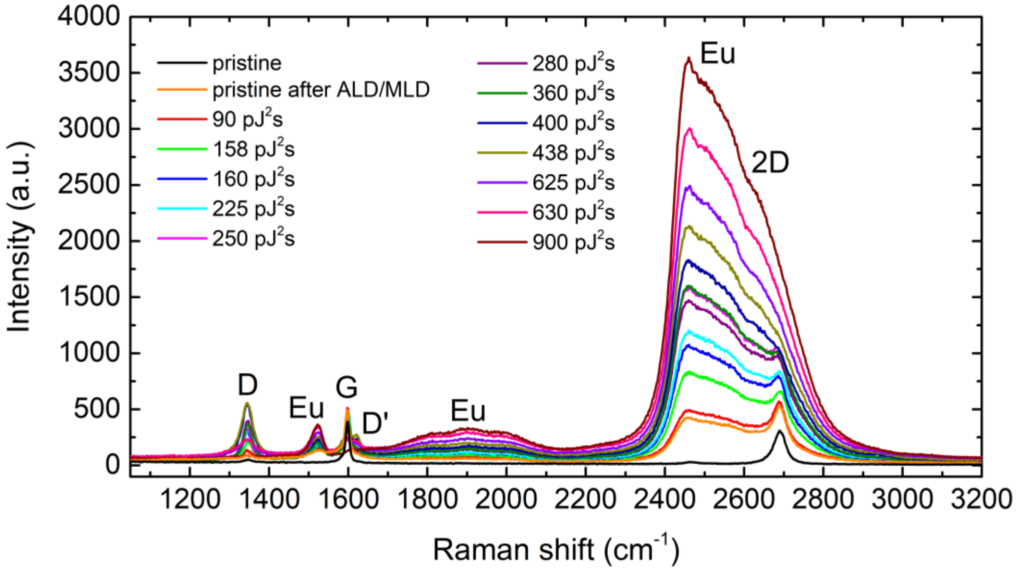

Sample 15:

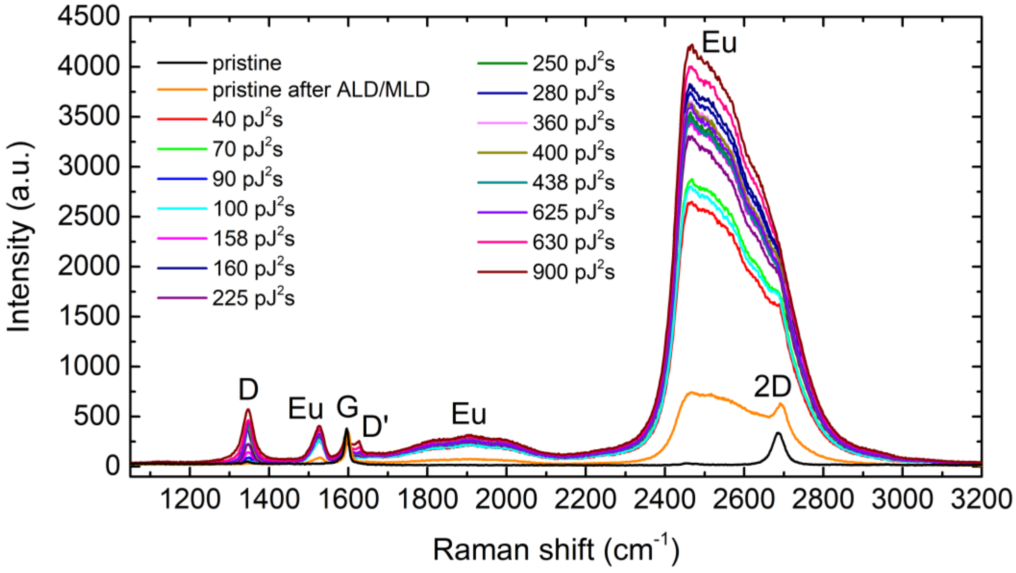

Sample 16:

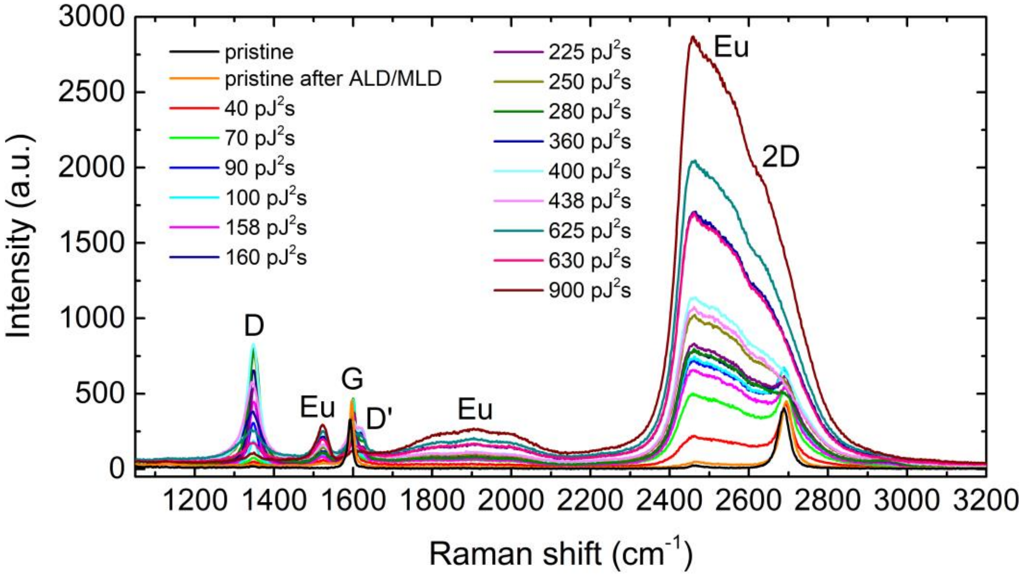

Sample 17:

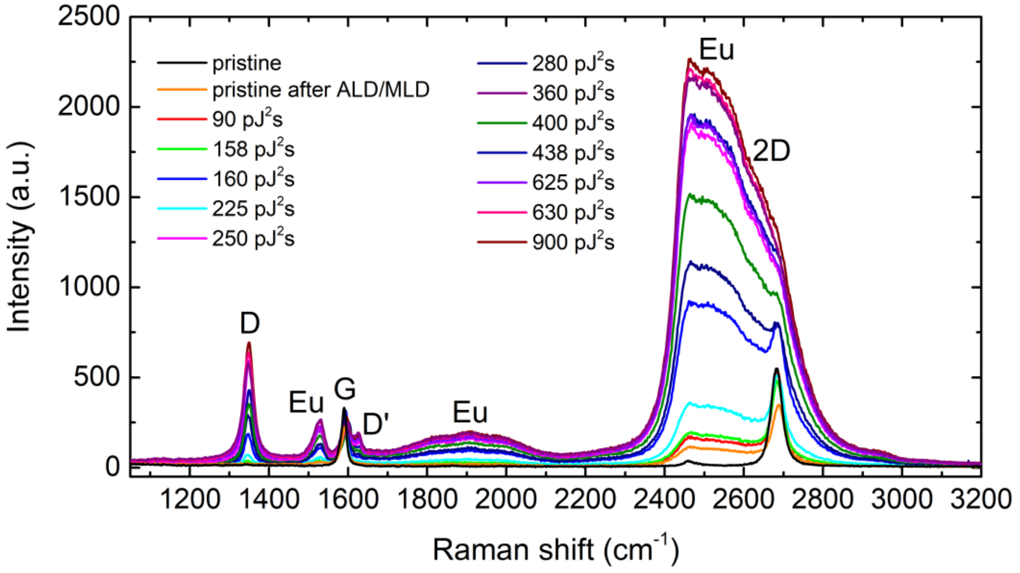

Sample 18:

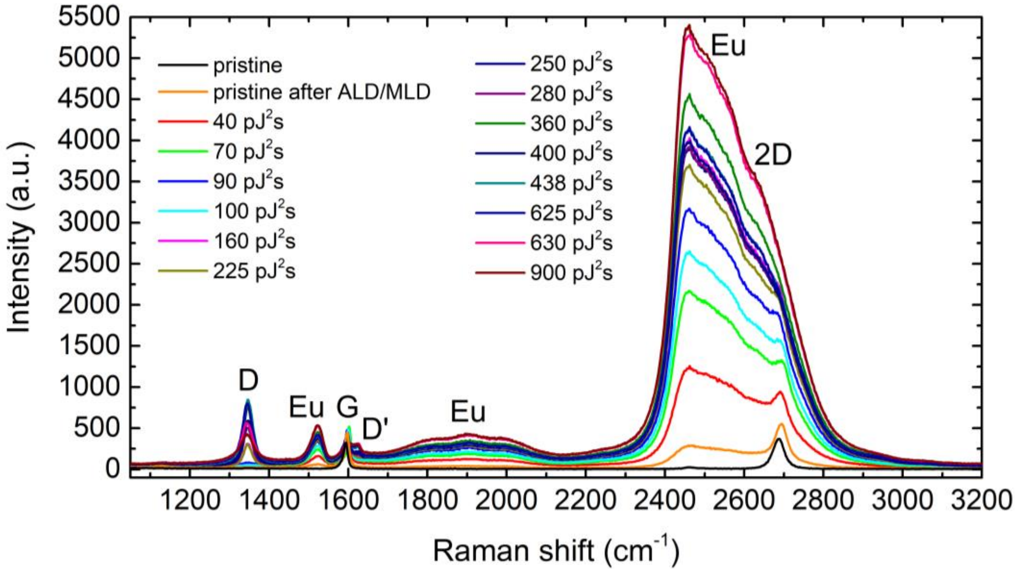

Figure S3. Raman spectra of all the samples after ALD/MLD. The spectrum of pristine graphene before ALD/MLD for each sample is shown for comparison.

FTIR and nano-FTIR spectra

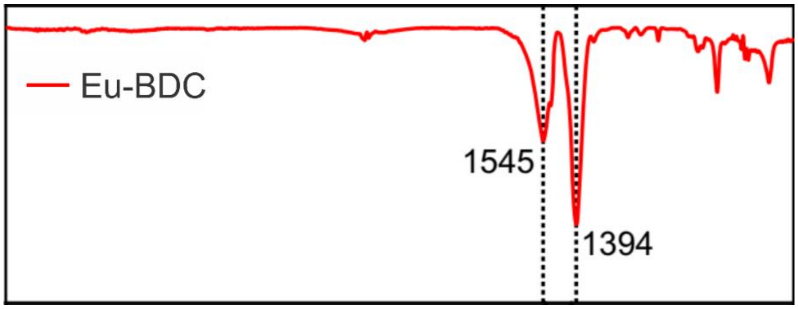

Figure S4. FTIR spectrum of 100 nm Eu-BDC film on Si.

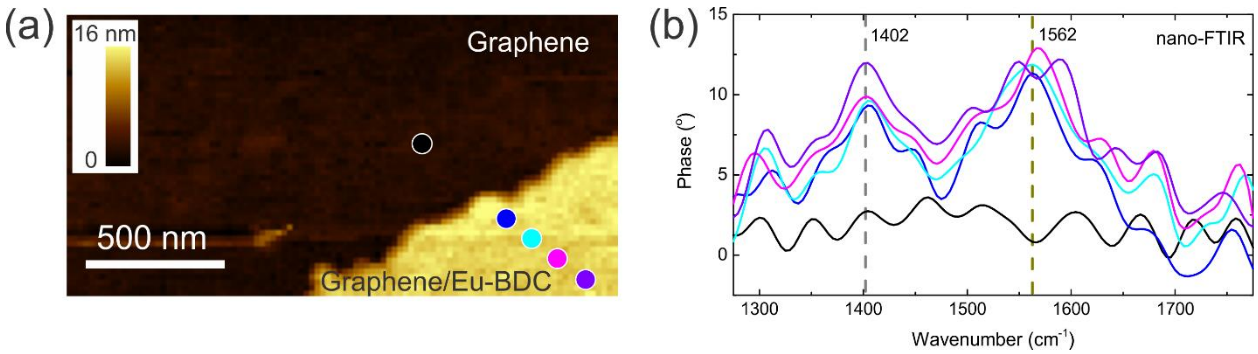

Figure S5. a) AFM height image of graphene and graphene/Eu-BDC thin film measured on Sample 9. b) Corresponding nano-FTIR (IR-SNOM) spectra at different positions marked in a).

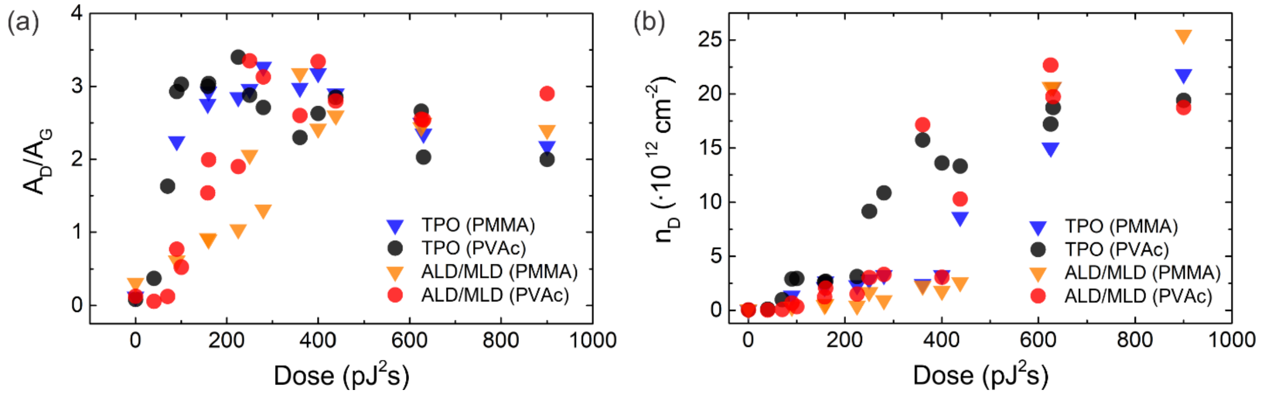

Figure S6. a) A_D/A_G dependence on fs-laser irradiation dose for Samples 8 (PMMA) and 16 (PVAc). b) n_D vs. dose for Samples 8 (PMMA) and 16 (PVAc) after TPO and ALD/MLD of Eu-BDC thin films.

Table S3. Data extracted from the Raman spectra of Samples 8 and 16 after TPO and ALD/MLD.

Sample 8:

Fs-laser dose, pJ^{2}s	Γ_G (TPO), cm^{-1}	Γ_G (ALD/MLD), cm^{-1}	I_D/I_G (TPO)	I_D/I_G (ALD/MLD)	L_D (TPO), nm	L_D (ALD/MLD), nm	n_D (TPO), cm^{-2} *10^{12}	n_D (ALD/MLD), cm^{-2} *10^{12}
0	8.9	10.1	0.04	0.09	50.5	33.7	0.04	0.08
90	14.2	10.9	1.40	0.34	8.6	17.4	1.37	0.33
158	19.6	12.2	2.20	0.5	6.8	14.3	2.15	0.49
160	23.5	13.6	2.73	0.61	6.1	12.9	2.68	0.60
225	20.2	12.3	2.37	0.45	6.6	15.1	2.33	0.44
250	28.3	21.2	2.92	1.72	5.9	7.8	2.86	1.69
280	27.9	16.1	3.34	0.97	5.5	10.3	3.28	0.95
360	21.3	26	2.49	2.32	6.4	6.6	2.44	2.27
400	29	19.5	3.33	1.84	5.5	7.5	3.27	1.80
438	29.4	21	2.46	1.73	3.4	6.2	8.65	2.60
625	31	27.8	1.92	1.51	2.6	2.2	15.02	20.66
630	36.5	26.5	1.5	1.48	2.2	2.2	20.67	20.66
900	39.2	30.1	1.42	1.04	2.1	2.0	21.83	25.51

Sample 16:

Fs-laser dose, pJ^{2}s	Γ_G (TPO), cm^{-1}	Γ_G (ALD/MLD), cm^{-1}	I_D/I_G (TPO)	I_D/I_G (ALD/MLD)	L_D (TPO), nm	L_D (ALD/MLD), nm	n_D (TPO), cm^{-2} *10^{12}	n_D (ALD/MLD), cm^{-2} *10^{12}
0	12.6	11.5	0.05	0.06	43.9	42.7	0.05	0.05
40	14.4	11.8	0.12	0.03	28.8	58.3	0.12	0.03
70	14.5	11.9	1.02	0.08	10.0	35.3	1.00	0.08
90	30.0	16.2	2.97	0.69	5.9	12.2	2.91	0.68

100	26.9	12	3.01	0.35	5.8	17.1	2.95	0.34
158	34.7	16.5	2.70	1.28	6.2	9.0	2.65	1.25
160	39.1	22.1	2.76	2.11	6.1	7.0	2.71	2.07
225	37.7	16.7	3.21	1.55	5.7	8.1	3.14	1.52
250	38.1	29.8	2.42	3.10	3.3	5.7	9.18	3.04
280	40.1	28.0	2.26	3.39	3.0	5.5	10.90	3.33
360	53.5	34.1	1.86	1.74	2.5	2.4	15.74	17.15
400	41.2	31.2	2.03	3.13	2.7	5.7	13.62	3.07
438	38.9	33.9	2.05	2.3	2.8	3.1	13.32	10.31
625	51.0	42.3	1.75	1.37	2.4	2.1	17.22	22.68
630	55.0	40.5	1.65	1.58	2.3	2.2	18.74	19.75
900	55.2	30.3	1.6	1.65	2.2	2.3	19.41	18.74

n_D and L_D were calculated using I_D/I_G ratios according to the equations [S2]:

$$I_D/I_G = 102/L_D^2 \ (L_D > 2r_A),$$

$$I_D/I_G = C_A \frac{r_A^2 - r_S^2}{r_A^2 - 2r_S^2}[exp(-\pi r_S^2/L_D^2) - exp(-\pi(r_A^2 - r_S^2)/L_D^2)] + C_S[1 - exp(-\pi r_S^2/L_D^2)],$$

$(L_D \leq 2r_A)$, where C_A=3.5, C_S=0.87, r_A=3 nm, r_S=1 nm,

and $n_D = 1/L_D^2$.

Additional Data on Eu-BDC Film Thickness and Selectivity on Graphene

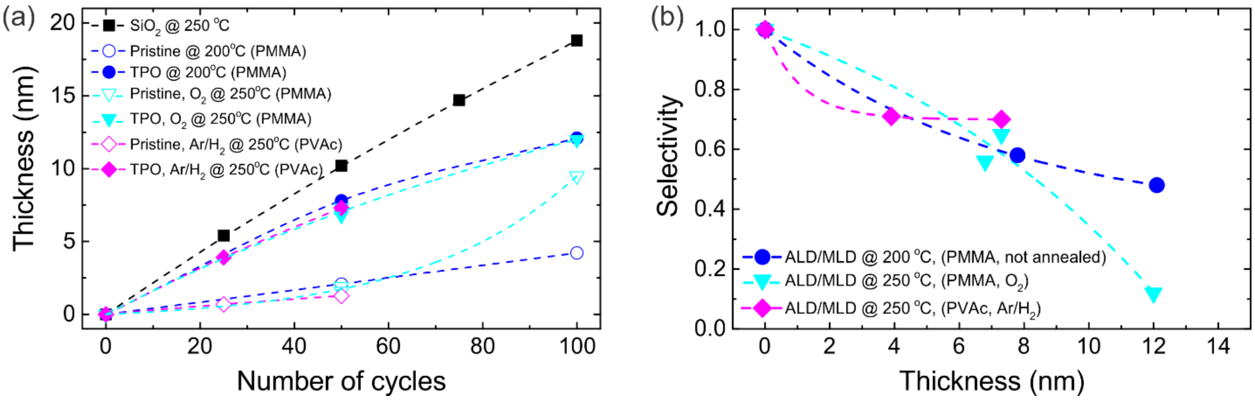

Figure S7. a) Thickness vs. number of ALD/MLD cycles, measured for TPO graphene in the dose range from 225 to 438 pJ^{2}s. b) Selectivity vs. thickness, measured in the dose range from 225 to 438 pJ^{2}s. Dotted lines are guides to the eye.

Graphene Height before and after TPO

The sp^2 to sp^3 change, induced by TPO of the graphene surface, leads to out-of-plane distortion of the flat graphene lattice, which may result in wrinkling, contributing to the observed height

increase. Additionally, while the TPO process at the applied femtosecond laser doses is primarily photochemical, localized photothermal effects can occur. These may induce slight out-of-plane deformation or wrinkling of the graphene sheet on Si/SiO_2, which contributes to the height increase observed by AFM [S3]. Also, oxygen functional groups are introduced on both sides of the graphene layer, effectively increasing the total TPO graphene thickness depending on their surface density and bonding configuration [S4-S6].

A minor contribution may also originate from the partial amorphization of a thin polymer residue layer under laser irradiation, which could locally swell or crosslink. However, it is hard to detect discontinuous residues via Raman spectroscopy, AFM, or micro-XPS. Nevertheless, the measured height of pristine graphene on the Si/SiO_2 substrate was ≈0.4 nm, confirming that no continuous polymer film was present before TPO (see Figure S8). The AFM adhesion map further supports this interpretation, as the oxidized regions show “hydrophilic” contrast similar to the SiO_2 surface.

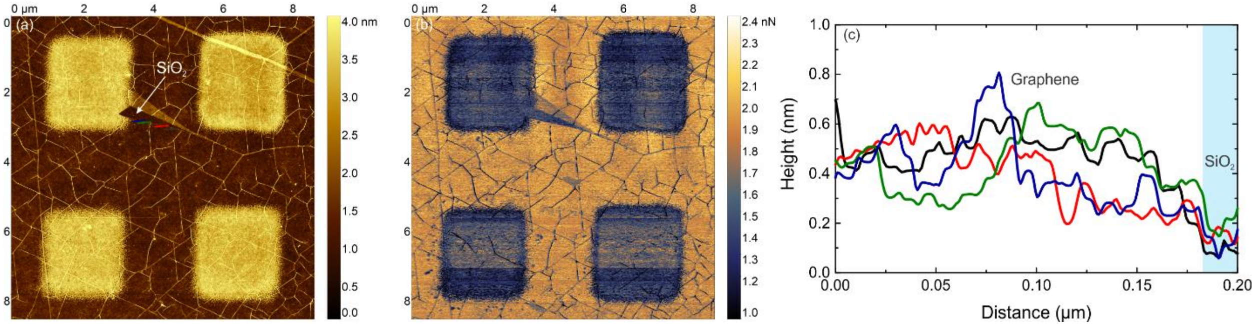

Figure S8. a) AFM height image and b) AFM adhesion image of Sample 10 after TPO. c) Corresponding profiles of pristine graphene on top of Si/SiO_2 measured from a).

TPO and ALD/MLD of Eu-organic Films on MoS_2 and WS_2

We also applied TPO and Eu-BDC ALD/MLD to monolayer MoS_2 and WS_2 to show that our method is extendable to other 2D materials. Monolayer MoS_2 was grown by CVD and transferred onto a Si/SiO_2 substrate using the wet transfer process, followed by several photolithography steps prior to molecular deposition. WS_2 was directly grown by CVD on Si/SiO_2 without further processing.

For MoS_2, the AFM topography (Figure S9a) confirmed localized surface modification within the laser-irradiated areas, indicating a height increase of ≈1.5-2 nm. The Eu-organic film thickness after ALD/MLD was ≈6-7 nm (Figure S9b,c). The corresponding Raman map (Figure S9d) illustrates the spatial distribution of the Eu-related band at 612 nm. Some patterned areas appear indistinct due to polymer residues from photolithography, but the Raman spectra provide clear evidence of selective ALD/MLD growth. In pristine MoS_2, the E^1_{2g} and A_{1g} bands show a slight redshift after ALD/MLD, and weak Eu-related bands appear due to nucleation on polymer residues resulting from photolithography processes. For TPO MoS_2, both bands redshift and broaden after TPO, followed by a small blueshift, primarily in the E^1_{2g} band, after ALD/MLD. Eu-related peaks are more prominent in TPO MoS_2, while no additional bands corresponding to MoO_3 are observed,

indicating sub-threshold oxidation. The spectral changes (broadening and redshift) are consistent with non-thermal oxidation and oxygen incorporation reported for early-stage O_2 plasma treatment [S7]. The introduced oxygen groups provide reactive sites for precursor chemisorption, enabling the selective growth of Eu-BDC.

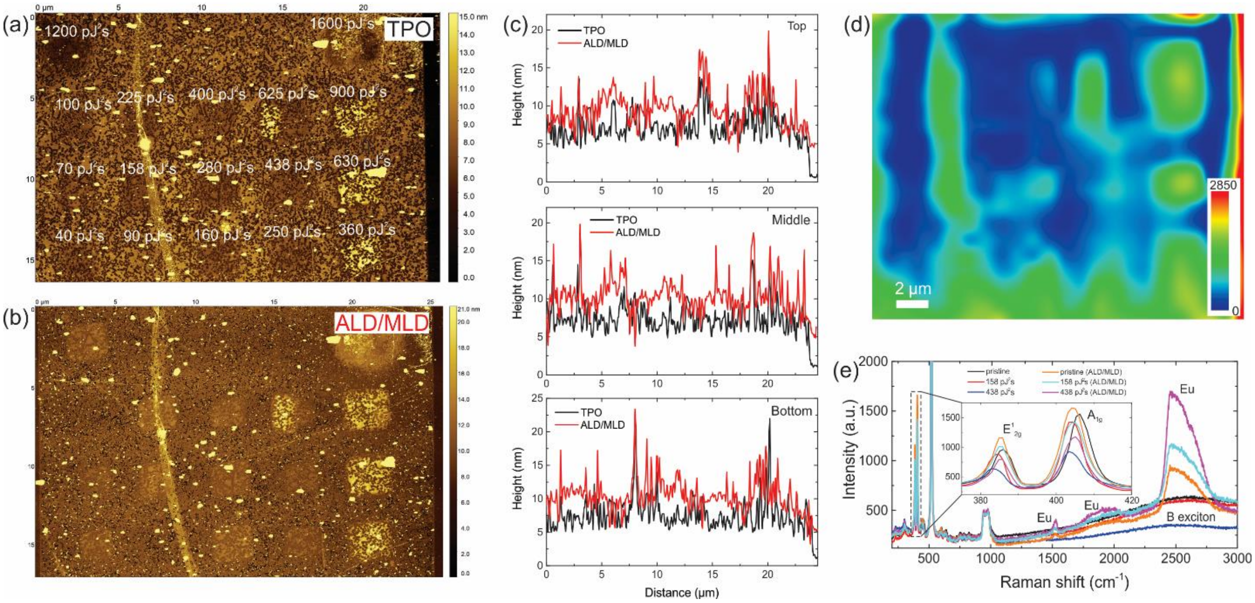

Figure S9. a) AFM topography image for monolayer MoS_2 after TPO at different doses mentioned in the image. b) AFM topography image measured after ALD/MLD of 7 nm Eu-BDC film. c) AFM profiles before and after ASD measured from a) and b). d) Micro-PL map of the Eu-BDC film after 100 ALD/MLD cycles, measured at 612 nm and excited by a 532 nm CW laser. e) Raman spectra of pristine MoS_2 and two TPO MoS_2 areas before and after 100 ALD/MLD cycles of Eu-BDC.

For WS_2, AFM revealed well-defined surface modification in irradiated regions (Figure S10a-c). The ALD/MLD process demonstrated high selectivity due to the cleaner CVD surface. Minor WS_2 degradation near flake edges likely resulted from prolonged exposure to ambient humidity. The Raman map (Figure 10d) shows distinct areas where Eu-BDC deposition occurred. The E^1_{2g} and A_{1g} Raman bands display decreased intensity and moderate broadening, while the A-exciton exhibits a redshift of about 20 meV (from 631.8 to 638.3 nm) after both TPO and ALD/MLD (Figure S10e). This shift can be attributed to dielectric screening by the Eu-BDC layer and/or a small strain, with an additional contribution from trion formation. In pristine WS_2 regions, no Eu modes or Raman shifts were detected, confirming negligible nucleation on the inert basal plane. These spectral features suggest the formation of WO_3-like surface domains, similar to those observed in UV-ozone-treated WS_2 [S8]. The Eu-BDC films were deposited predominantly on TPO WS_2 regions, demonstrating selectivity comparable to that achieved for graphene.

Using the same Eu-BDC ALD/MLD conditions as for graphene (250°C), the TPO-induced active sites on MoS_2 and WS_2 remain sufficiently stable to initiate and sustain selective nucleation during the initial cycles. Although we did not perform a dedicated temperature-window study for MoS_2 and WS_2, oxide-like surface domains are not expected to desorb at 250°C. In contrast, we avoided

growth temperatures above 300°C due to partial decomposition of oxygen-containing groups in TPO-treated graphene (and other 2D materials), which reduces selectivity.

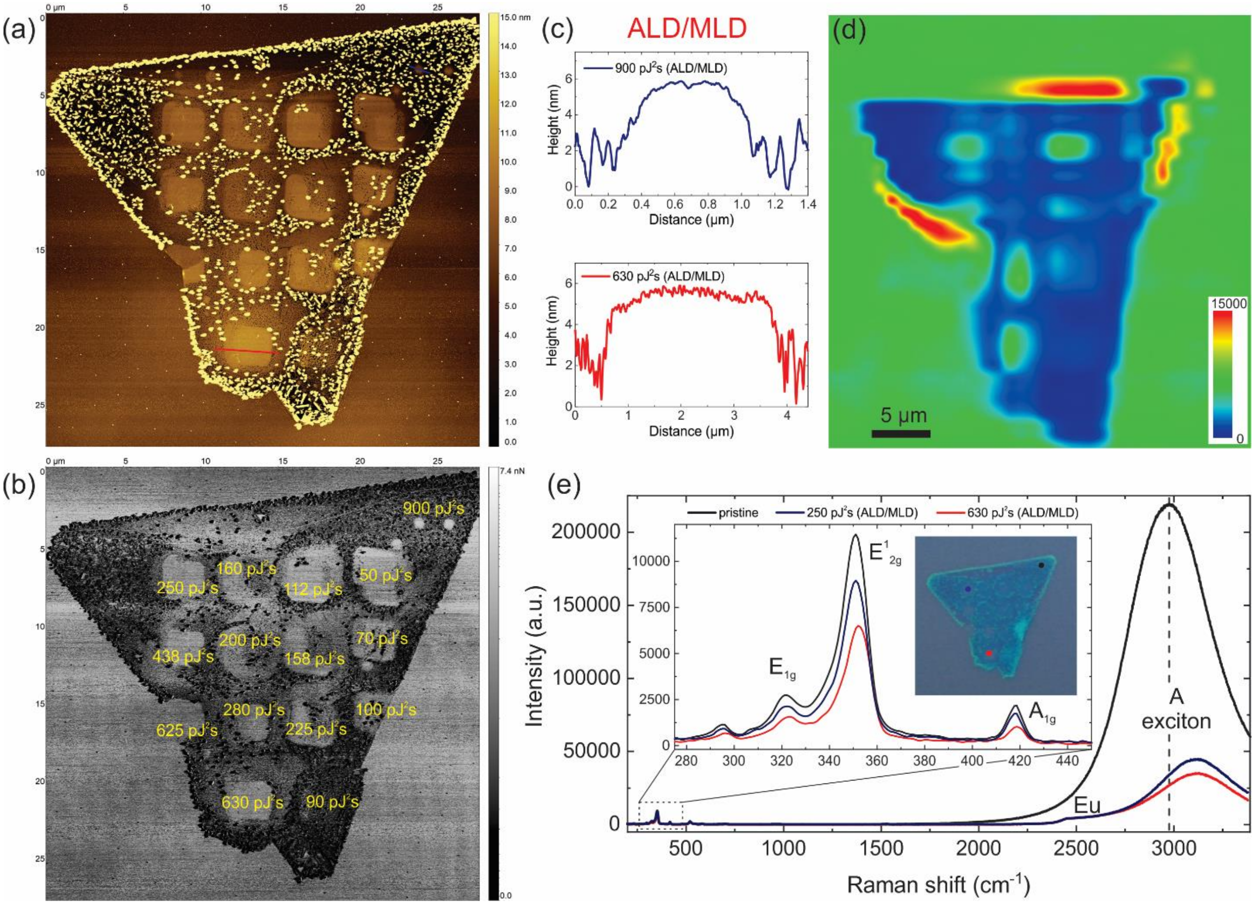

Figure S10. a) AFM topography image for monolayer WS_2 after ALD/MLD of 3 nm Eu-BDC film. b) AFM adhesion image for WS_2 after ALD/MLD of 3 nm Eu-BDC film. c) AFM profiles after Eu-organic film deposition measured from a) for two fs-laser doses. d) Micro-PL map of the Eu-BDC film after 50 ALD/MLD cycles, measured at 612 nm and excited by a 532 nm CW laser. e) Raman spectra of pristine WS_2 and two TPO WS_2 areas after 50 ALD/MLD cycles of Eu-BDC.

Table S4. Comparison of ALD, MLD, and AS-ALD Approaches on Bulk, 1D and 2D Materials.

	Activation / Inhibition Strategy	Material / Substrate	Selecti-vity	Lateral Resolution	Process temperature (ºC)	Applications	Limitations	Ref.
**Bulk**	Dimethylamino-trimethylsilane inhibitor	TiO_2 on TiN and SiO_2	~90% at 10 nm	<50 nm, lithography-defined	150	3D structures, FET	Require plasma etching to remove undesired TiO_2 nucleus, uniformity < 90%	[S9]
	MoF_6 passivation	ZnO or TiO_2 on Mo	~90 and 95% at 10 nm	<200 nm, lithography-defined	250	Nanoribbons, stop layers	Passivation does not work for Al_2O_3, selectivity loss after 10 nm film	[S10]
	SAM	Polyurea on Si/SiO_2	~92% at 10 nm	Lithography-defined	23	3D structures	Long SAM growth and pulse/purge times	[S11]
	Ti inhibitor	TiO_2 on SiO_2	~95% up to 49 nm	Lithography-defined	180	3D structures	Selectivity drops with depth	[S12]
	Indicone inhibitors	ZnO on Si	81% at 5 nm	Lithography-defined	-	3D structures	Require 450-600 °C annealing before ALD, and reactive ion etching after	[S13]
	Alucone and indicone inhibitors	Ru on Si	-	Lithography-defined	-	3D structures	Require 450-600 °C annealing before ALD, and reactive ion etching after	[S14]
	Ion implantation	Pt on Si	99% at 10 nm	Lithography-defined	250	3D structures	CF_X blocking degrades after ~500 cycles, blocking requires optimization for other materials	[S15]
	E-beam irradiation	ZnO on graphite	70% at	<42 nm	150	-	Low selectivity due to α-C contamination under e-beam	[S16]
	Remote Ar plasma	HfO_2 on graphite	-	-	200-300	FET	Defects after ALD	[S17]
**Graphene**	GO Seeding layer	Al_2O_3 on graphene	-	-	150	FET	The GO layer can limit charge transfer and carrier coupling	[S18]
	Oxygen plasma and polyethylenimine	Al_2O_3 on graphene	-	-	100	Temperature sensor	Transconductance decreases after encapsulation	[S19]
	UV-ozone	Al_2O_3 on graphene	-	-	100	FET	Cleaning of polymer residues at 5s UV, non-continuous Al_2O_3 film	[S20]
	-	Al_2O_3 on graphene	-	-	300	FET, sensor	Thick films, defects in graphene, and cracks in Al_2O_3 after ALD	[S21]
	Precleaning with H_2O	Al_2O_3 on graphene	-	-	100	Emitter	Low graphene quality	[S22]
	Al seeding layer	Al_2O_3 on graphene	-	-	300	Phototransisto r	Low responsivity due to defects	[S23]
	Cu or Ni-Au substrate	Al_2O_3 on graphene	-	-	80-200	FET	Continuous growth only on graphene on Cu	[S24]
	Oxygen plasma	Al_2O_3 on graphene	-	-	100	FET	Defects in graphene	[S25]

	Oxygen plasma + hBN	Al_2O_3 on graphene	-	-	300	FET	Non-scalable	[S26]
	Al seeding layer	Al_2O_3 on graphene	-	-	300	FET	Defects due to the Al seeding layer	[S27]
	XeF_2 gas treatment	Al_2O_3, HfO_2, ZnO on graphene	-	-	200	FET	Require heating up to 400°C to break C-F bonds	[S28]
	Nitrogen and oxygen plasma	AlOx+Al_2O_3 on graphene	-	-	200 and 300	FET	N_2 plasma induces doping of graphene	[S29]
	Oxygen plasma	AlO_X on graphene	-	-	150	FET	Not optimal gate/dielectric interface to observe quantized conductance	[S30]
	PTCDA SAM	HfO_2 and Al_2O_3 on graphene	-	-	100 and 225	Capacitor, FET	Holes in the HfO_2 layer	[S31]
	Functionalization by p-hydroxyphenyl anchor groups	ZnO/Al_2O_3/CuO on graphene	~100% at 4 nm	Lithography-defined	250	3D structures	Hardening of PMMA at grown T	[S32]
	α-C layer by e-beam	HfO_2 on graphene	-	-	90	FET	Defects and trap states after electron beam irradiation	[S33]
	-	Pt on graphene	-	-	300	Gas sensor	High selectivity only for grain boundaries and step edges	[S34]
	Fs-laser	ZnO on graphene	~92% at 6 nm	~300 nm	150-200	UV photodetectors	Require post-annealing at 300°C to restore graphene to its initial state	[S35]
**CNT**	Physical adsorption	HfO_2 on CNT	-	-	90	FET	Polymer residues affect the deposition	[S36]
	-	Alucones and zincones on CNT	-	-	150	Composite	Non-uniform coatings	[S37]
**2D layered materials**	Si seeding layer, low T growth	HfO_2 and Al_2O_3 on MoS_2 and WSe_2	-	-	200	FET	Trap states due to Si evaporation	[S38]
	He+ beam	HfO_2 on MoS_2	91% at 3.8 nm	<10 nm	280	FET	While the process works on a bulky MoS_2, the monolayer gets damaged	[S39]
	Oxygen plasma	HfO_2 and Al_2O_3 on MoS_2	-	-	100-300	FET	Damage to MoS_2 during ALD	[S40]
	Si seeding layer	HfO_2 and Al_2O_3 on MoS_2	-	-	300	FET	Damage to MoS_2 due to Si deposition	[S41]
	Au substrate	HfO_2 and Al_2O_3 on MoS_2	-	-	250 and 290	-	ALD only on a metal substrate, incomplete coverage for thin HfO_2	[S42]
	Ozone	Al_2O_3 on a sacrificed graphite or WSe_2	-	-	80 and 150	FET	Non-scalable	[S43]
	Mica substrate	Al_2O_3 and HfO_2 on mica on MoS_2 and BP	-	-	150	FET	Non-scalable	[S44]

Acetylacetone	WS_2 on various dielectrics	95% at 1 nm	Lithography-defined	250	FETs	Require post-annealing at 450°C to improve crystallinity	[S45]
GO seeding layer	HfO_2 on WS_2	-	-	300	FET	GO layer can limit charge transfer and carrier coupling	[S46]
-	aBN on MoS_2	-	-	65 and 250	Quantum well structures	Non-scalable	[S47]
-	Al_2O_3 on WS_2	-	-	200	-	High selectivity only for grain boundaries and step edges	[S48]
-	HfO_2 on MoS_2	-	-	180	Phototransisto r	Slow decay times due to oxygen vacancies or trap states	[S49]
AlO_X	HfO_2 on MoS_2	-	-	50-125 for AlO_X 200 for HfO_2	FET	V_{th} variation between FETs	[S50]
AlO_X	Al_2O_3 on MoS_2	-	-	50 for AlO_X 200 for HfO_2	FET	EOT > 1.3 nm, pinholes in seeding layer	[S51]
Sb_2O_3 seeding layer	HfO_2 on MoS_2	-	-	140	FET	V_{th} variation between FETs	[S52]
PTCDA SAM	HfO_2 on graphene, MoS_2 and WSe_2	-	-	150	FET	Transfer residues degrade the film quality, high contact resistance	[S53]
-	Al_2O_3 on MoS_2 and WS_2	-	-	180	Gas sensor	High selectivity only for grain boundaries and step edges	[S54]
Lateral superlattice	Al_2O_3 on $MoSe_2$	~95% at 10 nm	Defined by lateral superlattice width	170	FET	Works only on the $MoSe_2$ surface. HfO_2, Ru, Te, and Sb_2Se_3 show lower selectivity	[S55]
Aminosilane inhibitors	Ru on MoS_2	-	Defined by a flake size	283	3D structures	Selectivity only towards Ru and Pt versus SiO_2, no selectivity for HfO_2	[S56]
Fs-laser	Eu-BDC on graphene, MoS_2, WS_2	~92% at 10 nm	<300 nm	200, 250	FET, Phototransisto r	Damage to 2DM at high laser doses, polymer traces affect the selectivity	**This work**

Abbreviations: PTCDA – perylene-3,4,9,10-tetracarboxylic dianhydride, SAM – self-assembled monolayer, α-C layer – amorphous carbon layer, BN – boron nitride, BP – black phosphorus, GO – graphene oxide, FET – field-effect transistor, V_{th} – threshold voltage, EOT – equivalent oxide thickness

The Work Function and Raman G and 2D Band Shifts after TPO and ALD/MLD

In Kelvin probe force microscopy (KPFM), we measured surface potential as the contact potential difference (V_{CPD}) between the conductive Au tip and the sample. The V_{CPD} is related to the work function (WF) as:

$$V_{CPD} = (\Phi_{tip} - \Phi_{sample})/e,$$

where Φ_{tip} and Φ_{sample} are the tip and sample work functions, and e is the elementary charge. To obtain absolute WF values, we calibrated the effective tip WF using a reference with a known WF. Using Au as the reference, we first measured V_{CPD_Au} and calculated Φ_{tip}:

$$\Phi_{tip} = \Phi_{Au} + e \times V_{CPD\_Au}$$

Then, for the graphene sample, the V_{CPD} was measured with the same tip and identical settings, and the WF was calculated as:

$$\Phi_{sample} = \Phi_{tip} - e \times V_{CPD\_sample} = \Phi_{Au} + e \times (V_{CPD\_Au} - V_{CPD\_sample}).$$

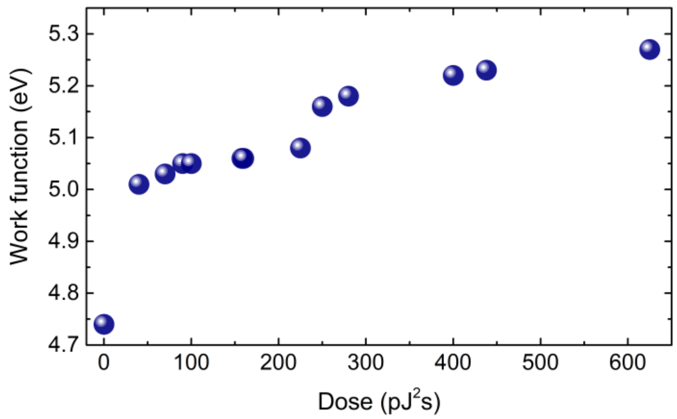

Figure S11. Work function dependence on fs-laser dose after TPO for Sample 16.

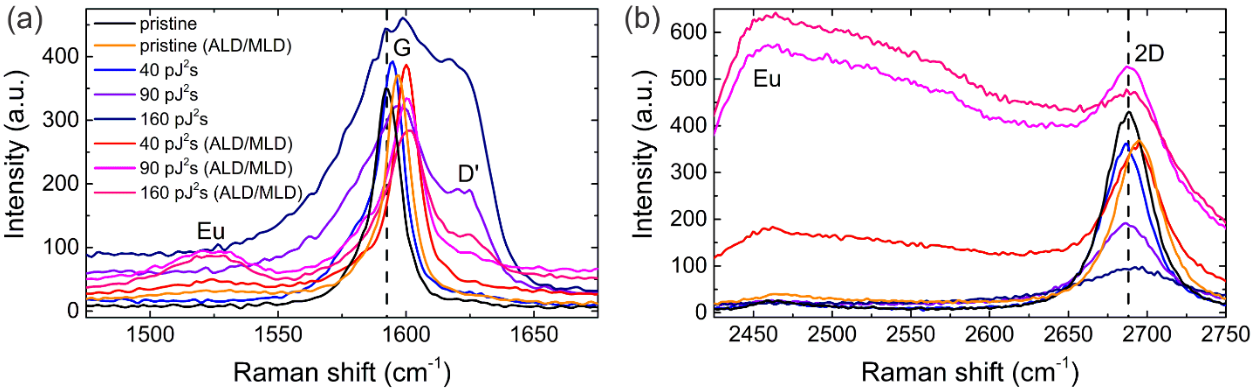

Figure S12. Enlarged Raman spectra of the a) G and b) 2D bands of Sample 16 before and after ALD/MLD of Eu-BDC for pristine graphene and three TPO doses.

Micro-PL Spectra for Graphene after ALD/MLD of Eu-BDC Films

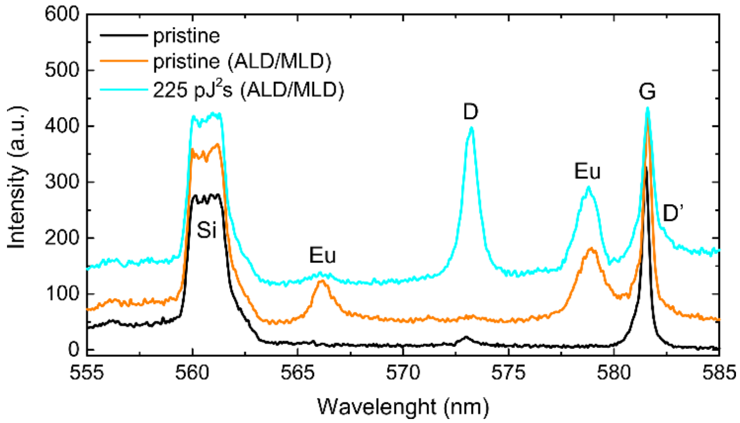

Figure S13. Micro-PL spectra for Sample 18 after ALD/MLD, containing a 566 nm peak.

Emission Spectra of Eu-BDC Thick Film on Si

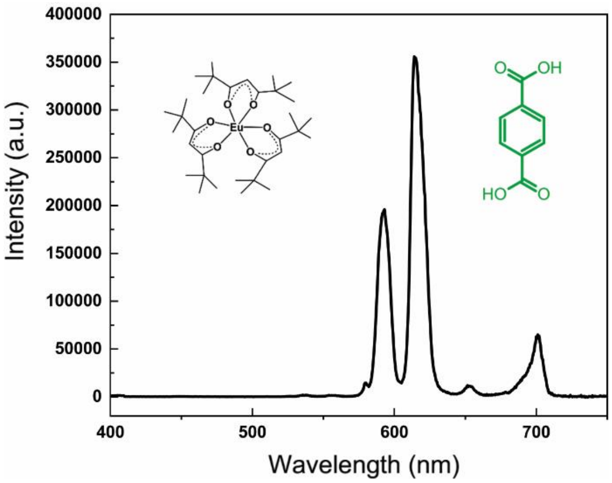

Figure S14. The emission spectrum of a 100 nm Eu-BDC film on Si, excited by a 250 nm Xe arc lamp.

PL Spectra at Different Excitation Powers

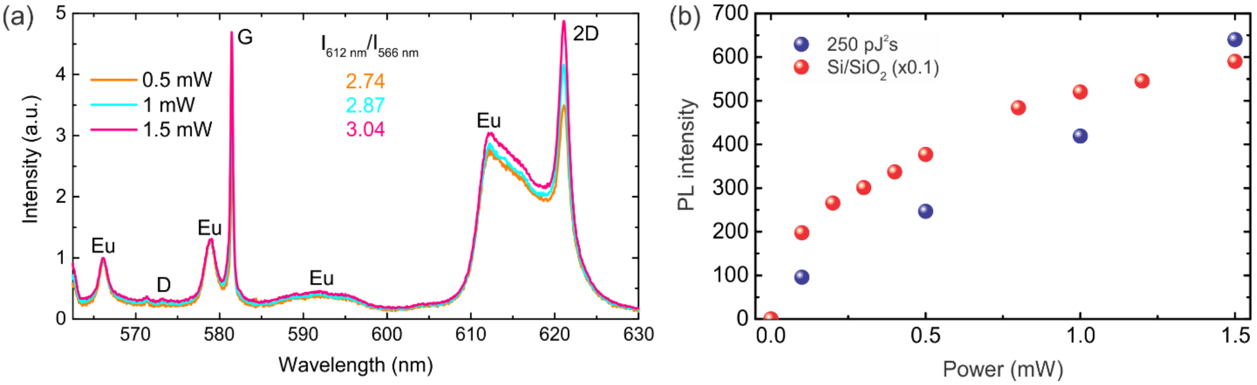

Figure S15. a) Normalized spectra at 566 nm for Sample 18 measured on pristine graphene after ALD/MLD with three different 532 nm laser powers. b) PL intensity at 612 nm vs. 532 nm CW laser irradiation power for Sample 18 with 12.3 nm Eu-BDC thin film on TPO graphene and Si/SiO_2 substrate.

Additional FLIM Data

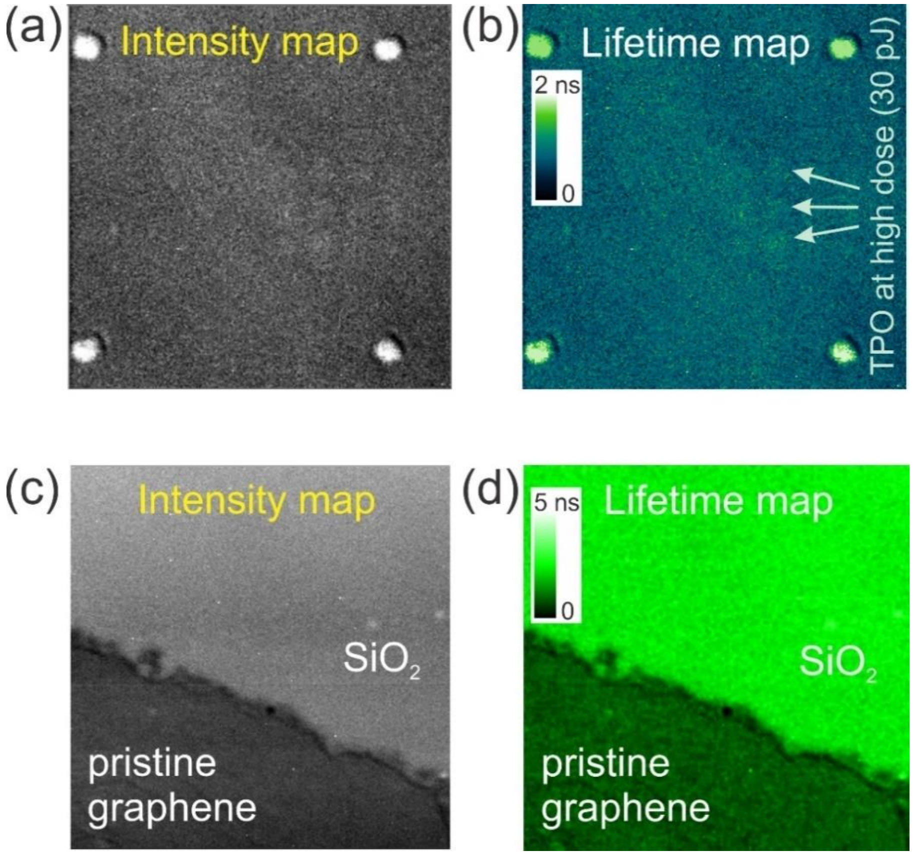

Figure S16. (a) FLIM intensity and (b) lifetime after TPO of graphene. (c) FLIM intensity and (d) lifetime after ALD/MLD on graphene and SiO_2 for Sample 18.

References

[S1] Johansson, A.; Tsai, H. C.; Aumanen, J.; Koivistoinen, J.; Myllyperkiö, P.; Hung, Y. Z.; Chuang, M. C.; Chen, C. H.; Woon, W. Y.; Pettersson, M. Chemical Composition of Two-Photon Oxidized Graphene. *Carbon* **2017**, *115*, 77-82, DOI: 10.1016/j.carbon.2016.12.091

[S2] Lucchese, M. M.; Stavale, F.; Ferreira, E. H. M.; Vilani, C.; Moutinho, M. V. O.; Capaz, R. B.; Achete, C. A.; Jorio, A. Quantifying ion-induced defects and Raman relaxation length in graphene. *Carbon* **2010**, *48* (5), 1592-1597, DOI: 10.1016/j.carbon.2009.12.057

[S3] Hu, J.; Vanacore, G. M.; Cepellotti, A.; Marzari, N.; Zewail, A. H. Rippling Ultrafast Dynamics of Suspended 2D Monolayers, Graphene. *Proc. Natl. Acad. Sci.* **2016**, *113* (43), E6555-E6561, DOI: 10.1073/pnas.1613818113

[S4] Yan, J.-A.; Chou, M. Y. Oxidation Functional Groups on Graphene: Structural and Electronic Properties. *Phys. Rev. B* **2010**, *82* (12), 125403, DOI: 10.1103/PhysRevB.82.125403

[S5] Bagri, A.; Grantab, R.; Medhekar, N. V.; Shenoy, V. B. Stability and Formation Mechanisms of Carbonyl- and Hydroxyl-Decorated Holes in Graphene Oxide. *J. Phys. Chem. C* **2010**, *114* (28), 12053-12061, DOI: 10.1021/jp908801c

[S6] Bagri, A.; Mattevi, C.; Acik, M.; Chabal, Y. J.; Chhowalla, M.; Shenoy, V. B. Structural Evolution during the Reduction of Chemically Derived Graphene Oxide. *Nat. Chem.* **2010**, *2* (7), 581-587, DOI: 10.1038/nchem.686

[S7] Ko, T. Y.; Jeong, A.; Kim, W.; Lee, J.; Kim, Y.; Lee, J. E.; Ryu, G. H.; Park, K.; Kim, D.; Lee, Z.; Lee, M. H. On-Stack Two-Dimensional Conversion of MoS2 into MoO3. *2D Mater.* **2017**, *4* (1), 014003, DOI: 10.1088/2053-1583/4/1/014003

[S8] Kang, M.; Yang, H. I.; Choi, W. Oxidation of WS2 and WSe2 Monolayers by Ultraviolet-Ozone Treatment. *J. Phys. D: Appl. Phys.* **2019**, *52* (50), 505105, DOI: 10.1088/1361-6463/ab42b0

[S9] Nye, R. A.; Van Dongen, K.; de Marneffe, J.-F.; Parsons, G. N.; Delabie, A. Quantified Uniformity and Selectivity of TiO2 Films in 45-nm Half-Pitch Patterns Using Area-Selective Deposition Supercycles. *Adv. Mater. Interfaces* **2023**, *10* (20), 2300163, DOI: 10.1002/admi.202300163

[S10] Oh, H.; Thelven, J. M.; Margavio, H. R. M.; Parsons, G. N. Low-Temperature Dual-Material Area-Selective Deposition: Molybdenum Hexafluoride–Mediated SiO_2 Fluorination/Passivation for Self-Aligned Molybdenum/Metal Oxide Nanoribbons. *Adv. Funct. Mater.* **2024**, *34* (33), 2316872, DOI: 10.1002/adfm.202316872

[S11] Prasittichai, C.; Zhou, H.; Bent, S. F. Area-Selective Molecular Layer Deposition of Polyurea Films. *ACS Appl. Mater. Interfaces* **2013**, *5* (24), 13391-13396, DOI: 10.1021/am4043195

[S12] Nguyen, C. T.; Cho, E.-H.; Gu, B.; Lee, S.; Kim, H.-S.; Park, J.; Yu, N.-K.; Shin, S.; Shong, B.; Lee, J. Y.; Lee, H.-B. Gradient Area-Selective Deposition for Seamless Gap-Filling in 3D

Nanostructures through Surface Chemical Reactivity Control. *Nat. Commun.* **2022**, *13*, 7597, DOI: 10.1038/s41467-022-35428-6

[S13] Lee, S.; Kim, M.; Baek, G.; Kim, H.-M.; Van, T. T. N.; Gwak, D.; Heo, K.; Shong, B.; Park, J. S. Thermal Annealing of Molecular Layer-Deposited Indicone Toward Area-Selective Atomic Layer Deposition. *ACS Appl. Mater. Interfaces* **2020**, *12* (38), 43212-43221, DOI: 10.1021/acsami.0c10322

[S14] Lee, S.; Kim, H.-M.; Baek, G.; Park, J.-S. Dry-Etchable Molecular Layer-Deposited Inhibitor Using Annealed Indicone Film for Nanoscale Area-Selective Deposition. *ACS Appl. Mater. Interfaces* **2021**, *13*, 60144-60153, DOI: 10.1021/acsami.1c16112

[S15] Kim, W.-H.; Hashemi, F. S. M.; Mackus, A. J. M.; Singh, J.; Kim, Y.; Bobb-Semple, D.; Fan, Y.; Kaufman-Osborn, T.; Godet, L.; Bent, S. F. A Process for Topographically Selective Deposition on 3D Nanostructures by Ion Implantation. *ACS Nano* **2016**, *10* (4), 4451-4458, DOI: 10.1021/acsnano.6b00094

[S16] Koerner, G.; Wyatt, Q. K.; Bateman, B.; Boyle, C.; Young, M. J.; Maschmann, M. R. Area-Selective Atomic Layer Deposition on HOPG Enabled by Writable Electron-Beam Functionalization. *Nano Select* **2022**, *3* (10), 1448-1457, DOI: 10.1002/nano.202200091

[S17] Yin, Y.-T.; Wu, H.-C.; Chen, M.-J.; Lee, C. S.; Liu, Y.-T.; Chen, Y.-T.; Chiu, Y.-C.; Yu, C.–C.; Chen, L.–C.; Van, T. T. N.; Chen, Y.-M.; Lee, C.–H.; Wu, K.; Lin, J. H.; Hsu, Y.–J. High-Quality HfO2 High-k Gate Dielectrics Deposited on Highly Oriented Pyrolytic Graphite via EPALS-Assisted Atomic Layer Deposition. *ACS Appl. Electron. Mater.* **2025**, *7*, 1943-1951, DOI: 10.1021/acsaelm.4c02224

[S18] Nourbakhsh, A.; Adelmann, C.; Song, Y.; Lee, C. S.; Asselberghs, I.; Huyghebaert, C.; Brizzi, S.; Tallarida, M.; Schmeißer, D.; Van Elshocht, S.; Heyns, M.; Kong, J.; Palacios, T.; De Gendt, S. Graphene Oxide Monolayers as Atomically Thin Seeding Layers for Atomic Layer Deposition of Metal Oxides. *Nanoscale* **2015**, *7*, 10781-10789, DOI: 10.1039/C5NR01128K

[S19] Lim, S.; Kim, H.; Ryu, S.; Oh, J.; Park, J.; Jung, S.; Yoon, Y. Highly Air-Stable Graphene p–n Junctions Encapsulated by Atomic Layer Deposition for Flexible and Transparent Wearable Temperature Sensors. *J. Mater. Sci. Technol.* **2025**, *233*, 104-112, DOI: 10.1016/j.jmst.2025.02.014

[S20] Park, G.; Go, D.; Jo, S.; Lee, T. H.; Shin, J. W.; Park, J. S. High-Performance Graphene-Dielectric Interface by UV-Assisted Atomic Layer Deposition. *Adv. Electron. Mater.* **2023**, *9* (7), 2300074, DOI: 10.1002/aelm.202300074

[S21] Canto, B.; Otto, M.; Maestre, A.; Centeno, A.; Zurutuza, A.; Robertz, B.; Reato, E.; Chmielak, B.; Stoll, S. L.; Hemmetter, A.; Schlachter, F.; Ehlert, L.; Li, S.; Neumaier, D.; Rinke, G.; Wang, Z.; Lemme, M. C. Multi-Project Wafer Runs for Electronic Graphene Devices in the European 2D-Experimental Pilot Line Project. *Nat. Commun.* **2025**, *16*, 1417, DOI: 10.1038/s41467-025-56357-0

[S22] Mutsukawa, R.; Takao, Y.; Murata, H.; Nagao, M.; Murakami, K. Oxidation Resistance Enhancement of Graphene-Oxide-Semiconductor Planar-Type Electron Sources Using Al2O3 Thin Protective Coating by Atomic Layer Deposition. *ACS Omega* **2025**, *10* (21), 21731-21737, DOI: 10.1021/acsomega.5c01310

[S23] Castiglione, T. R.; Pucher, T.; Dockx, K.; Contreras, G. A.; Biava, D. S.; Elchiver, B. B.; Buscema, M.; Castellanos-Gomez, A.; van der Zant, H.; Dulić, D. Air-Stable, Aluminium Oxide Encapsulated Graphene Phototransistors. *Nanotechnology* **2024**, *36* (9), 095701, DOI: 10.1088/1361-6528/ad9df0

[S24] Dlubak, B.; Kidambi, P. R.; Weatherup, R. S.; Hofmann, S.; Robertson, J. Substrate-Assisted Nucleation of Ultra-Thin Dielectric Layers on Graphene by Atomic Layer Deposition. *Appl. Phys. Lett.* **2012**, *100*, 173113, DOI: 10.1063/1.4707376

[S25] Nayfeh, O. M.; Marr, T.; Dubey, M. Impact of Plasma-Assisted Atomic-Layer-Deposited Gate Dielectric on Graphene Transistors. *IEEE Electron Device Lett.* **2011**, *32* (4), 473-475, DOI: 10.1109/LED.2011.2108258

[S26] Canto, B.; Otto, M.; Powell, M. J.; Babenko, V.; O'Mahony, A.; Knoops, H. C.; Sundaram, R. S.; Hofmann, S.; Lemme, M. C.; Neumaier, D. Plasma-Enhanced Atomic Layer Deposition of Al2O3 on Graphene Using Monolayer hBN as Interfacial Layer. *Adv. Mater. Technol.* **2021**, *6* (11), 2100489, DOI: 10.1002/admt.202100489

[S27] Dockx, K.; Barnes, M. D.; Wehenkel, D. J.; van Rijn, R.; van der Zant, H. S. J.; Buscema, M. Strong Doping Reduction on Wafer-Scale CVD Graphene Devices via Al2O3 ALD Encapsulation. *Nanotechnology* **2024**, *35* (39), 395202, DOI: 10.1088/1361-6528/ad5dbb

[S28] Kim, H.; Ryu, H.; Jeong, H. W.; Kim, J.; Moon, D.; Mun, S. A.; Hwang, C. S.; Park, M. H.; Son, J.; Lee, G.-H. Vertical Stacking of Atomic-Layer-Deposited Oxide Layers via a Fluorinated Graphene Transfer Technique. *ACS Nano* **2025**, *19* (25), 23186-23192, DOI: 10.1021/acsnano.5c04669

[S29] Riazimehr, S.; Esteki, A.; Otto, M.; Powell, M. J.; Rinke, G.; Robertz, B.; Wang, Z.; Lemme, M. C.; Hore, K.; Knoops, H. Plasma-Enhanced Atomic Layer Deposition of Al2O3 on Graphene via an In Situ-Deposited Interlayer. *Mater. Sci. Semicond. Process.* **2025**, *199*, 109829, DOI: 10.1016/j.mssp.2025.109829

[S30] Shani, L.; Lueb, P.; Menning, G.; Gupta, M.; Riggert, C.; Littmann, T.; Hackbarth, F.; Rossi, M.; Jung, J.; Badawy, G.; Verheijen, M. A.; Crowell, P. A.; Bakkers, E. P. A. M.; Pribiag, V. S. Diffusive and Ballistic Transport in Thin InSb Nanowire Devices Using a Few-Layer Graphene–AlOx Gate. *Mater. Quantum Technol.* **2024**, *4* (1), 015101, DOI: 10.1088/2633-4356/ad2d6b

[S31] Alaboson, J. M. P.; Wang, Q. H.; Emery, J. D.; Lipson, A. L.; Bedzyk, M. J.; Elam, J. W.; Pellin, M. J.; Hersam, M. C. Seeding Atomic Layer Deposition of High-k Dielectrics on Epitaxial Graphene with Organic Self-Assembled Monolayers. *ACS Nano* **2011**, *5* (6), 5223-5232, DOI 10.1021/nn201414d

[S32] Liu, X.; Yang, B.; Zhou, X.; Wu, M.; Spiecker, E.; Bachmann, J.; Hauke, F.; Hirsch, A.; Wei, T. Synergistic Combination of Reductive Covalent Functionalization and Atomic Layer Deposition-Towards Spatially Defined Graphene–Organic–Inorganic Heterostructures. *Angew. Chem. Int. Ed.* **2023**, *62*, e202314183, DOI: 10.1002/anie.202314183

[S33] Xiao, M.; Qiu, C.; Zhang, Z.; Peng, L.-M. Atomic-Layer-Deposition Growth of an Ultrathin HfO2 Film on Graphene. *ACS Appl. Mater. Interfaces* **2017**, *9* (39), 34050-34056, DOI: 10.1021/acsami.7b09408

[S34] Kim, K.; Lee, H.-B. R.; Johnson, R. W.; Tanskanen, J. T.; Liu, N.; Kim, M. G.; Pang, C.; Ahn, C.; Bent, S. F.; Bao, Z. Selective Metal Deposition at Graphene Line Defects by Atomic Layer Deposition. *Nat. Commun.* **2014**, *5*, 4781, DOI: 10.1038/ncomms5781

[S35] Mentel, K. K.; Emelianov, A. V.; Philip, A.; Johansson, A.; Karppinen, M.; Pettersson, M. Area-Selective Atomic Layer Deposition on Functionalized Graphene Prepared by Reversible Laser Oxidation. *Adv. Mater. Interfaces* **2022**, *9* (29), 2201110, DOI: 10.1002/admi.202201110

[S36] Ding, S.; Liu, Y.; Shang, Q.; Gao, B.; Yao, F.; Wang, B.; Ma, X.; Zhang, Z.; Jin, C. Morphological Evolution of Atomic Layer Deposited Hafnium Oxide on Aligned Carbon Nanotube Arrays. *Nano Lett.* **2024**, *24*, 13631-13637, DOI: 10.1021/acs.nanolett.4c03407

[S37] Brown, J. J.; Hall, R. A.; Kladitis, P. E.; George, S. M.; Bright, V. M. Molecular Layer Deposition on Carbon Nanotubes. *ACS Nano* **2013**, *7* (9), 7812-7823, DOI: 10.1021/nn402733g

[S38] Ko, J.-S.; Shearer, A. B.; Lee, S.; Neilson, K.; Jaikissoon, M.; Kim, K.; Bent, S. F.; Pop, E.; Saraswat, K. C. Achieving 1-nm-Scale Equivalent Oxide Thickness Top-Gate Dielectric on Monolayer Transition Metal Dichalcogenide Transistors With CMOS-Friendly Approaches. *IEEE Trans. Electron Devices* **2025**, *72* (1), 1-6, DOI: 10.1109/TED.2024.3466112

[S39] Kozodaev, M. G.; Lebedinskii, Y. Y.; Markeev, A. M.; Romanov, R. S.; Yakubovsky, D.; Novikov, S.; Tatmyshevskiy, V.; Volkov, V. V. Low-Energy He^+ Ions Induced Functionalization of the MoS2 Surface for ALD HfO2 Growth Enhancement. *J. Phys. Chem. C* **2023**, *127* (34), 17014-17020, DOI: 10.1021/acs.jpcc.3c00578

[S40] Mahlouji, R.; Zhang, Y.; Verheijen, M. A.; Karwal, S.; Hofmann, J. P.; Kessels, W. M. M.; Bol, A. A. Influence of High-κ Dielectrics Integration on ALD-Based MoS2 Field-Effect Transistor Performance. *ACS Appl. Nano Mater.* **2024**, *7* (16), 18786-18800, DOI: 10.1021/acsanm.4c02214

[S41] Zhang, H.; Arutchelvan, G.; Meersschaut, J.; Gaur, A.; Conard, T.; Bender, H.; Lin, D.; Asselberghs, I.; Heyns, M.; Radu, I.; Vandervorst, W.; Delabie, A. MoS2 Functionalization with a Sub-nm Thin SiO2 Layer for Atomic Layer Deposition of High-κ Dielectrics. *Chem. Mater.* **2017**, *29* (16), 6772-6780, DOI: 10.1021/acs.chemmater.7b01695

[S42] Schilirò, E.; Panasci, S. E.; Mio, A. M.; Nicotra, G.; Agnello, S.; Pécz, B.; Radnóczi, G. Z.; Deretzis, I.; La Magna, A.; Roccaforte, F.; Lo Nigro, R.; Giannazzo, F. Direct Atomic Layer Deposition of Ultra-Thin Al2O3 and HfO2 Films on Gold-Supported Monolayer MoS2. *Appl. Surf. Sci.* **2023**, *630*, 157476, DOI: 10.1016/j.apsusc.2023.157476

[S43] Šebek, M.; Wang, Z.; West, N. G.; Yang, M.; Neo, D. C. J.; Su, X.; Wang, S.; Pan, J.; Thanh, N. T. K.; Teng, J. Van der Waals Enabled Formation and Integration of Ultrathin High-k Dielectrics on 2D Semiconductors. *npj 2D Mater. Appl.* **2024**, *8*, 9, DOI: 10.1038/s41699-024-00443-2

[S44] He, Y.; Lv, Z.; Liu, Z.; Yang, M.; Ai, W.; Chen, J.; Chen, W.; Wang, B.; Fu, X.; Luo, F.; Wu, J. Sacrifice-Layer-Free Transfer of Wafer-Scale Atomic-Layer-Deposited Dielectrics and Full-Device Stacks for Two-Dimensional Electronics. *Nat. Commun.* **2025**, *16*, 5904, DOI: 10.1038/s41467-025-60864-5

[S45] Balasubramanyam, S.; Merkx, M. J. M.; Verheijen, M. A.; Kessels, W. M. M.; Mackus, A. J. M.; Bol, A. A. Area-Selective Atomic Layer Deposition of Two-Dimensional WS2 Nanolayers. *ACS Mater. Lett.* **2020**, *2* (5), 511–518, DOI: 10.1021/acsmaterialslett.0c00093

[S46] Wyndaele, P. J.; de Marneffe, J. F.; Sergeant, S.; et al. Enhancing Dielectric Passivation on Monolayer WS2 via a Sacrificial Graphene Oxide Seeding Layer. *npj 2D Mater. Appl.* **2024**, *8*, 25, DOI: 10.1038/s41699-024-00464-x

[S47] Chen, C. Y.; Sun, Z.; Torsi, R.; Wang, K.; Kachian, J.; Liu, B.; Rayner, G. B.; Chen, Z.; Appenzeller, J.; Lin, Y. C.; Robinson, J. A. Tailoring Amorphous Boron Nitride for High-Performance Two-Dimensional Electronics. *Nat. Commun.* **2024**, *15*, 4016, DOI: 10.1038/s41467-024-48429-4

[S48] Lee, T. T.; Chiranjeevulu, K.; Pedaballi, S.; Cott, D.; Delabie, A.; Dee, C. F.; Chang, E. Y. Nucleation and Growth Mechanism for Atomic Layer Deposition of Al2O3 on Two-Dimensional WS2 Monolayer. *J. Vac. Sci. Technol. A* **2023**, *41* (1), 013201, DOI: 10.1116/6.0001913

[S49] Li, X.-X.; Chen, X.-Y.; Chen, J.-X.; Zeng, G.; Li, Y.-C.; Huang, W.; Ji, Z.-G.; Zhang, D. W.; Lu, H.-L. Dual-Gate MoS2 Phototransistor with Atomic-Layer-Deposited HfO2 as Top-Gate Dielectric for Ultrahigh Photoresponsivity. *Nanotechnology* **2021**, *32* (21), 215203, DOI: 10.1088/1361-6528/abe2cc

[S50] Shearer A. B.; Ko J.-S.; Hoang A. T.; Werbrouck A.; Rothman A.; Fackovic Volcanjk D.; Lee Y.-M.; Bennett R. K. A.; Mannix A. J.; Saraswat K. C.; Pop E.; Bent S. F. Precursor Engineering of Atomic Layer Deposition for Top-Gate Insulators on Monolayer MoS2 Transistors. *ACS Nano* **2025**, *19* (37), 33473-33484, DOI: 10.1021/acsnano.5c10705

[S51] Ko, J. S.; Zhang, Z.; Lee, S.; Jaikissoon, M.; Bennett, R. K. A.; Kim, K.; Kummel, A. C.; Bandaru, P.; Pop, E.; Saraswat, K. C. Ultrathin Gate Dielectric Enabled by Nanofog Aluminum Oxide on Monolayer MoS2. In *Proceedings of the 53rd IEEE European Solid-State Device Research Conference (ESSDERC 2023)*, **2023**, 1-4, DOI: 10.1109/ESSDERC59256.2023.10268527

[S52] Xu, Y.; Liu, T.; Liu, K.; Zhao, Y.; Liu, L.; Li, P.; Nie, A.; Feng, X.; Zhuge, F.; Li, H.; Wang, X.; Zhai, T. Scalable Integration of Hybrid High-k Dielectric Materials on Two-Dimensional Semiconductors. *Nat. Mater.* **2023**, *22*, 1078-1084, DOI: 10.1038/s41563-023-01626-w.

[S53] Li, W.; Zhou, J.; Cai, S.; Yu, Z.; Zhang, J.; Fang, N.; Li, T.; Wu, Y.; Chen, T.; Xie, X.; Ma, H.; Yan, K.; Dai, N.; Wu, X.; Zhao, H.; Wang, Z.; He, D.; Pan, L.; Shi, Y.; Wang, P.; Chen, W.;

Nagashio, K.; Duan, X.; Wang, X. Uniform and Ultrathin High-k Gate Dielectrics for Two-Dimensional Electronic Devices. *Nat. Electron*. **2019**, *2*, 563-571, DOI: 10.1038/s41928-019-0334-y

[S54] Sohn, I.; Wi, S.; Kim, Y.; Shin, D.; Kim, M.; Lee, S.; Yoon, H.; Yoo, J.; Chung, S.-M.; Ki, H. Selective Passivation of 2D TMD Surface Defects by Atomic Layer Deposited Al2O3 to Enhance Recovery Properties of Gas Sensor. *Appl. Surf. Sci.* **2024**, *646*, 158906, DOI: 10.1016/j.apsusc.2023.158906

[S55] Park, J.; Kwak, S. J.; Kang, S.; Oh, S.; Shin, B.; Noh, G.; Kim, T. S.; Kim, C.; Park, H.; Oh, S. H.; Kang, W.; Hur, N.; Chai, H. J.; Kang, M.; Kwon, S.; Lee, J.; Lee, Y.; Moon, E.; Shi, C.; Lou, J.; Lee, W. B.; Kwak, J. Y.; Yang, H.; Chung, T. M.; Eom, T.; Suh, J.; Han, Y.; Jeong, H. Y.; Kim, Y. J.; Kang, K. Area-Selective Atomic Layer Deposition on 2D Monolayer Lateral Superlattices. *Nat. Commun.* **2024**, *15*, 2138, DOI: 10.1038/s41467-024-46293-w

[S56] Khan, R.; Shong, B.; Ko, B. G.; Lee, J. K.; Lee, H.; Park, J. Y.; Oh, I. K.; Raya, S. S.; Hong, H. M.; Chung, K. B.; Luber, E. J.; Kim, Y. S.; Lee, C. H.; Kim, W. H.; Lee, H. B. R. Area-Selective Atomic Layer Deposition Using Si Precursors as Inhibitors. *Chem. Mater.* **2018**, *30* (21), 7603-7610, DOI: 10.1021/acs.chemmater.8b02774
